# Supplementary material for: A unified model for interpretable latent embedding of multi-sample, multi-condition single-cell data
Source: Nat Commun. 2024 Aug 3;15:6573. doi: 10.1038/s41467-024-50963-0 (PMC11298001; doi:10.1038/s41467-024-50963-0)
Supplement: Supplementary file 1 — Supplementary Information [file 41467_2024_50963_MOESM1_ESM.pdf]

Supplementary Information

SUPPLEMENTARY FIGURES AND TABLES **3**

SUPPLEMENTARY METHODS **25**

1 THE GEDI FRAMEWORK 25
1.1 Fitting the GEDI model with no prior gene-level or sample-level information 25
1.1.1 Solving $\mathbf{Z}_r$ 25
1.1.2 Solving $\Delta\mathbf{Z}_i$ 27
1.1.3 Solving $\mathbf{o}_r$ 27
1.1.4 Solving $\Delta\mathbf{o}_i$ 27
1.1.5 Solving **B** 28
1.1.6 Solving σ^2 28
1.2 Fitting the GEDI model with gene-level prior information 29
1.2.1 Solving **A** 29
1.2.2 Solving σ^2 30
1.2.3 Solving other parameters 32
1.3 Fitting the GEDI model with sample-level prior information 32
1.3.1 Solving $\mathbf{R}_o$ 34
1.3.2 Solving $\mathbf{R}_k$ 34
1.3.3 Solving σ^2 35
1.3.4 Solving other parameters 35
1.4 Fitting the GEDI model to UMI counts 35
1.4.1 Solving parameters other than σ^2 36
1.4.2 Solving σ^2 37
1.5 Fitting the GEDI model to paired UMI counts 37
1.5.1 Obtaining the expectation of **Y** 38
1.5.2 Obtaining the variance of **Y** 39
1.6 Choice of hyperparameters 40
1.6.1 The prior distribution of $\mathbf{Z}_r$ 40
1.6.2 The prior distribution of $\Delta\mathbf{Z}_i$ 40
1.6.3 The prior distribution of $\Delta\mathbf{o}_i$ 40
1.6.4 The prior distribution of **A** 41
1.6.5 The prior distributions of $\mathbf{R}_o$ and $\mathbf{R}_k$ 41
2 DATASETS AND PREPROCESSING 42
2.1.1 PBMC dataset 42
2.1.2 Pancreas dataset 42
2.1.3 Tabula Muris BM dataset 42
2.1.4 COVID-19 dataset 42
2.1.5 Faure dataset 43
2.1.6 La Manno dataset 43
2.1.7 Genga dataset 43
2.1.8 Tasic dataset 43
3 INTEGRATION METHODS 44
3.1.1 GEDI 44
3.1.2 Seurat 44
3.1.3 LIGER 44
3.1.4 Harmony 44
3.1.5 BBKNN 44
3.1.6 CSS 45
3.1.7 PCA 45
3.1.8 scVI 45
3.1.9 Scanorama 45
4 METRICS TO COMPARE INTEGRATION PERFORMANCE 45
4.1.1 Alignment Score 45
4.1.2 LISI 46
4.1.3 kBET 46
4.1.4 ASW 46
4.1.5 ARI 46

4.1.6 NMI ... 46
5 SIMULATING COHORT-LEVEL SCRNA-SEQ DATA ... 47
5.1 A generative model for simulating single-cell data ... 47
5.2 Selecting the parameters of the generative model ... 48
5.2.1 Estimating w_n ... 48
5.2.2 Estimating X_g and x'_g ... 48
5.2.3 Estimating Σ_g: ... 49
5.2.4 Estimating σ_g: ... 49
5.2.5 Estimating M_n: ... 50
5.3 Cluster-free differential expression benchmark ... 50
5.3.1 GEDI ... 50
5.3.2 LEMUR ... 50
5.3.3 miloDE ... 50
5.3.4 Vector field visualization ... 50
SUPPLEMENTARY REFERENCES ... 51

Supplementary Figures and Tables

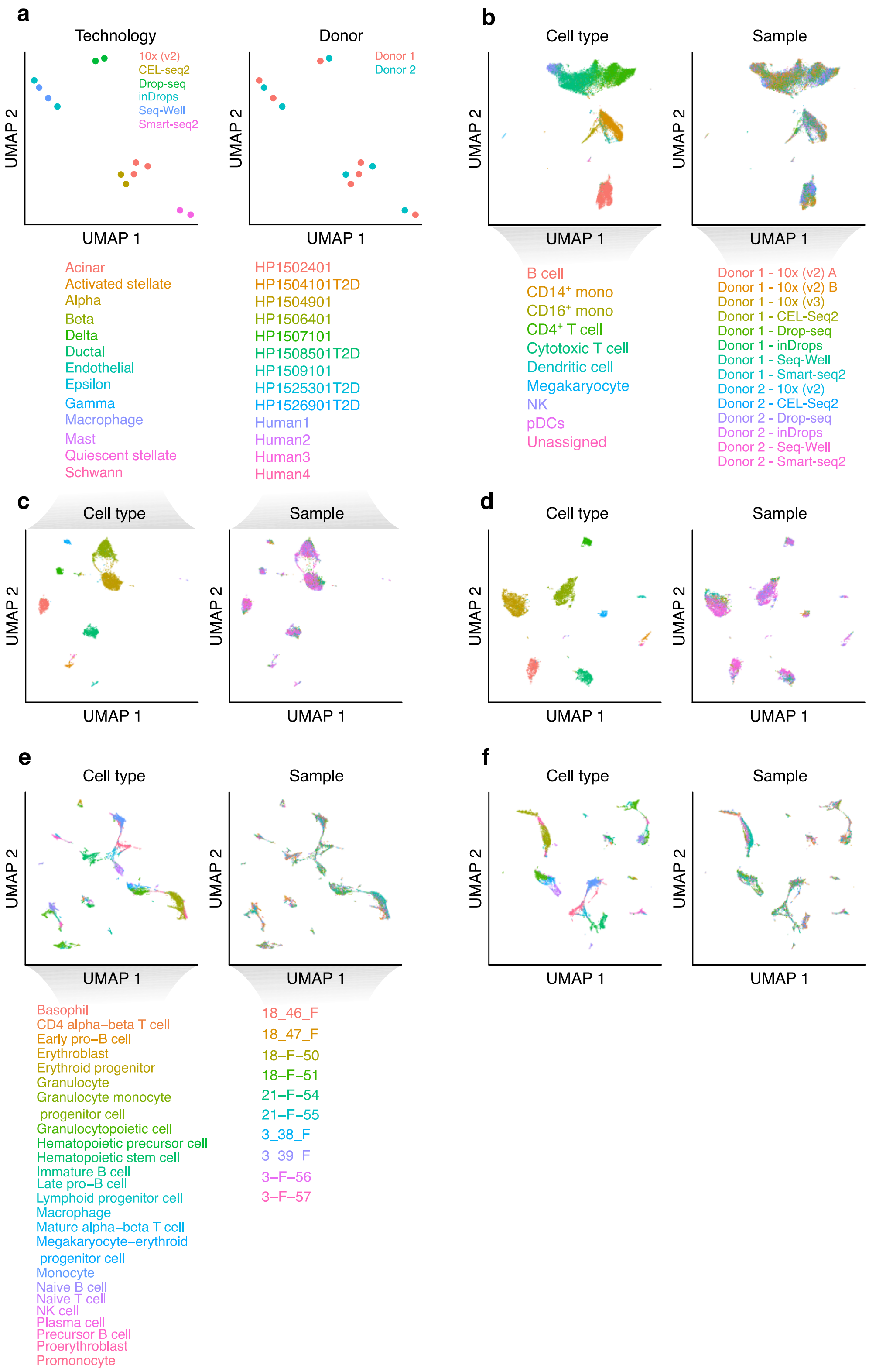

Supplementary Figure 1. Analysis of sample-to-sample variability with GEDI
(Continued on the next page)

(**a**) UMAP embedding of the sample-specific manifold distortions learned by GEDI for the PBMC dataset[1] (related to **Fig. 2a**). For each sample i, GEDI learns a set of sample-specific manifold parameters, consisting of $\Delta\mathbf{Z}_i$ and $\Delta\mathbf{o}_i$ (see **Methods** for the explanation of these parameters). We concatenated $\Delta\mathbf{Z}_i$, followed by vectorization, to obtain a vector $\boldsymbol{\theta}_i \in \mathbb{R}^{G(K)}$ for each sample i, where G is the number of genes and K is the number of principal axes (see **Methods**). We then regressed out the effect of donor from each element j of each $\boldsymbol{\theta}_i$ by modeling $\theta_{i,j} \sim \alpha_j + \beta_j h_i$ across the samples and taking the residual of the regression (here, h_i represents the donor for sample i). We then performed PCA and UMAP dimensionality reduction on the residuals to obtain the plots shown here. Each dot represents one sample, labeled by the single-cell technology used (left) or donor of origin (right). Only technologies with more than one sample are displayed. A similar analysis was performed to obtain **Fig. 2a**, with the difference that in that figure, the effect of technology was regressed out (h_i was set to the technology used for each sample i) (**b**) UMAP embedding of the cells in the PBMC dataset after integration with GEDI (hyperplane mode). Each dot represents one cell, colored by the cell type labels from the original study (left) or by sample (right). This figure is related to **Fig. 2b**, with the difference that **Fig 2b** is based on GEDI in the hyperellipsoid mode. (**c**) UMAP embedding of the cells in the Pancreas dataset[2, 3] after integration with GEDI (hyperellipsoid mode). Each dot represents one cell, colored by the cell type labels from the original study (left) or by sample (right). (**d**) Same as (c), but integration was performed with GEDI (hyperplane mode). (**e**) UMAP embedding of the cells in the Tabula Muris dataset[4] after integration with GEDI (hyperellipsoid mode). Each dot represents one cell, colored by the cell type labels from the original study (left) or by sample (right). (**f**) Same as (e), but integration was performed with GEDI (hyperplane mode).

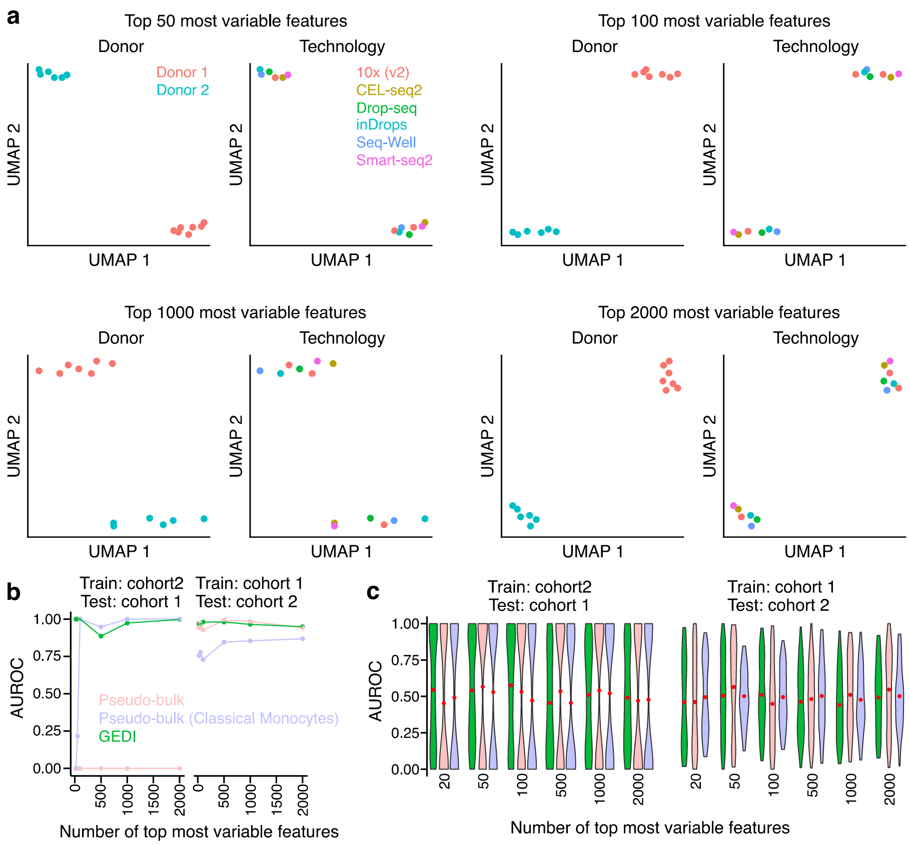

Supplementary Figure 2. Effect of the number of variable features on analysis of sample-to-sample variability (**a**) UMAP embeddings of the sample-specific manifold distortions learned by GEDI for the PBMC dataset[1] (related to **Fig. 2a**); The panels are similar to **Supplementary Figure 1a**, but different number of most variable features were selected prior to PCA and UMAP embedding. Specifically, we selected the most variable features of the residuals, followed by PCA and UMAP dimensionality reduction to obtain the plots shown here. (**b**) Analysis of the effect of the number of features used for assessing the classification between COVID and control cases in the COVID-19 dataset (related to **Fig. 2e**). For the classification task, a Support Vector Machine (SVM) was trained using different number of most variable features from either: 1) the sample-specific manifold distortion learned by GEDI; 2) the pseudo-bulk of all cell-types or 3) the pseudo-bulk of classical monocytes. For the GEDI analysis, samples were first encoded using the sample-specific manifold parameters, while for the pseudo-bulk analysis the variance stabilizing transformation from DESeq2 was applied on the aggregated count data. We then regressed out the effect of cohort from each feature, selected different numbers of the most variable features (shown on the x-axis) from the residual matrices, and trained a SVM using the PCA embedding from the residual matrices. Performance of classification was measured using the area under the receiving operating characteristic curve (AUROC) values. Left: SVM was trained with data from cohort 2 and tested on cohort 1. Right: SVM was trained with cohort1 and tested on cohort2. (**c**) Random baseline of the COVID-19 status classification task using either GEDI distortion matrices, a pseudobulk using all cell types or a pseudo-bulk using the classical monocytes. To generate the random baseline, 100 models were evaluated where the sample labels were shuffled randomly during training, followed by testing the model on real sample labels of the test cohort. Other steps are the same as in (b). Violin plots show the distribution of the AUROC scores across various number of most variable features, while the red dot represents the mean AUROC score per method.

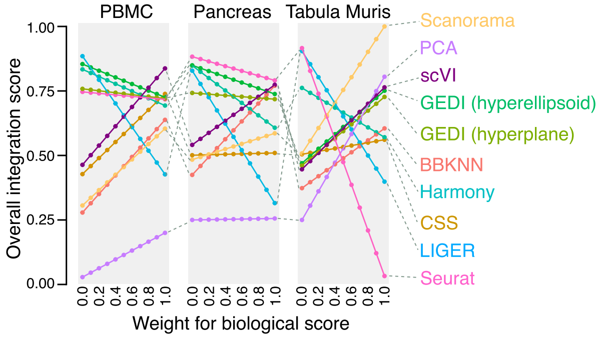

Supplementary Figure 3. The effect of the choice of weights on the overall integration score

Overall ranking score comparing the performance of various integration methods over a range of weights for the biological score, applied to the PBMC, Pancreas and Tabula Muris datasets (related to **Figure 2c**). The overall integration score is calculated based on a weighted sum of the 'batch score' and 'biological score' (see **Methods** for details). The original integration score[5] used a weight of 0.6 for the biological score and 0.4 for the batch score. In this plot, the overall ranking score is recomputed by modifying the weight of the biological score. Source data are provided as a Source Data file.

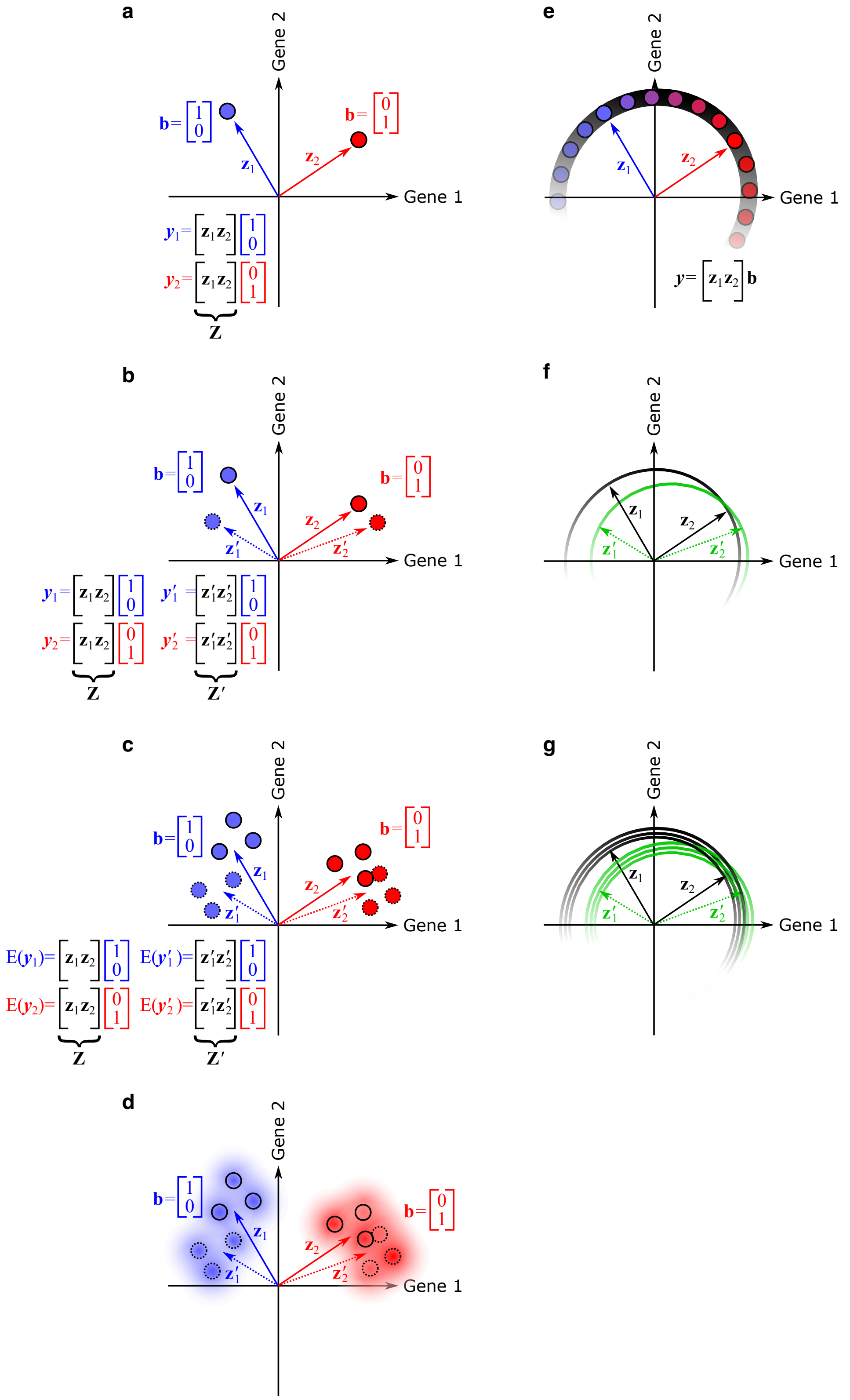

Supplementary Figure 4. Schematic representation of the transcriptomic vector field and its comparison to traditional DGE approaches

(Continued on the next page)

(**a-c**) Differential expression analysis between conditions at the level of cell clusters. (**a**) Schematic of the gene expression profiles of two clusters y_1 and y_2. The coordinates of each dot represent the gene expression measurements of gene 1 vs gene 2, while **b** represents the identity of each cluster, given by a one-hot encoding vector. The vectors $\mathbf{z}_1$ and $\mathbf{z}_2$ represent the principal axes of the gene-expression data (in this case, the expression vector of each cluster). The matrix multiplications show how the expression profile of each cluster can be obtained knowing the cell state **b** and the matrix of principal axes **Z**. (**b**) Same as (a) but showing the gene expression profiles of two clusters y_1 and y_2 in two conditions. Dotted circles represent the gene expression measurements in the new condition, while $\mathbf{z}'_1$ and $\mathbf{z}'_2$ represent distortions to the principal axes introduced by the condition-associated changes. The matrix multiplications show that the expression profiles of each cluster in each condition can be obtained by knowing the cell state **b** and the matrices **Z** and $\mathbf{Z}'$, which can then be used to calculate the change in expression from one condition to the other. (**c**) Same as (b) but including multiple samples per condition, with sample-to-sample variability in each condition in addition to condition-to-condition variability. The diagram shows how in pseudobulk cluster-based DGE approaches, gene-expression data is aggregated for each cluster and sample, which is then used to estimate the change in the average expression of each cell cluster between conditions while accounting for unexplained inter-sample variability. (**d**) Other methods also exist[6] that consider each single cell individually, as opposed to using pseudobulks; the fuzzy circles represent the cell-to-cell variability within each cluster in each condition. Calculation of the change in expression between conditions is still performed per cluster but considering each cell as one observation, accounting for inter-sample variability and within-sample variability of cells. (**e-g**) Cluster-free differential expression analysis between conditions. (**e**) Same as (a), however **b**, the biological state, is not represented by a one-hot vector but rather by a continuum of cell-states. The principal axes $\mathbf{z}_1$ and $\mathbf{z}_2$ define a manifold near which different cell-states lie. (**f**) Same as (e) but showing that when comparing two conditions, instead of being interested in how the expression vectors of two clusters change, we are interested in finding how the manifold of the cells changes between two conditions. (**g**) Same as (e) but including multiple samples per condition. GEDI models inter-sample variability of the manifolds as well as within-sample variability of cells around the manifold.

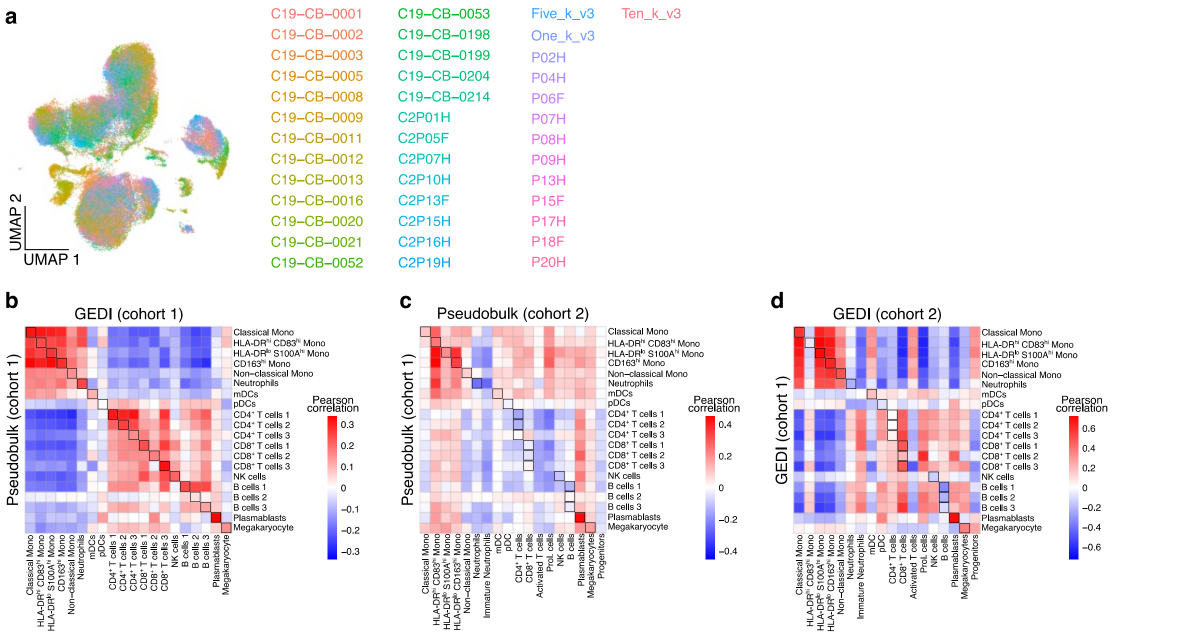

Supplementary Figure 5. Additional comparisons between GEDI and pseudo-bulk analysis
(**a**) UMAP embedding of the COVID-19 dataset[7] for cohort 1 (similar to **Fig. 3b**). The color indicates the donor labels from the original study. (**b**) Comparison between the mean transcriptomic vector per cell type, obtained from GEDI, and differential gene expression values (log fold-change) obtained from pseudo-bulk analysis, for the comparison of severe COVID-19 vs. control cases in cohort 1 (related to **Fig. 3e**). Heatmap shows the Pearson correlation values between GEDI (columns) and pseudo-bulk analysis (rows). (**c**) Same as in (b) but showing reproducibility between cohort 1 (rows) and cohort 2 (columns) for the pseudo-bulk analysis (related to **Fig. 3f**). (**d**) Same as in (b-c) but showing reproducibility between cohort 1 (rows) and cohort 2 (columns) for GEDI (related to **Fig. 3g**).

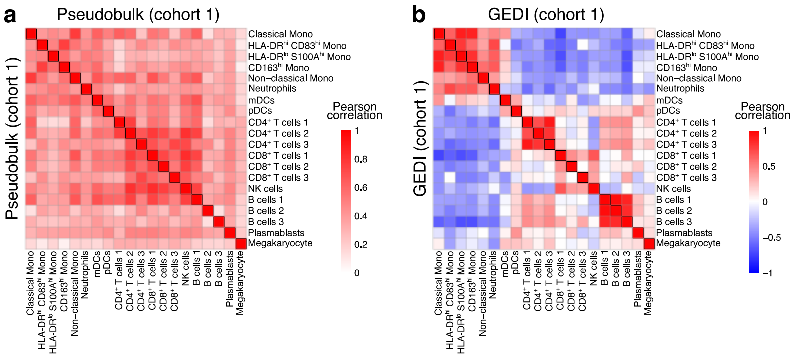

Supplementary Figure 6. GEDI differential expression estimates preserve cell type-specificity
(**a**) Pseudo-bulk differential expression (DE) analysis between mild COVID-19 vs. control in cohort 1. Heatmap shows the Pearson correlation for the estimated DE profiles of different cell types. (**b**) same as (a) but showing the Pearson correlation of the mean transcriptomic vectors of the cell types, obtained from GEDI.

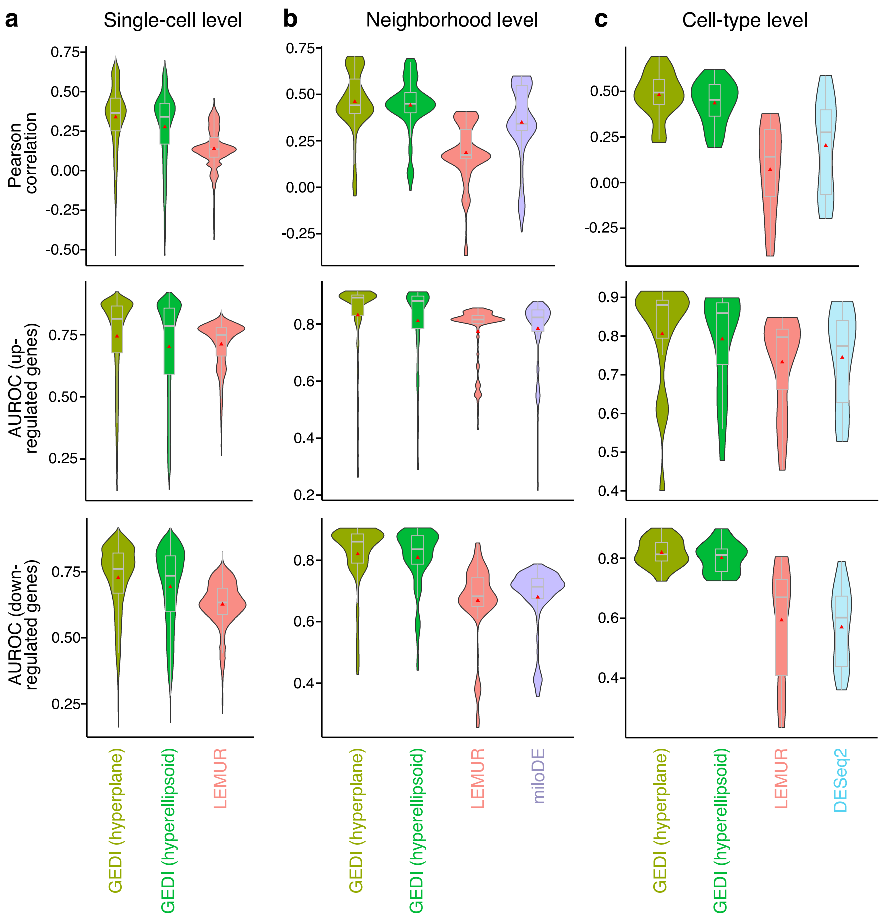

Supplementary Figure 7. Additional comparisons of cluster-free DGE methods
(**a**) Single-cell level comparison of GEDI and LEMUR, using a simulated cohort-level single-cell dataset. To generate the simulated cohort-level single-cell dataset, the COVID-19 dataset was used as a template to select the parameters of the generative model, based on the comparison of severe COVID-19 vs. control cases in cohort1. For each cell (n of cells = 86,549), the differential expression estimate of a given method was compared against the ground truth vectors via Pearson correlation (top), or by assessing the classification of up-regulated (middle) or down-regulated (bottom) genes using AUROC scores. Sets of up-regulated and down-regulated genes were defined by using a threshold of 0.3 (log_2 scale) of the ground truth DE values. For each metric, the violin plots and boxplots show the distribution across all cells; red triangle: mean, center line: median; box limits: upper and lower quartiles; whiskers: 1.5x the interquartile range (related to **Fig 4b**). (**b**) Same as in (a) but comparing DE estimates at the neighborhood level (n of neighborhoods = 215) and including miloDE. Ground truth vectors and GEDI's and LEMUR's single-cell DE estimates were collapsed into neighborhood estimates, by averaging across cells of the neighborhoods defined by miloDE (related to **Fig 4c**). **(c)** Same as in (a) but comparing DE estimates at the cell type level (n of cell types = 20) and including DESeq2. Ground truth vectors and GEDI's and LEMUR's single-cell DE estimates were collapsed into cell type estimates, by averaging across cells of the same cell type (related to **Fig 4d**).

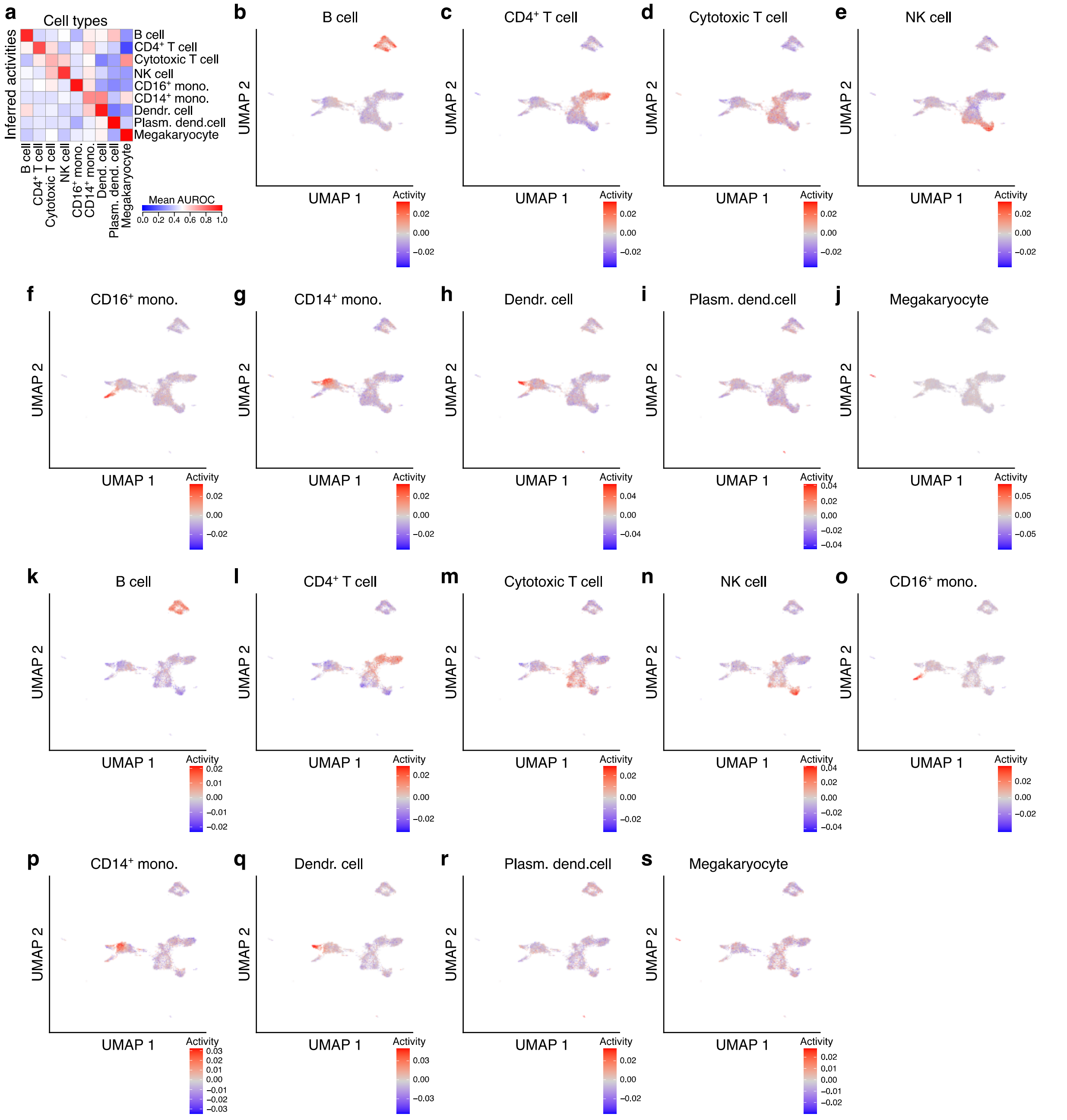

Supplementary Figure 8. Projection of cell type signatures with GEDI
(**a**) Cell type signature projections obtained by GEDI are compared to the true labels in the PBMC dataset (donor 2). Heatmap shows AUROC values for differential enrichment of inferred cell type signatures from GEDI (rows) for each cell type (columns). This figure is similar to **Fig. 5a**, with the difference that, here, the gene signatures are obtained from donor 1, followed by their projection on the cells from donor 2. (**b-j**) UMAP plots showing single-cell projection of cell-type signature activities for donor 1. (**k-s**) UMAP plots showing single-cell projection of cell-type signature activities for donor 2. Source data are provided as a Source Data file.

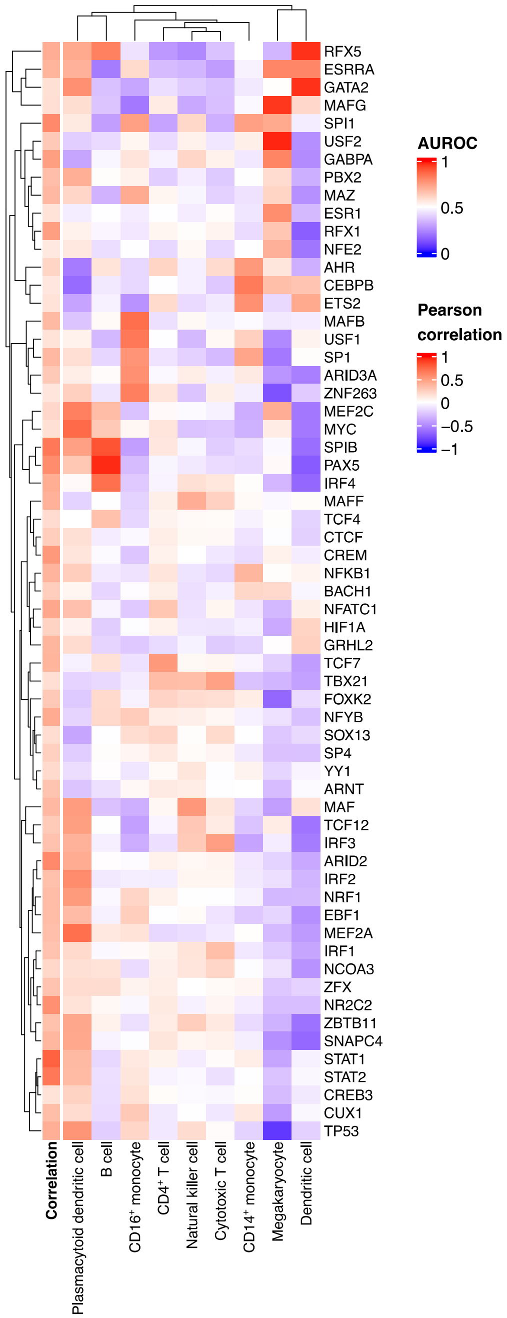

Supplementary Figure 9. Identification of cell type-specific transcription factor activities with GEDI
Heatmap shows AUROC values for differential enrichment of inferred transcription factor (TF) activities from GEDI (rows) for each cell type (columns) in the PBMC dataset. Left annotation heatmap shows the Pearson correlation between inferred activity and model-fitted mRNA abundance. For this figure, we included only TFs with Pearson correlation >0.1 between activity and model-fitted mRNA abundance. See **Supplementary Data 2** for mean AUROC values per cell type for all TFs analyzed.

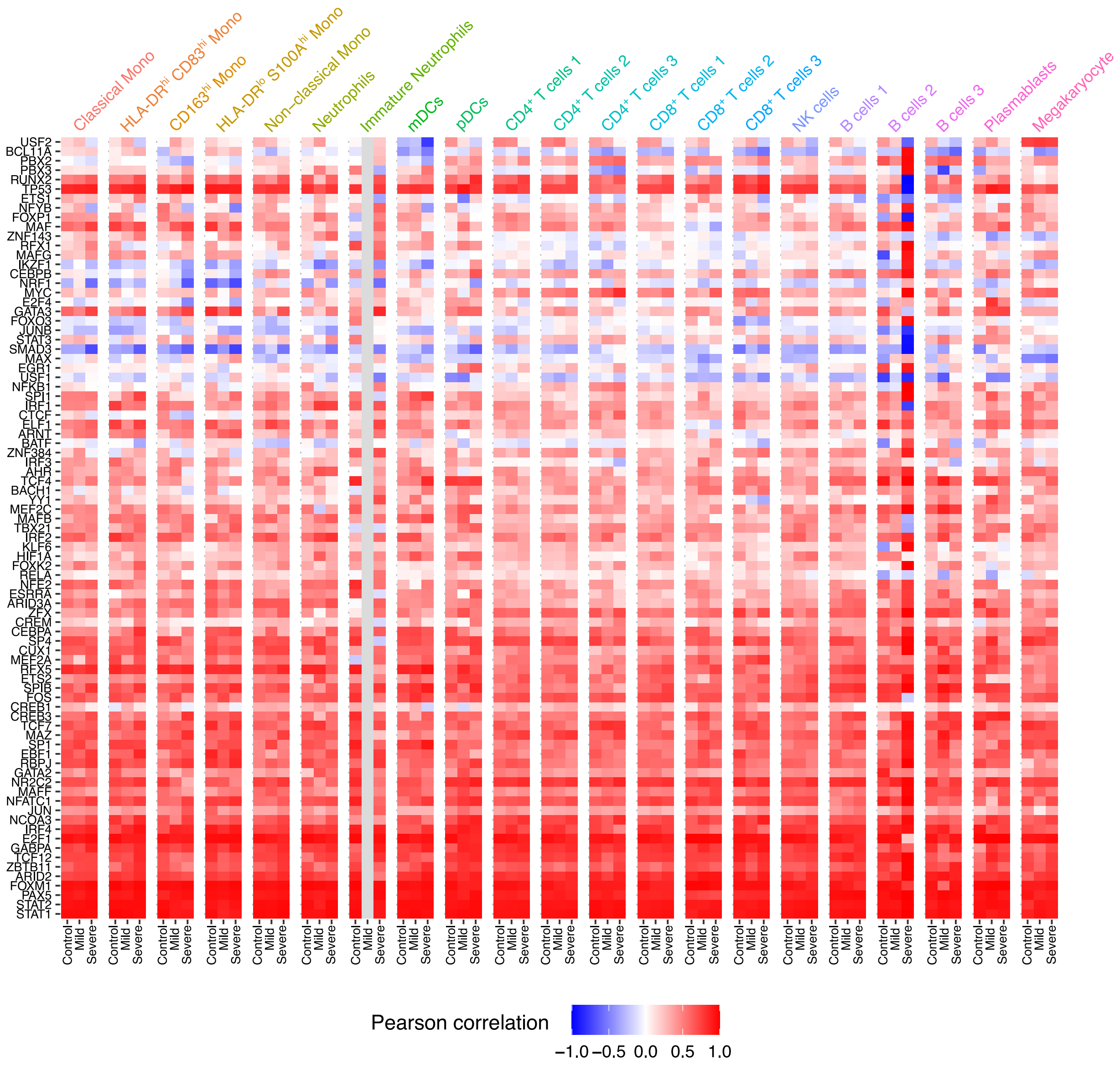

Supplementary Figure 10. Comparison between the projected TF regulon activities and TF mRNA abundance
The heatmap shows the Pearson correlation between the inferred regulon activity of each TF and the GEDI-imputed abundance of the mRNA encoding that TF, separately calculated for each cell type and each COVID-19 condition in the COVID-19 cohort 1 dataset.

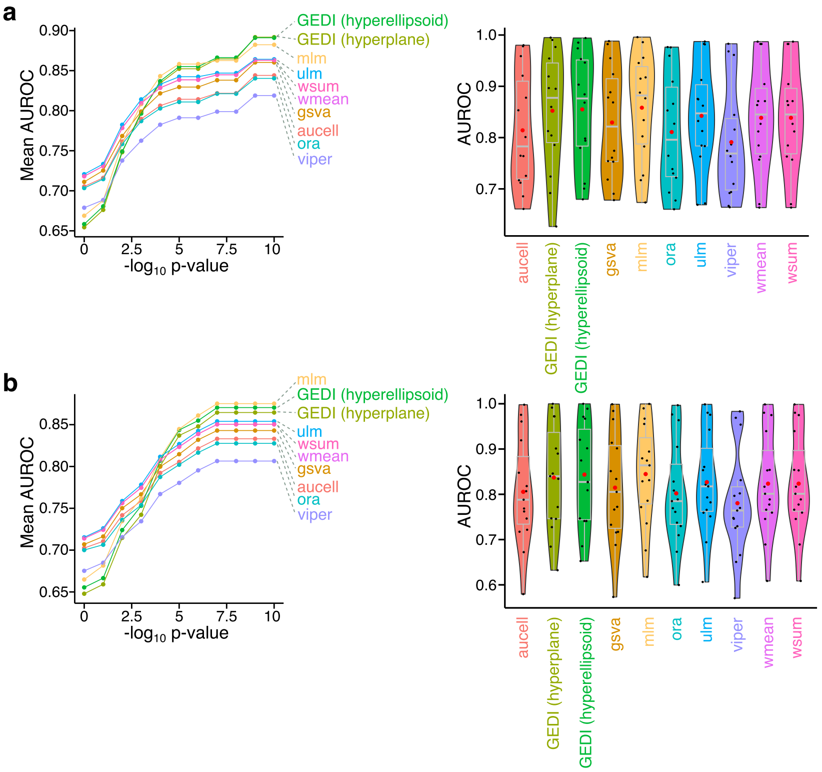

Supplementary Figure 11. Assessment of estimated TF activities using a single-cell perturbation dataset
(**a**) Comparison of different methods for the inference of TF activity using the CRISPRi dataset from Genga et al[8] as ground truth. The CRISPRi dataset consisted of two technical batches, which were split in our analysis. First, the degree of association between the perturbation of each TF and the principal axes of heterogeneity of batch 1 cells were identified using a likelihood-ratio test (shown on the x-axis of the left plot; see **Methods** for details). Next, single cell TF activities were estimated for each method in cells from batch 2. Then, for each TF and each method, the estimated activities were evaluated as a classifier to predict TF perturbation status. Performance of the classification was measured using AUROC values (y-axes). Left: Mean AUROC scores for sets of TFs with varying degree of association with the principal axes of heterogeneity, as shown by the different p-value cutoffs from the likelihood ratio test (each dot represents the sets of TFs that pass the significance threshold depicted by the x-axis). Right: Violin and boxplots showing the distribution of AUROC scores for the set of TFs that pass the the $1x10^{-5}$ p-value cutoff. Black dots represent the AUROC score of each TF while the red dot represents the mean AUROC for each method. (**b**) Same as (a) but batch 2 cells were used for testing the association of each TFs with the principal axes of heterogeneity, and batch 1 cells were used for evaluating the performance of classification. Source data are provided as a Source Data file.

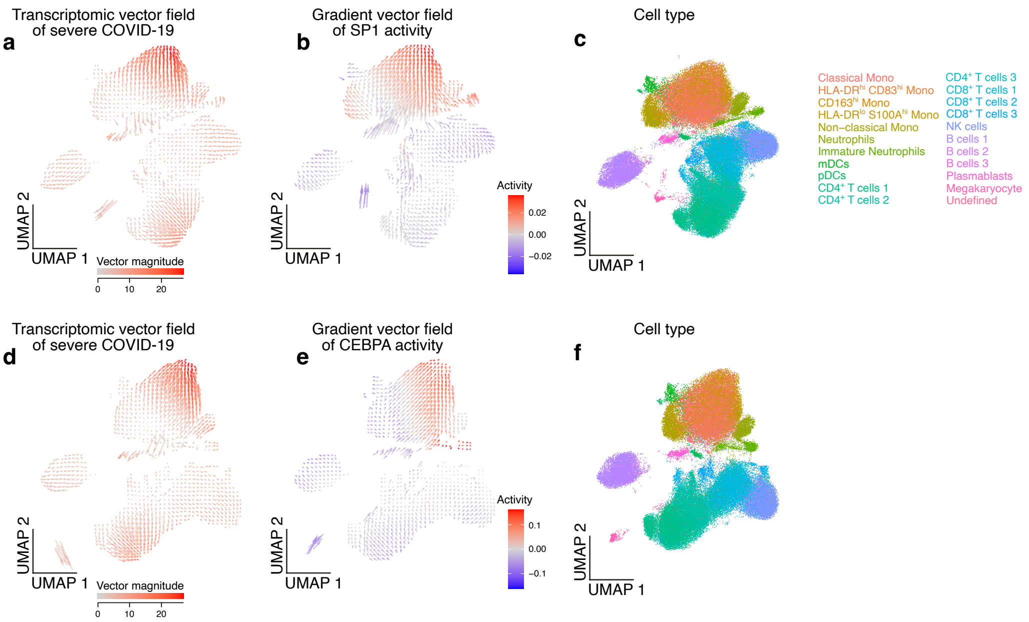

Supplementary Figure 12. Correlation of TF activity gradient vectors and the transcriptomic vector field (**a-c**) The activity gradient of SP1 correlates with the transcriptomic vector of severe COVID-19 in monocytes. (**a**) UMAP representation of the transcriptomic vector field of severe COVID-19. The color shows the vector magnitude. (**b**) Gradient vector field of SP1 activity. The color represents SP1 activity. (**c**) The same UMAP as in (a-b), but the color represents the cell type labels as a reference. (**d-f**) same as in (a-c), but for CEBPA. (**d**) Same as (a), but UMAP coordinates are derived from the analysis of the vector field of CEBPA. (**e**) Same as (b) but showing CEBPA activity. (**f**) Same as (c), but UMAP coordinates are derived from the analysis of the vector field of CEBPA. Also see **Fig. 5d-g**.

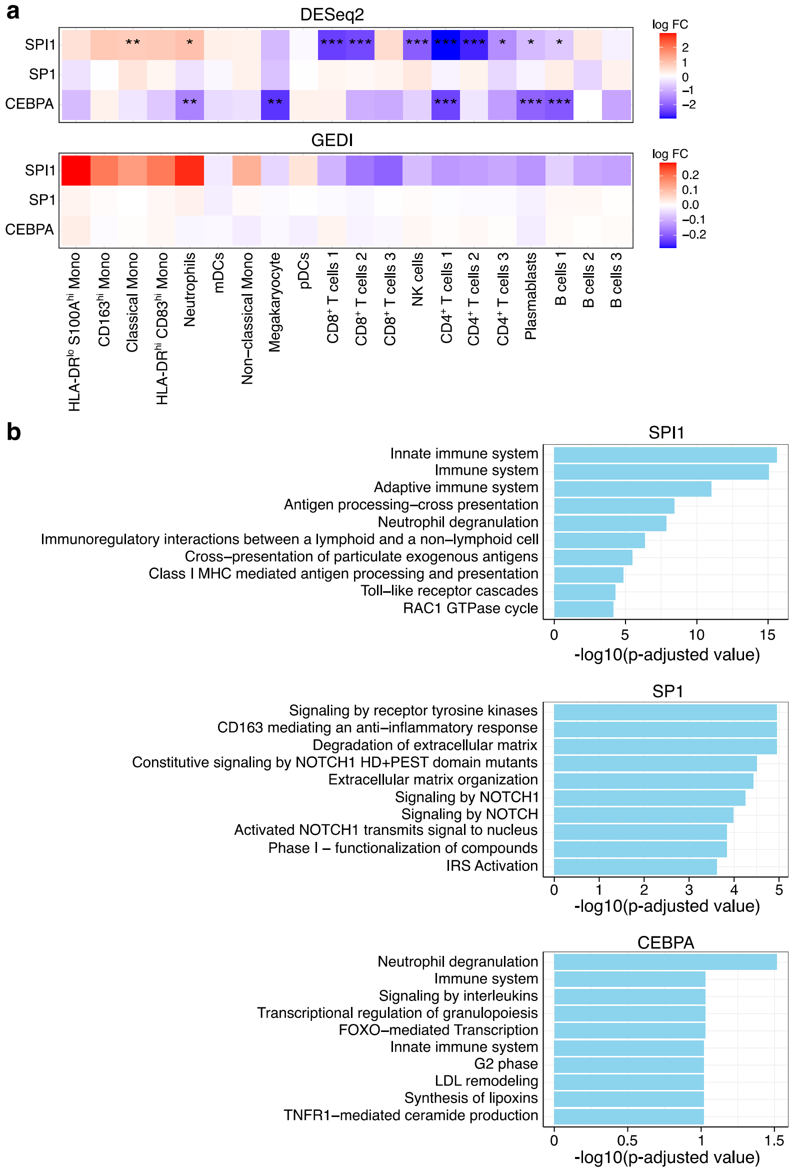

Supplementary Figure 13. TFs whose activity gradients are associated with severe COVID-19
(**a**) Differential expression of SPI1, SP1 and CEBPA, some of the TFs whose activity gradients show high concordance with the transcriptomic vector field of severe COVID-19 (related to **Fig. 5d-g** and **Supplementary Figure 12**). Heatmap shows log fold changes per cell type from DESeq2 (top) or the mean transcriptomic vector field from GEDI (bottom). For DESeq2, * denotes p-adjusted value <0.05, ** denotes p-adjusted value <0.01, *** denotes p-adjusted value <0.001 and no symbol represents p-adjusted value ≥0.05. Exact p-values are provided in the Source Data file. (**b**) Enrichment analysis of the top targets of SPI1, SP1 and CEBPA. For each TF we obtained the Pearson correlation between inferred activity from GEDI and model-fitted abundance of each gene, and kept the 50 top genes with the highest correlation. Then, for each TF, we overlapped the list with the TF targets defined by the refined human DoRothEA gene-regulatory network, restricting for activating interactions. The final gene set was used as input to Enrichr[9] and enrichment analysis was performed using the Reactome 2022 library. Bar height indicates the negative $\log_{10}$ of the Benjamini-Hochberg adjusted p-values. Source data are provided as a Source Data file.

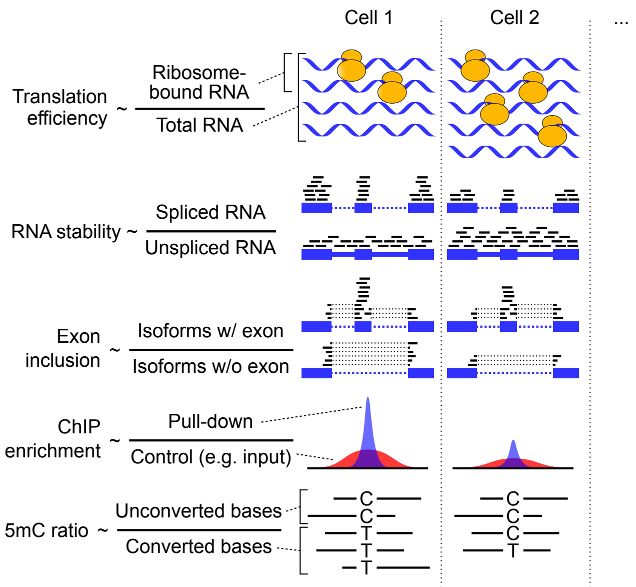

Supplementary Figure 14. Examples modalities that are defined as the ratio of two quantities.

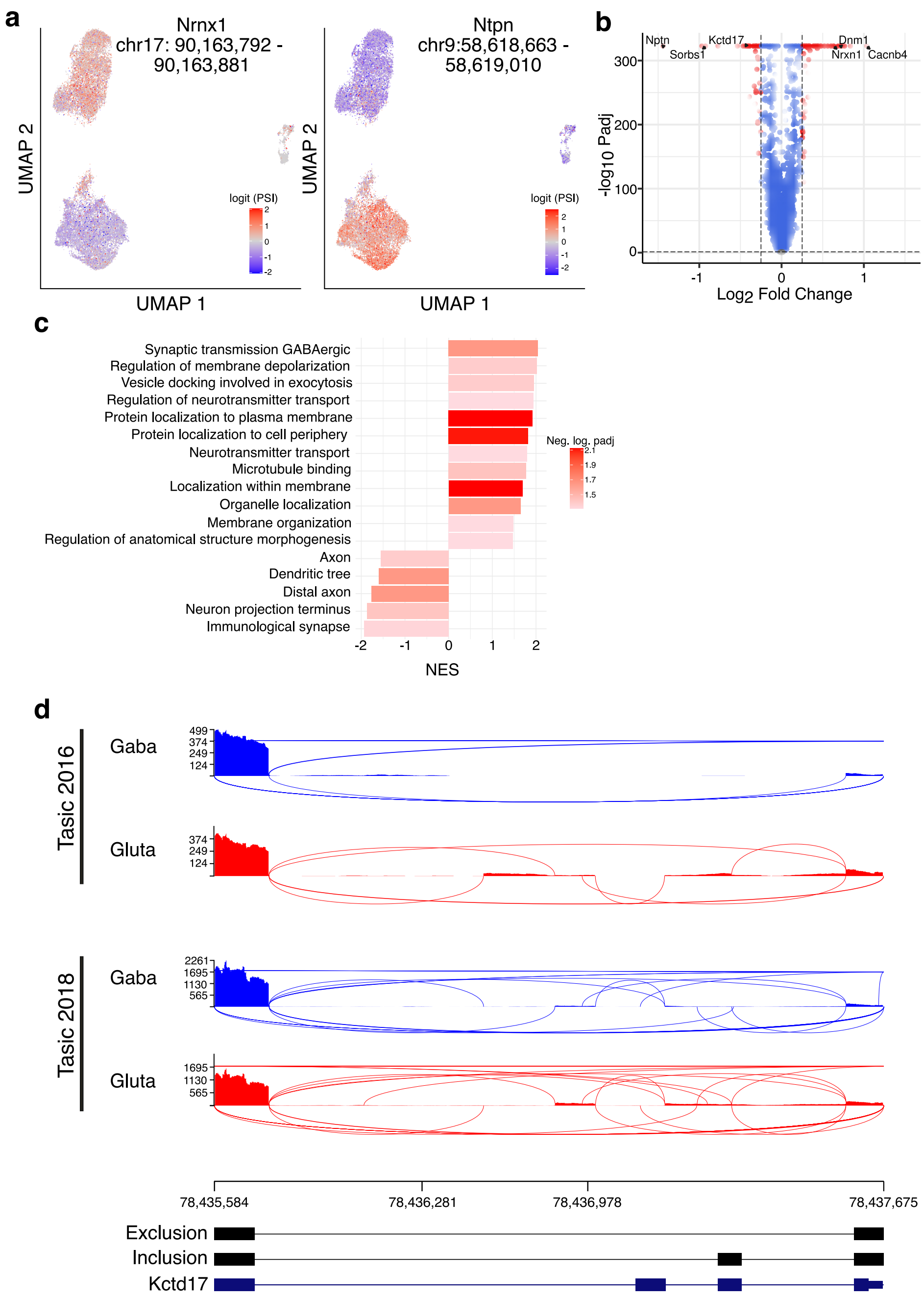

Supplementary Figure 15. Analysis of the latent splicing space learned by GEDI
(**a**) UMAP embedding of the latent splicing space of mouse cortical cells after integration of data from two studies[10, 11]. This figure is similar to **Fig. 6d**, with the difference that the color represents the log-odds of inclusion/exclusion for a cassette exon in *Nrnx1* (left) and a cassete exon in *Ntpn* (right). (**b**) Volcano plot showing differential PSI between GABAergic and Glutamatergic neurons. Red points indicate exon inclusion events with a $\log_2$ fold-change >0.25. (**c**) Gene Set Enrichment Analysis (GSEA[12]) of differential exon inclusion between GABAergic and Glutamatergic neurons. Bar height indicates the normalized enriched score while bar color represents the negative $\log_{10}$ of the FDR-adjusted p-value. (**d**) Sashimi plots of an example cassette exon in *Kctd17* that is differentially spliced between neuronal subtypes. Source data are provided as a Source Data file.

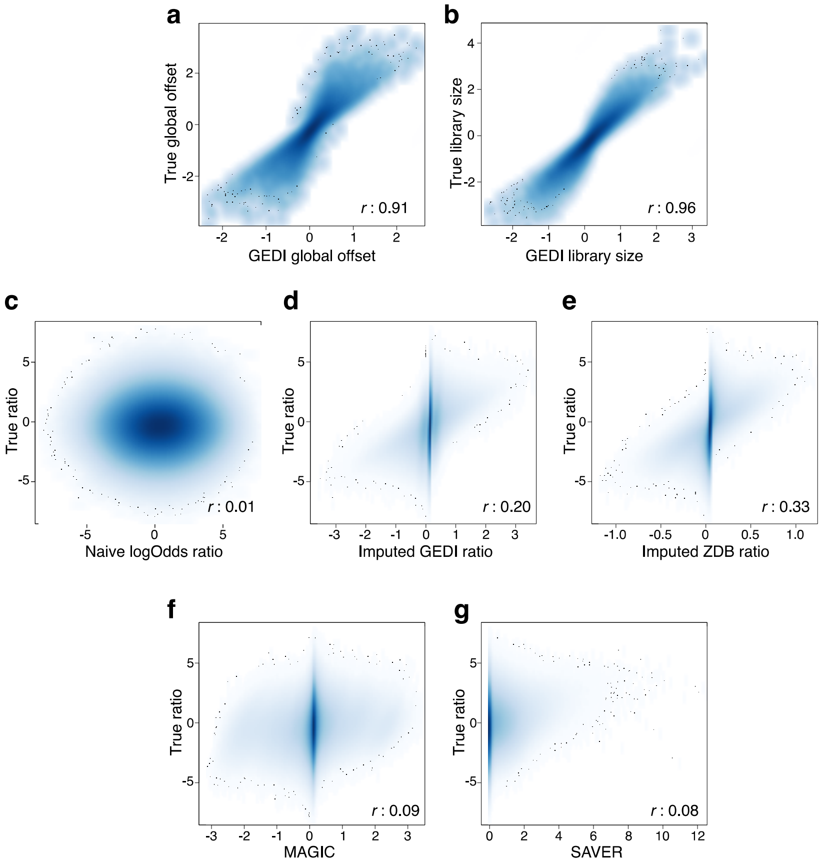

Supplementary Figure 16. Analysis of simulated pairs of UMI counts with GEDI

To simulate paired UMI counts (similar to, for example, UMI counts that are observed from analysis of spliced and unspliced RNA), we started with matrix **M** containing UMI counts for 11,532 genes across 17,180 cells from a real scRNA-seq dataset (PBMC dataset[1] donor 1). We used the matrix **M** to create two other matrices of the same dimensions, $\mathbf{M}_1$ and $\mathbf{M}_2$, based on binomial subsampling of each element $m_{g,n}$ of **M** according to the distribution $m_{1,g,n} \sim \mathrm{B}(m_{g,n}, p_{g,n})$, and $m_{2,g,n} = m_{g,n} - m_{1,g,n}$. The sampling probability $p_{g,n}$ for each gene g in each cell n was chosen as $p_{g,n} = 1/\left[1 - \exp\left[-(\mathbf{ZB})_{g,n} + o_g + s_n\right]\right]$, where **Z** and **B** are two matrices of rank 20, with each element sampled from $N(0,0.5)$, and **o** and **s** are vectors representing gene-specific and cell-specific offsets, respectively, sampled from $N(0,1)$. (**a**) Comparison of the ground-truth gene-specific offset **o** with GEDI-inferred gene-specific offsets. Pearson correlation is shown. (**b**) Comparison of the ground-truth cell-specific offset **s** with GEDI-inferred cell-specific offsets. (**c**) Comparison of the ground-truth logit of $p_{g,n}$ to a naïve estimator, obtained as $log[(m_{1,g,n}+1)/(m_{2,g,n}+1)]$. Each data point represents one gene in one cell. (**d**) Comparison of the ground-truth logit of $p_{g,n}$ to the GEDI-imputed values, i.e., the expected value of the latent $y_{g,n}$ given the observed (simulated) $m_{1,g,n}$ and $m_{1,g,n}$ UMI counts and the model-predicted value. (**e**) Comparison of the ground-truth logit of $p_{g,n}$ to the model-predicted values. (**f**) Comparison of the ground-truth logit of $p_{g,n}$ to imputed values from MAGIC[13]. (**g**) Comparison of the ground-truth logit of $p_{g,n}$ to imputed values from SAVER[14].

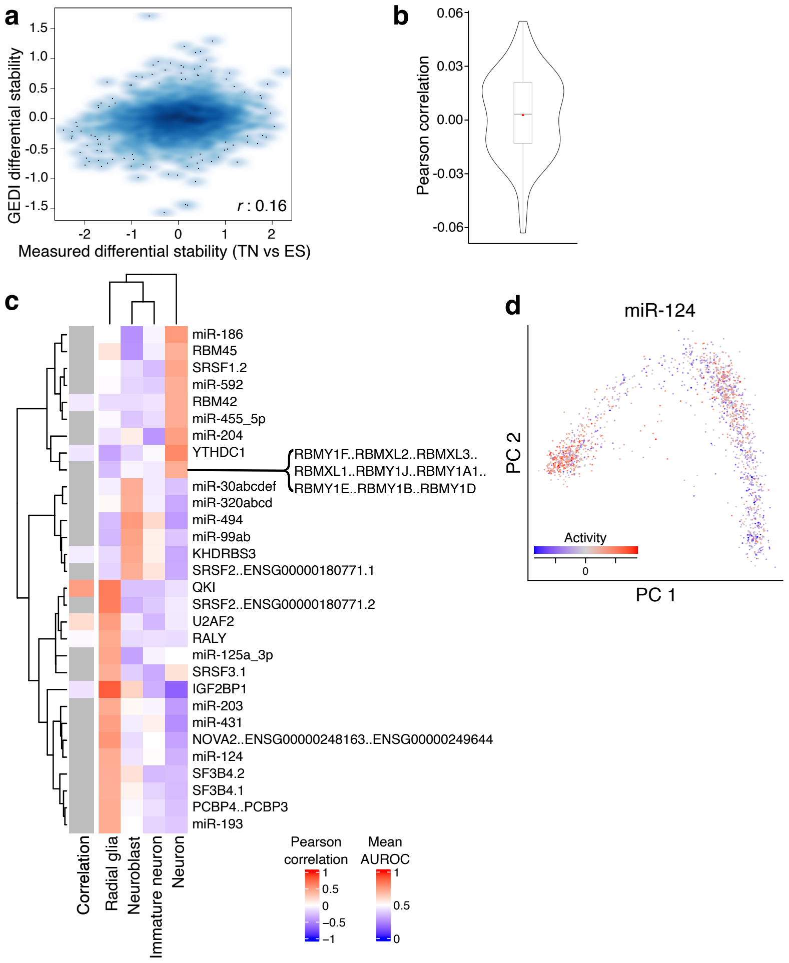

Supplementary Figure 17. Analysis of the latent stability space learned by GEDI

(**a**) Comparison between differential stability estimates inferred from GEDI vs. RNA half-life measurements obtained from mouse embryonic stem cells (ESCs) and in vitro-differentiated terminal neurons (TNs)[15]. GEDI estimates were obtained after analyzing the ratio of spliced and unspliced transcripts at the single-cell level in a model of sensory neurogenesis[16]. Differential stability estimates were obtained by calculating the slope of the imputed log-ratio of spliced vs. unspliced transcripts vs. pseudotime, which represents stability changes per unit of time. Pseudotime scores were obtained from the original publication[16]. Pearson correlation between GEDI estimates and experimental measurements of stability is shown. (**b**) Null distribution of the Pearson correlation values between GEDI stability estimates and experimental measurements of stability. Stability analysis was performed as in (a) but shuffling the pseudo-time labels during the estimation of differential stability with GEDI. Violin plot and boxplot shows the distribution of 100 Pearson correlation values with shuffled pseudo-time labels, red dot represents the mean AUROC. (**c**) GEDI identifies cell type-specific activities of post-transcriptional regulators. GEDI was applied to analyze the ratio of unspliced and spliced RNAs in human neurons, using a previously published dataset of human embryonic glutamatergic neurogenesis[17]. In this analysis, we modeled the spliced/unspliced latent manifold as a function of the regulatory networks of RNA binding proteins (RBPs) and miRNAs. Heatmap shows AUROC values for differential enrichment of inferred post-transcriptional regulator activities from GEDI (rows) for each cell type (columns). Left annotation heatmap shows Pearson correlation between inferred activity and model-fitted mRNA abundance (for RBPs). Gray values indicate not available values of expression of miRNAs. (**d**) PCA of the human embryonic glutamatergic dataset[17]. The color shows the projected regulon activity of miR-124, which is an inhibitor of mRNA stability[18]. Activity pattern shows that the targets of miR-124 are more stable in radial glia and less stable in differentiated neurons, consistent with higher miR-124 activity in differentiated neurons.

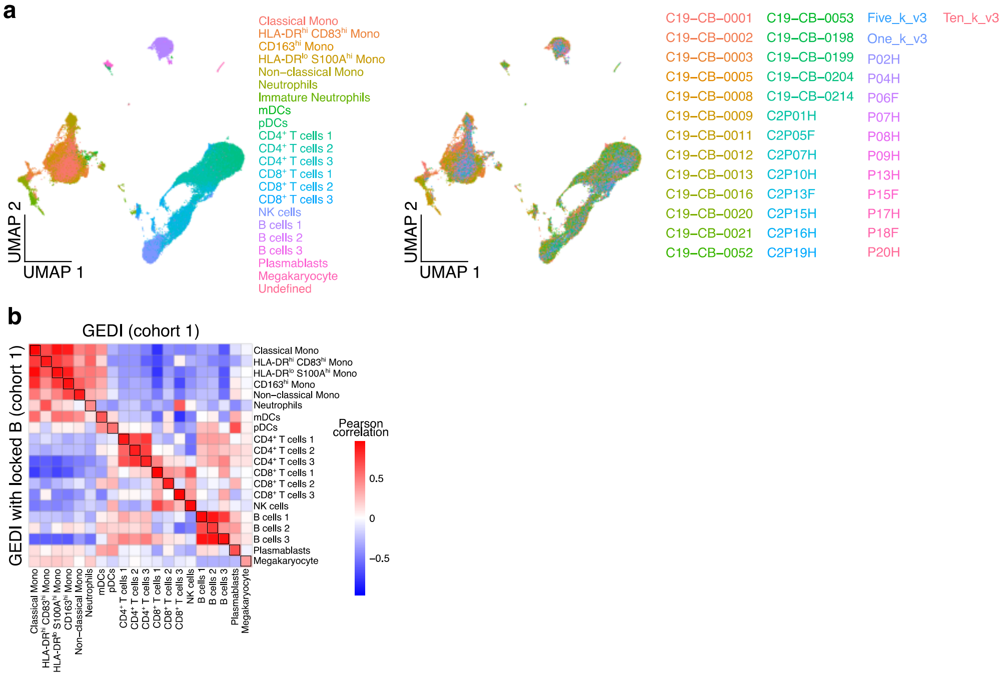

Supplementary Figure 18. Post-hoc analysis of prespecified integrated cell state spaces with GEDI
(**a**) UMAP embedding of the cells in the COVID-19 cohort 1 dataset after integration with Harmony[19]. Each dot represents one cell, colored by the cell type labels from the original study (left) or by sample (right). (**b**) The mean transcriptomic vector field, representing expression shift in mild COVID-19 relative to healthy controls, was obtained using GEDI based on the integrated cell state space from Harmony, or by *de novo* integration of data with GEDI. The heatmap shows the pairwise Pearson correlations of the mean transcriptomic vectors of cell types between the Harmony-based GEDI analysis (rows) and *de novo* GEDI analysis (columns).

Supplementary Table 1. List of source data files
The Zenodo records are available at:
https://zenodo.org/doi/10.5281/zenodo.8222039,
https://zenodo.org/doi/10.5281/zenodo.8222697,
https://zenodo.org/doi/10.5281/zenodo.11163741,
https://zenodo.org/doi/10.5281/zenodo.11164776.

**File name:** pbmc_lis_K_v2.rds (related to Figure 2)<br>**Description:** List with embeddings for the integration of the PBMC dataset. List contains results across various number of latent variables (K) and integration methods. For a given K and method, there are three objects: 'embedding_res': embeddings after integration; 'umap_2_res': UMAP embeddings with two dimensions; 'umap_n_res': UMAP embeddings with *n* dimensions, where *n* is equal to the number of latent variables K.<br>**Download URL:** https://zenodo.org/records/11163742/files/pbmc_lis_K_v2.rds?download=1
**File name:** pancreas_lis_K_v2.rds (related to Figure 2)<br>**Description:** Same as above, but for the Pancreas dataset.<br>**Download URL:** https://zenodo.org/records/11163742/files/pancreas_lis_K_v2.rds?download=1
**File name:** tabulaMuris_lis_K_v2.rds (related to Figure 2)<br>**Description:** Same as above, but for the Tabula Muris dataset.<br>**Download URL:** https://zenodo.org/records/11163742/files/tabulaMuris_lis_K_v2.rds?download=1
**File name:** pbmc_gedi_model_bothDonors.rds (related to Figure 2)<br>**Description:** GEDI object of PBMC data.<br>**Download URL:** https://zenodo.org/record/8222040/files/pbmc_gedi_model_bothDonors.rds?download=1
**File name:** COVID19_gedi_model_bothCohorts.rds (related to Figure 2)<br>**Description:** GEDI object of COVID-19 data (two cohorts).<br>**Download URL:** https://zenodo.org/record/8222040/files/COVID19_gedi_model_bothCohorts.rds?download=1
**File name:** COVID19_gedi_model_cohort1.rds (related to Figure 3)<br>**Description:** GEDI object of COVID-19 data (cohort1), with sample-level variables incorporated in the model.<br>**Download URL:** https://zenodo.org/record/8222040/files/COVID19_gedi_model_cohort1.rds?download=1
**File name:** COVID19_gedi_model_cohort2.rds (related to Figure 3)<br>**Description:** GEDI object for COVID-19 data (cohort2), with sample-level variables incorporated in the model.<br>**Download URL:** https://zenodo.org/record/8222698/files/COVID19_gedi_model_cohort2.rds?download=1
**File name:** COVID19_list_DE.rds (related to Figure 3)<br>**Description:** List with Differential Expression results for DESeq2 and GEDI, for the severe vs control and mild vs control comparisons.<br>**Download URL:** https://zenodo.org/record/8222698/files/COVID19_list_DE.rds?download=1
**File name:** CFDE_simulated_data.rds (related to Figure 4)<br>**Description:** List with objects for the simulated dataset used for cluster-free differential expression analysis. List contains: 'meta': metadata; 'raw_counts': raw counts of the simulated data; 'DE_ground_truth_mild': ground truth differential expression estimates (log2 fold-change) per cell for the comparison of mild vs control; 'DE_ground_truth_severe;: ground truth differential expression estimates (log2 fold-change) per cell for the comparison of severe vs control.<br>**Download URL:** https://zenodo.org/records/11163742/files/CFDE_simulated_data.rds?download=1
**File name:** CFDE_gedi_Bl2.rds (related to Figure 4)<br>**Description:** List with GEDI-hyperplane results of cluster-free differential expression using the simulated data. List contains: 'DE_gedi_bl2_mild': differential expression estimates (log fold-change) per cell for the comparison of mild vs control; 'DE_gedi_bl2_severe': differential expression estimates (log fold-change) per cell for the comparison of severe vs control.<br>**Download URL:** https://zenodo.org/records/11163742/files/CFDE_gedi_Bl2.rds?download=1
**File name:** CFDE_gedi_Bsphere.rds (related to Figure 4)<br>**Description:** Same as above, but for GEDI-hypersphere.<br>**Download URL:** https://zenodo.org/records/11163742/files/CFDE_gedi_Bsphere.rds?download=1
**File name:** CFDE_lemur.rds (related to Figure 4)<br>**Description:** Same as above, but for LEMUR.<br>**Download URL:** https://zenodo.org/records/11164777/files/CFDE_lemur.rds?download=1
**File name:** CFDE_miloDE.rds (related to Figure 4)<br>**Description:** Same as above, but for miloDE. Here, differential expression estimates are at the neighborhood level.

**Download URL:** https://zenodo.org/records/11164777/files/CFDE_miloDE.rds?download=1
**File name:** CFDE_deseq2.rds (related to Figure 4)<br>**Description:** Same as above, but for DESeq2, at the cell type level.<br>**Download URL:** https://zenodo.org/records/11164777/files/CFDE_deseq2.rds?download=1
**File name:** CFDE_performance_stats.rds (related to Figure 4)<br>**Description:** List with statistics comparing cluster-free differential expression methods. List contains pearson correlation, AUROC for up-regulated genes and AUROC for down-regulated genes values at the single-cell, neighborhood and cell type levels, for the severe vs control and mild vs control comparisons.<br>**Download URL:** https://zenodo.org/records/11164777/files/CFDE_performance_stats.rds?download=1
**File name:** pbmc_gedi_model_donor1_celltype.rds (related to Figure 5)<br>**Description:** GEDI object of PBMC data (donor 1), with prior information of cell type signatures incorporated in the model.<br>**Download URL:** https://zenodo.org/record/8222698/files/pbmc_gedi_model_donor1_celltype.rds?download=1
**File name:** pbmc_gedi_model_donor2_celltype.rds (related to Figure 5)<br>**Description:** GEDI object of PBMC data (donor 2), with prior information of cell type signatures incorporated in the model.<br>**Download URL:** https://zenodo.org/record/8222698/files/pbmc_gedi_model_donor2_celltype.rds?download=1
**File name:** pbmc_gedi_model_bothDonors_TF.rds (related to Figure 5)<br>**Description:** GEDI object of PBMC data, with prior information of transcription factor regulatory networks incorporated in the model.<br>**Download URL:** https://zenodo.org/record/8222698/files/pbmc_gedi_model_bothDonors_TF.rds?download=1
**File name:** COVID19_gedi_model_cohort1_TF.rds (related to Figure 5<br>**Description:** GEDI object of COVID-19 data (cohort1), with prior information of transcription factor regulatory networks and sample-level variables both incorporated in the model.<br>**Download URL:** https://zenodo.org/record/8222698/files/COVID19_gedi_model_cohort1_TF.rds?download=1
**File name:** Tasic_gedi_model.rds (related to Figure 6)<br>**Description:** GEDI object of Tasic dataset.<br>**Download URL:** https://zenodo.org/record/8222698/files/Tasic_gedi_model.rds?download=1
**File name:** Faure_gedi_model.rds (related to Figure 6)<br>**Description:** GEDI object of Faure dataset.<br>**Download URL:** https://zenodo.org/record/8222698/files/Faure_gedi_model.rds?download=1
**File name:** LaManno_gedi_model.rds (related to Figure 6)<br>**Description:** GEDI object of La Manno data, with prior information of post-transcriptional regulatory networks incorporated in the model.<br>**Download URL:** https://zenodo.org/records/11165452/files/LaManno_gedi_model_v2.rds?download=1

Supplementary Methods

1 The GEDI framework

1.1 Fitting the GEDI model with no prior gene-level or sample-level information

We start by discussing the solution for the simplest GEDI model, i.e., a model in which measurement matrix $\mathbf{Y}\in\mathbb{R}^{G\times N}$ is directly provided, there is no prior gene-level information, and there is no prior sample-level information. In this case, we aim to obtain the maximum a posteriori (MAP) estimate of the parameter set Θ:

$$\Theta = \{\mathbf{o}_r, \mathbf{Z}_r, \Delta\mathbf{o}_1, \ldots, \Delta\mathbf{o}_Q, \Delta\mathbf{Z}_1, \ldots, \Delta\mathbf{Z}_Q, \mathbf{B}, \mathbf{s}, \sigma^2\}$$

$$\widehat{\Theta}_{\text{MAP}}(\mathbf{Y}) = \arg\max_\Theta f(\mathbf{Y}|\Theta)g(\Theta)$$

Here, Q represents the total number of datasets that are being integrated, f is the sampling distribution of $\mathbf{Y}$ conditional on Θ, and g is the prior distribution of Θ. Other parameters are the same as defined in the main **Methods** text.

$\mathbf{Y}$ consists of N column vectors $\mathbf{y}_{n\in\{1,\ldots,N\}}$; each vector $\mathbf{y}_n$ represents the measurements of G genes/events in cell n. We assume that each observation $\mathbf{y}_n$ is independent of other observations conditional on Θ. Therefore:

$$\mathbf{y}_n|\Theta \sim \mathcal{N}\left(\mathbf{o}_r + \Delta\mathbf{o}_{i(n)} + \left(\mathbf{Z}_r + \Delta\mathbf{Z}_{i(n)}\right)\mathbf{b}_n + s_n\mathbf{1}_G, \sigma^2\mathbf{I}\right) \tag{1}$$

The column vector $\mathbf{o}_r\in\mathbb{R}^G$ represents the origin point on a reference hyperplane, with each element $o_{r,g}$ ($g\in\{1,\ldots,G\}$) derived from the following prior:

$$o_{r,g} \sim \mathcal{N}(\mu_o, \sigma^2 S_o\mathbf{I})$$

$\Delta\mathbf{o}_i$ represents the sample-specific translation of the origin point. These vectors have the following priors:

$$\Delta\mathbf{o}_i \sim \mathcal{N}\left(\mathbf{0}, \sigma^2 S_{\Delta o_i}\mathbf{I}\right)$$

Each column $\mathbf{z}_{r,k}$ of the matrix $\mathbf{Z}_r\in\mathbb{R}^{G\times K}$ represents a vector that originates from point $\mathbf{o}_r$ and lies on the reference hyperplane, and has the following prior:

$$\mathbf{z}_{r,k} \sim \mathcal{N}(\mathbf{0}, \sigma^2 S_Z\mathbf{I})$$

Each column $\Delta\mathbf{z}_{i,k}$ of each matrix $\Delta\mathbf{Z}_i\in\mathbb{R}^{G\times K}$ (for all $i\in\{1,\ldots,Q\}$) represents the sample-specific distortion of $\mathbf{z}_{r,k}$, and has the following prior:

$$\Delta\mathbf{z}_{i,k} \sim \mathcal{N}\left(\mathbf{0}, \sigma^2 S_{\Delta Z_i}\mathbf{I}\right)$$

Finally, each s_n ($n\in\{1,\ldots,N\}$) is a cell-specific intercept (representing library size) with the following prior:

$$s_n \sim N\left(\mu_{s,n}, \sigma_s^2\right)$$

The above priors include hyperparameters μ_o, S_o, $S_{\Delta oi}$, S_Z, $S_{\Delta Zi}$, $\mu_{s,n}$ and σ_s^2. The choice of these hyperparameters are discussed in **section 1.6**.

The MAP estimate of the parameter set can be obtained as follows:

$$\begin{aligned}
\widehat{\Theta}_{\text{MAP}}(\mathbf{Y}) &= \arg\max_\Theta f(\mathbf{Y}|\Theta)g(\Theta) = \arg\min_\Theta[-\log[f(\mathbf{Y}|\Theta)g(\Theta)]] \\
&= \arg\min_\Theta \Bigg[\frac{GN}{2}\log(2\pi\sigma^2) + \frac{1}{2\sigma^2}\sum_{n\in\{1,\ldots,N\}}\left\|\mathbf{y}_n - \mathbf{o}_r - \Delta\mathbf{o}_{i(n)} - \left(\mathbf{Z}_r + \Delta\mathbf{Z}_{i(n)}\right)\mathbf{b}_n - s_n\mathbf{1}_G\right\|_2^2 \\
&\quad + \frac{G}{2}\log(2\pi\sigma^2 S_o) + \frac{1}{2\sigma^2 S_o}\|\mathbf{o}_r - \mu_o\mathbf{1}_G\|_2^2 + \sum_{i\in\{1,\ldots,Q\}}\left[\frac{G}{2}\log\left(2\pi\sigma^2 S_{\Delta o_i}\right) + \frac{1}{2\sigma^2 S_{\Delta o_i}}\|\Delta\mathbf{o}_i\|_2^2\right] \\
&\quad + \frac{GK}{2}\log(2\pi\sigma^2 S_Z) + \frac{1}{2\sigma^2 S_Z}\|\mathbf{Z}_r\|_F^2 + \sum_{i\in\{1,\ldots,Q\}}\left[\frac{GK}{2}\log\left(2\pi\sigma^2 S_{\Delta z_i}\right) + \frac{1}{2\sigma^2 S_{\Delta z_i}}\|\Delta\mathbf{Z}_i\|_F^2\right] \\
&\quad + \frac{1}{2\sigma_s^2}\sum_{n\in\{1,\ldots,N\}}\left(s_n - \mu_{s,n}\right)^2\Bigg]
\end{aligned} \tag{2}$$

We use block coordinate descent to iteratively optimize the parameters until convergence, as described below.

1.1.1 Solving $\mathbf{Z}_r$

From Eq. (2), if we isolate the terms that contain $\mathbf{Z}_r$, we have:

$$\widehat{\mathbf{Z}_r} = \arg\min_{\mathbf{Z}_r}\left[\frac{1}{2\sigma^2}\sum_{n\in\{1,\dots,N\}}\left\|\mathbf{y}_n - \mathbf{o}_r - \Delta\mathbf{o}_{i(n)} - \left(\mathbf{Z}_r + \Delta\mathbf{Z}_{i(n)}\right)\mathbf{b}_n - s_n\mathbf{1}_G\right\|_2^2 + \frac{1}{2\sigma^2 S_Z}\|\mathbf{Z}_r\|_F^2\right]$$
$$= \arg\min_{\mathbf{Z}_r}\left[\sum_{n\in\{1,\dots,N\}}\left\|\mathbf{y}_n - \mathbf{o}_r - \Delta\mathbf{o}_{i(n)} - \left(\mathbf{Z}_r + \Delta\mathbf{Z}_{i(n)}\right)\mathbf{b}_n - s_n\mathbf{1}_G\right\|_2^2 + \frac{1}{S_Z}\|\mathbf{Z}_r\|_F^2\right]$$

When $\mathbf{Z}_r$ is not restricted to be orthogonal, we can simply solve it as:

$$\widehat{\mathbf{Z}_r} = \mathbf{Y}'\mathbf{B}^T\left(\mathbf{B}\mathbf{B}^T + \frac{1}{S_Z}\mathbf{I}\right)^{-1}$$

where

$$\mathbf{Y}' = [\mathbf{y}'_1 \quad \dots \quad \mathbf{y}'_N]$$
$$\mathbf{y}'_n = \mathbf{y}_n - \mathbf{o}_r - \Delta\mathbf{o}_{i(n)} - \Delta\mathbf{Z}_{i(n)}\mathbf{b}_n - s_n\mathbf{1}_G$$

(3)

When $\mathbf{Z}_r$ is restricted so that its columns are orthogonal to each other, we solve the problem by first defining $\mathbf{Z}_r$ as the product of two matrices $\mathbf{U}\in\mathbb{R}^{G\times K}$ and $\mathbf{S}\in\mathbb{R}^{K\times K}$, where $\mathbf{U}$ is a matrix with orthonormal columns and $\mathbf{S}=\text{diag}(\boldsymbol{s})$ is a diagonal matrix. This leads to the following minimization problem:

$$\widehat{\mathbf{Z}_r} = \widehat{\mathbf{U}}\widehat{\mathbf{S}}$$
$$\left\{\widehat{\mathbf{U}},\widehat{\mathbf{S}}\right\} = \arg\min_{\{\mathbf{U},\mathbf{S}\}}\left\{\|\mathbf{Y}' - \mathbf{USB}\|_F^2 + \frac{1}{S_Z}\|\mathbf{US}\|_F^2\right\} = \arg\min_{\{\mathbf{U},\mathbf{S}\}}\|\mathbf{Y}'' - \mathbf{USB}'\|_F^2$$

where:

$$\mathbf{Y}'' = [\mathbf{Y}' \quad \mathbf{0}_{G\times K}]$$
$$\mathbf{B}' = \left[\mathbf{B} \quad \frac{1}{\sqrt{S_Z}}\mathbf{I}_{K\times K}\right]$$

We solve $\mathbf{U}$ and $\mathbf{S}$ iteratively as part of the block coordinate descent algorithm. First, to solve $\mathbf{S}$, we want to obtain:

$$\widehat{\mathbf{S}} = \arg\min_{\mathbf{S}}\|\mathbf{Y}'' - \mathbf{USB}'\|_2^2 = \arg\min_{\mathbf{S}}\sum_{g=1}^{G}\sum_{n=1}^{N+K}\left(y''_{g,n} - \sum_{k=1}^{K}u_{g,k}s_k b'_{k,n}\right)^2$$
$$= \arg\min_{\mathbf{S}}\sum_g\sum_n\left(y''_{g,n}\right)^2 - 2\sum_g\sum_n\left(y''_{g,n}\sum_k u_{g,k}s_k b'_{k,n}\right) + \sum_g\sum_n\left(\sum_k u_{g,k}s_k b'_{k,n}\right)^2$$

For each element s_κ, we can separately solve by setting its derivative to zero:

$$\frac{d}{d(s_\kappa)}\left[\sum_g\sum_n\left(y''_{g,n}\right)^2 - 2\sum_g\sum_n\left(y''_{g,n}\sum_k u_{g,k}s_k b'_{k,n}\right) + \sum_g\sum_n\left(\sum_k u_{g,k}s_k b'_{k,n}\right)^2\right] = 0$$
$$\Rightarrow -2\sum_g\sum_n\left(y''_{g,n}u_{g,\kappa}b'_{\kappa,n}\right) + 2\sum_g\sum_n u_{g,\kappa}b'_{\kappa,n}\sum_k u_{g,k}s_k b'_{k,n} = 0$$
$$\Rightarrow -\sum_g\sum_n\left(y''_{g,n}u_{g,\kappa}b'_{\kappa,n}\right) + s_\kappa\sum_g\sum_n\left(u_{g,\kappa}b'_{\kappa,n}\right)^2 + \sum_g\sum_n u_{g,\kappa}b'_{\kappa,n}\sum_{k\neq\kappa}u_{g,k}s_k b'_{k,n} = 0$$

which can be rearranged as:

$$s_\kappa\sum_g\sum_n\left(u_{g,\kappa}b_{\kappa,n}\right)^2 = \sum_g\sum_n\left(y''_{g,n}u_{j,\kappa}b'_{\kappa,n}\right) - \sum_g\sum_n u_{g,\kappa}b'_{\kappa,n}\sum_{k\neq\kappa}u_{g,k}s_k b'_{k,n}$$
$$\Rightarrow s_\kappa = \frac{\sum_g\sum_n\left(y''_{g,n}u_{g,\kappa}b'_{\kappa,n}\right) - \sum_g\sum_n u_{g,\kappa}b'_{\kappa,n}\sum_{k\neq\kappa}u_{g,k}s_k b'_{k,n}}{\sum_g\sum_n\left(u_{g,\kappa}b'_{\kappa,n}\right)^2}$$
$$= \frac{\sum_g\sum_n\left(y''_{g,n}u_{g,\kappa}b'_{\kappa,n}\right) - \sum_n\sum_{k\neq\kappa}b'_{\kappa,n}s_k b'_{k,n}\sum_g u_{g,\kappa}u_{g,k}}{\sum_g\sum_n\left(u_{g,\kappa}b'_{\kappa,n}\right)^2}$$

Then, considering the fact that the different columns of $\mathbf{U}$ are orthonormal, and therefore their dot products are zero, this equation simplifies to:

$$s_\kappa = \frac{\sum_g \sum_n \left(y''_{g,n} u_{g,\kappa} b'_{\kappa,n}\right)}{\sum_g \sum_n \left(u_{g,\kappa} b'_{\kappa,n}\right)^2} = \frac{\sum_g u_{g,\kappa} \sum_n b'_{\kappa,n} y''_{g,n}}{\sum_n \left(b'_{\kappa,n}\right)^2 \sum_g \left(u_{g,\kappa}\right)^2} = \frac{\sum_g u_{g,\kappa} \sum_n b'_{\kappa,n} y''_{g,n}}{\sum_n \left(b'_{\kappa,n}\right)^2} = \frac{\sum_g u_{g,\kappa} \sum_n b'_{\kappa,n} y''_{g,n}}{1 + \frac{1}{S_Z}}$$

$$= \frac{\sum_j u_{j,\kappa} [\mathbf{Y}'(\mathbf{B}')^T]_{j,\kappa}}{1 + \frac{1}{S_Z}} \tag{4}$$

To solve **U**, we have:

$$\widehat{\mathbf{U}} = \arg\min_{\mathbf{S}} \|\mathbf{Y}'' - \mathbf{USB}'\|_2^2$$

When **S** is given, this problem transforms into a Procrustes problem. Therefore, we can solve **U** by performing SVD on $\mathbf{Y}''(\mathbf{SB}')^T$:

$$\mathrm{SVD}[\mathbf{Y}''(\mathbf{SB}')^T] = \mathbf{U}'\mathbf{S}'\mathbf{V}^{\boldsymbol{T}}$$
$$\mathbf{U} = \mathbf{U}'\mathbf{V}^{\boldsymbol{T}} \tag{5}$$

1.1.2 Solving $\Delta\mathbf{Z}_i$

For each given $i \in \{1,\ldots,Q\}$, we can isolate from Eq. (2) the terms that contain $\Delta\mathbf{Z}_i$:

$$\widehat{\Delta\mathbf{Z}_\iota} = \arg\min_{\Delta\mathbf{Z}_i} \left[\frac{1}{2\sigma^2} \sum_{n \in \mathcal{D}_i} \|\mathbf{y}_n - \mathbf{o}_r - \Delta\mathbf{o}_i - (\mathbf{Z}_r + \Delta\mathbf{Z}_i)\mathbf{b}_n - s_n \mathbf{1}_G\|_2^2 + \frac{1}{2\sigma^2 S_{\Delta z_i}} \|\Delta\mathbf{Z}_i\|_F^2\right]$$

Here, $\mathcal{D}_i$ is the set of indices for all cells that belong to sample (dataset) i, i.e., $\mathcal{D}_i \in \{n | i(n) = i\}$, where $i(n)$ is the sample to which cell n belongs. We can solve $\Delta\mathbf{Z}_i$ as:

$$\widehat{\Delta\mathbf{Z}_\iota} = \mathbf{Y}'_i \mathbf{B}_i^T \left(\mathbf{B}_i \mathbf{B}_i^T + \frac{1}{S_{\Delta z_i}} \mathbf{I}\right)^{-1} \tag{6}$$

Here, $\mathbf{B}_i$ is a submatrix containing only columns $\mathcal{D}_i$ of of **B** (i.e, the concatenation of $\mathbf{b}_n$ column vectors for all cells n that belong to sample i). Similarly, $\mathbf{Y}'_i$ here is defined as the concatenation of column vectors $\mathbf{y}'_n$ for all cells n that belong to sample i, with $\mathbf{y}'_n$ here defined as:

$$\mathbf{y}'_n = \mathbf{y}_n - \mathbf{o}_r - \Delta\mathbf{o}_{i(n)} - \mathbf{Z}_r \mathbf{b}_n - s_n \mathbf{1}_G$$

1.1.3 Solving $\mathbf{o}_r$

Following the same procedure as the sections above, we have:

$$\widehat{\mathbf{o}_r} = \arg\min_{\mathbf{o}_r} \left[\frac{1}{2\sigma^2} \sum_{n \in \{1,\ldots,N\}} \left\|\mathbf{y}_n - \mathbf{o}_r - \Delta\mathbf{o}_{i(n)} - \left(\mathbf{Z}_r + \Delta\mathbf{Z}_{i(n)}\right)\mathbf{b}_n - s_n \mathbf{1}_G\right\|_2^2 + \frac{1}{2\sigma^2 S_o} \|\mathbf{o}_r - \mu_o \mathbf{1}_G\|_2^2\right]$$

$$\widehat{\mathbf{o}_r} = \frac{\mathbf{Y}'\mathbf{1}_N + \frac{1}{S_o}\mu_o \mathbf{1}_G}{N + \frac{1}{S_o}} \tag{7}$$

Here, $\mathbf{Y}'$ is defined as:

$$\mathbf{Y}' = [\mathbf{y}'_1 \quad \ldots \quad \mathbf{y}'_N]$$
$$\mathbf{y}'_n = \mathbf{y}_n - \Delta\mathbf{o}_{i(n)} - \left(\mathbf{Z}_r + \Delta\mathbf{Z}_{i(n)}\right)\mathbf{b}_n - s_n \mathbf{1}_G$$

1.1.4 Solving $\Delta\mathbf{o}_i$

Similar to the previous sections, we can isolate the terms that contain Δo, which leads to the following minimization problem:

$$\widehat{\Delta\mathbf{o}_\iota} = \arg\min_{\Delta o_i} \left[\frac{1}{2\sigma^2} \sum_{n \in \mathcal{D}_i} \|\mathbf{y}_n - \mathbf{o}_r - \Delta\mathbf{o}_i - (\mathbf{Z}_r + \Delta\mathbf{Z}_i)\mathbf{b}_n - s_n \mathbf{1}_G\|_2^2 + \frac{1}{2\sigma^2 S_{\Delta o_i}} \|\Delta\mathbf{o}_i\|_2^2\right]$$

$$\widehat{\mathbf{o}_r} = \frac{\mathbf{Y}'_i \mathbf{1}_{N_i}}{N_i + \frac{1}{S_{\Delta o_i}}} \tag{8}$$

Here, N_i is the number of cells in sample i, i.e., $N_i=|\mathcal{D}_i|$, and $\mathbf{Y}'_i$ here is defined as the concatenatation of column vectors $\mathbf{y}'_n$ for all cells $n\in\mathcal{D}_i$, with $\mathbf{y}'_n$ here defined as:

$$\mathbf{y}'_n = \mathbf{y}_n - \mathbf{o}_r - \left(\mathbf{Z}_r + \Delta\mathbf{Z}_{i(n)}\right)\mathbf{b}_n - s_n \mathbf{1}_G$$

1.1.5 Solving B

By isolating the terms containing **B** in Eq. (2), we have:

$$\widehat{\mathbf{B}} = \arg\min_{\mathbf{B}\in\mathcal{B}} \frac{1}{2\sigma^2} \sum_{n\in\{1,\ldots,N\}} \left\|\mathbf{y}_n - \mathbf{o}_r - \Delta\mathbf{o}_{i(n)} - \left(\mathbf{Z}_r + \Delta\mathbf{Z}_{i(n)}\right)\mathbf{b}_n - s_n \mathbf{1}_G\right\|_2^2$$

Here, $\mathcal{B}\subseteq\mathbb{R}^{K\times N}$ is the subset of $\mathbb{R}^{K\times N}$ to which **B** is restricted. In its simplest form, we have:

$$\mathcal{B} = \left\{\mathbb{R}^{K\times N}\middle|\ \forall k \in \{1,\ldots,K\}\ \textstyle\sum_{n=1}^{N}\left(b_{k,n}\right)^2 = 1\right\}$$

We can solve separately for each $\mathbf{b}_n$:

$$\widehat{\mathbf{b}_n} = \arg\min_{\mathbf{b}_n} \left\|\mathbf{y}_n - \mathbf{o}_r - \Delta\mathbf{o}_{i(n)} - \left(\mathbf{Z}_r + \Delta\mathbf{Z}_{i(n)}\right)\mathbf{b}_n - s_n \mathbf{1}_G\right\|_2^2$$

This leads us to:

$$\widehat{\mathbf{b}'_n} = \left(\mathbf{W}_{i(n)}^T \mathbf{W}_{i(n)}\right)^{-1} \mathbf{W}_{i(n)}^T \mathbf{y}'_n$$
$$\widehat{\mathbf{b}_n} = \widehat{\mathbf{b}'_n} \odot \mathbf{d} \tag{9}$$

Here, $\mathbf{W}_{i(n)}$ is defined as $\mathbf{W}_{i(n)}=\mathbf{Z}_r+\Delta\mathbf{Z}_{i(n)}$, and $\mathbf{y}'_n$ is defined as $\mathbf{y}'_n=\mathbf{y}_n-\mathbf{o}_r-\Delta\mathbf{o}_{i(n)}-s_n\mathbf{1}_G$. The operator $\odot$ is the Hadamard product, and **d** is a vector of normalization factors, so that each row of **B** becomes a unit vector.

With the added restriction that ***B*** should be restricted to the points on an ellipsoid, the above equation can be modified as follows:

$$\widehat{\mathbf{b}'_n} = \rho_n \left(\mathbf{W}_{i(n)}^T \mathbf{W}_{i(n)}\right)^{-1} \mathbf{W}_{i(n)}^T \mathbf{y}'_n$$
$$\widehat{\mathbf{b}_n} = \widehat{\mathbf{b}'_n} \odot \mathbf{d} \tag{10}$$

Here, ρ_n is a cell-specific normalization factor so that each $\mathbf{b}'_n$ becomes a unit vector (and, therefore, all points $\mathbf{b}'_n$ lie on the surface of a unit (K–1)-sphere). As in above, **d** is a vector of normalization factors, so that each row of **B** becomes a unit vector, but it also represents the lengths of the semi-axes of the ellipsoid on which the solution for **B** lies.

1.1.6 Solving σ^2

Let's start by defining the following sum:

$$\mathcal{S} = \sum_{n\in\{1,\ldots,N\}} \left\|\mathbf{y}_n - \mathbf{o}_r - \Delta\mathbf{o}_{i(n)} - \left(\mathbf{Z}_r + \Delta\mathbf{Z}_{i(n)}\right)\mathbf{b}_n - s_n \mathbf{1}_G\right\|_2^2 + \frac{1}{S_o}\|\mathbf{o}_r - \mu_o \mathbf{1}_G\|_2^2 + \sum_{i\in\{1,\ldots,Q\}} \frac{1}{S_{\Delta o_i}}\|\Delta\mathbf{o}_i\|_2^2 + \frac{1}{S_Z}\|\mathbf{Z}_r\|_F^2 + \sum_{i\in\{1,\ldots,Q\}} \frac{1}{S_{\Delta z_i}}\|\Delta\mathbf{Z}_i\|_F^2$$

We can then rewrite Eq. (2) as:

$$\widehat{\sigma^2} = \arg\min_{\sigma^2}\left[\frac{GN}{2}\log(2\pi\sigma^2) + \frac{G}{2}\log(2\pi\sigma^2 S_o) + \frac{G}{2}\sum_{i\in\{1,\ldots,Q\}}\log\left(2\pi\sigma^2 S_{\Delta o_i}\right) + \frac{GK}{2}\log(2\pi\sigma^2 S_Z) + \frac{GK}{2}\sum_{i\in\{1,\ldots,Q\}}\log\left(2\pi\sigma^2 S_{\Delta z_i}\right) + \frac{1}{2\sigma^2}\mathcal{S}\right]$$

We can solve σ^2 by setting its derivative to zero:

$$\frac{d}{d\sigma^2}\left[\frac{GN}{2}\log(2\pi) + \frac{G}{2}\log(2\pi\sigma^2 S_o) + \frac{G}{2}\sum_{i\in\{1,\ldots,Q\}}\log\left(2\pi\sigma^2 S_{\Delta o_i}\right) + \frac{GK}{2}\log(2\pi\sigma^2 S_Z) + \frac{GK}{2}\sum_{i\in\{1,\ldots,Q\}}\log\left(2\pi\sigma^2 S_{\Delta z_i}\right) + \frac{1}{2\sigma^2}\mathcal{S}\right] = 0$$

$$\Rightarrow (GN + G + GQ + GK + GKQ)\frac{1}{\sigma^2} - \left(\frac{1}{\sigma^2}\right)^2 \mathcal{S} = 0$$
$$\Rightarrow \widehat{\sigma^2} = \frac{\mathcal{S}}{GN + G + GQ + GK + GKQ}$$
(11)

1.2 Fitting the GEDI model with gene-level prior information

We can expresses $\mathbf{Z}_r$ as a probabilistic function of $\mathbf{C} \in \mathbb{R}^{G \times P}$, where $\mathbf{C}$ is a matrix representing gene-level prior information matrix. In this case, each column $\mathbf{z}_{r,k}$ of $\mathbf{Z}_r$ is treated as a latent variable with the following conditional probability function:

$$\mathbf{z}_{r,k} | \mathbf{a}_k \sim \mathcal{N}(\mathbf{C}\mathbf{a}_k, \sigma^2 S_Z \mathbf{I})$$
$$\mathbf{a}_k \sim \mathcal{N}(\mathbf{0}, \sigma^2 S_A \mathbf{I})$$

The column vectors $\mathbf{a}_{k \in \{1,\ldots,K\}}$ together form the matrix $\mathbf{A} \in \mathbb{R}^{P \times K}$. Therefore, the parameter set to be optimized will now include:

$$\Theta = \{\mathbf{o}_r, \mathbf{A}, \Delta\mathbf{o}_1, \ldots, \Delta\mathbf{o}_Q, \Delta\mathbf{Z}_1, \ldots, \Delta\mathbf{Z}_Q, \mathbf{B}, \sigma^2\}$$

We need to then optimize the following function:

$$\widehat{\Theta}_{\mathrm{MAP}}(\mathbf{Y}) = \arg\max_\Theta f(\mathbf{Y}|\Theta) g(\Theta) = \arg\max_\Theta \int f(\mathbf{Y}|\Theta, \mathbf{Z}_r) f(\mathbf{Z}_r|\Theta) d\mathbf{Z}_r \, g(\Theta)$$

For simplicity, in what follows, we drop the subscript r from $\mathbf{Z}_r$, and refer to it simply as $\mathbf{Z}$.

We can solve this optimization problem using expectation-maximization. First, at each iteration t, we define the expectation function as:

$$Q(\Theta|\Theta^{(t)}) = E_{\mathbf{Z}|\mathbf{Y},\Theta^{(t)}}[\log[f(\mathbf{Y}|\Theta,\mathbf{Z}) f(\mathbf{Z}|\Theta) g(\Theta)]] = E_{\mathbf{Z}|\mathbf{Y},\Theta^{(t)}}[\log f(\mathbf{Y}|\Theta,\mathbf{Z}) + \log f(\mathbf{Z}|\Theta) + \log g(\Theta)]$$
$$= E_{\mathbf{Z}|\mathbf{Y},\Theta^{(t)}}[\log f(\mathbf{Y}|\Theta,\mathbf{Z}) + \log f(\mathbf{Z}|\Theta)] + \log g(\Theta)$$
$$= E_{\mathbf{Z}|\mathbf{Y},\Theta^{(t)}}[\log f(\mathbf{Y}|\Theta,\mathbf{Z})] + E_{\mathbf{Z}|\mathbf{Y},\Theta^{(t)}}[\log f(\mathbf{Z}|\Theta)] + \log g(\Theta)$$

Then, we maximize the function Q with respect to each parameter using block coordinate descent, as described below.

1.2.1 Solving A

We want to maximize Q with respect to $\mathbf{A}$:

$$\widehat{\mathbf{A}} = \arg\max_\mathbf{A} Q(\Theta|\Theta^{(t)}) = \arg\max_\mathbf{A} \left[E_{\mathbf{Z}|\mathbf{Y},\Theta^{(t)}}[\log f(\mathbf{Y}|\Theta,\mathbf{Z})] + E_{\mathbf{Z}|\mathbf{Y},\Theta^{(t)}}[\log f(\mathbf{Z}|\Theta)] + \log g(\Theta)\right]$$

Note that, conditional on $\mathbf{Z}$, $\mathbf{Y}$ is independent of $\mathbf{A}$. Therefore:

$$\widehat{\mathbf{A}} = \arg\max_\mathbf{A} \left[E_{\mathbf{Z}|\mathbf{Y},\Theta^{(t)}}[\log f(\mathbf{Y}|\Theta,\mathbf{Z})] + E_{\mathbf{Z}|\mathbf{Y},\Theta^{(t)}}[\log f(\mathbf{Z}|\Theta)] + \log g(\Theta)\right]$$
$$= \arg\max_\mathbf{A} \left[E_{\mathbf{Z}|\mathbf{Y},\Theta^{(t)}}[\log f(\mathbf{Z}|\Theta)] + \log g(\Theta)\right] = \arg\min_\mathbf{A} \left[E_{\mathbf{Z}|\mathbf{Y},\Theta^{(t)}}[-\log f(\mathbf{Z}|\Theta)] - \log g(\Theta)\right]$$
$$= \arg\min_\mathbf{A} \left[E_{\mathbf{Z}|\mathbf{Y},\Theta^{(t)}}\left[\frac{1}{2\sigma^2 S_Z}\|\mathbf{Z} - \mathbf{C}\mathbf{A}\|_F^2\right] + \frac{1}{2\sigma^2 S_A}\|\mathbf{A}\|_F^2\right]$$
$$= \arg\min_\mathbf{A} \left[\frac{1}{S_Z} E_{\mathbf{Z}|\mathbf{Y},\Theta^{(t)}}\left[\sum_{g=1}^{G}\sum_{k=1}^{K}\left(z_{g,k} - (\mathbf{C}\mathbf{A})_{g,k}\right)^2\right] + \frac{1}{S_A}\|\mathbf{A}\|_F^2\right]$$

This equation can be expanded as:

$$\widehat{\mathbf{A}} = \arg\min_\mathbf{A} \left[\frac{1}{S_Z}\sum_{g=1}^{G}\sum_{k=1}^{K}\left(E_{\mathbf{Z}|\mathbf{Y},\Theta^{(t)}}(z_{g,k}^2) - 2(\mathbf{C}\mathbf{A})_{g,k} E_{\mathbf{Z}|\mathbf{Y},\Theta^{(t)}}(z_{g,k}) + (\mathbf{C}\mathbf{A})_{g,k}^2\right) + \frac{1}{S_A}\|\mathbf{A}\|_F^2\right]$$

Since $\mathrm{E}(X^2)=\mathrm{Var}(X)+[\mathrm{E}(X)]^2$, we can rewrite the above equation as:

$$\widehat{\mathbf{A}} = \arg\min_{\mathbf{A}} \left[\frac{1}{S_Z} \sum_{g=1}^{G} \sum_{k=1}^{K} \left[\mathrm{Var}_{\mathbf{Z}|\mathbf{Y},\Theta^{(t)}}(z_{g,k}) + \left[E_{\mathbf{Z}|\mathbf{Y},\Theta^{(t)}}(z_{g,k}) \right]^2 - 2(\mathbf{CA})_{g,k} E_{\mathbf{Z}|\mathbf{Y},\Theta^{(t)}}(z_{g,k}) + (\mathbf{CA})_{g,k}^2 \right] + \frac{1}{S_A} \|\mathbf{A}\|_F^2 \right]$$

$$= \arg\min_{\mathbf{A}} \left[\frac{1}{S_Z} \sum_{g=1}^{G} \sum_{k=1}^{K} \mathrm{Var}_{\mathbf{Z}|\mathbf{Y},\Theta^{(t)}}(z_{g,k}) + \frac{1}{S_Z} \sum_{g=1}^{G} \sum_{k=1}^{K} \left(E_{\mathbf{Z}|\mathbf{Y},\Theta^{(t)}}(z_{g,k}) - (\mathbf{CA})_{g,k} \right)^2 + \frac{1}{S_A} \|\mathbf{A}\|_F^2 \right]$$

We note that the variance of each element of **Z**, conditional on **Y** and the current estimates of the parameters $\Theta^{(t)}$, is independent of **A**. Therefore:

$$\widehat{\mathbf{A}} = \arg\min_{\mathbf{A}} \left[\frac{1}{S_Z} \sum_{g=1}^{G} \sum_{k=1}^{K} \left(E_{\mathbf{Z}|\mathbf{Y},\Theta^{(t)}}(z_{g,k}) - (\mathbf{CA})_{g,k} \right)^2 + \frac{1}{S_A} \|\mathbf{A}\|_F^2 \right] = \left(\mathbf{C}^T\mathbf{C} + \frac{S_Z}{S_A} \mathbf{I} \right)^{-1} \mathbf{C}^T E_{\mathbf{Z}|\mathbf{Y},\Theta^{(t)}}(\mathbf{Z}) \tag{12}$$

Solving this equation requires the calculation of the expectation (mean) of **Z** given **Y** and the current estimates of the parameters $\Theta^{(t)}$. When **Z** is not restricted by orthogonality constraint, this is simply a Bayesian linear regression problem, leading to the following expectation:

$$E_{\mathbf{Z}|\mathbf{Y},\Theta^{(t)}}(\mathbf{Z}) = \left(\mathbf{Y}'\left(\mathbf{B}^{(t)}\right)^T + \frac{1}{S_Z} \mathbf{CA}^{(t)} \right) \left(\mathbf{B}^{(t)}\left(\mathbf{B}^{(t)}\right)^T + \frac{1}{S_Z} \mathbf{I} \right)^{-1} \tag{13}$$

Here, $\mathbf{Y}' \in \mathbb{R}^{G\times N}$ consists of N column vectors $\mathbf{y}'_n \in \mathbb{R}^G$, each defined as:

$$\mathbf{y}'_n = \mathbf{y}_n - \mathbf{o}_r^{(t)} - \Delta\mathbf{o}_{i(n)}^{(t)} - \Delta\mathbf{Z}_{i(n)}^{(t)} \mathbf{b}_n^{(t)} - s_n^{(t)} \mathbf{1}_G$$

Note that, since each column $\mathbf{z}_k$ of **Z** has a multivariate normal posterior distribution, its mean coincides with its mode. We use this property to approximate the mean of **Z** when it is restricted to have orthogonal columns; i.e., we replace $E_{\mathbf{Z}|\mathbf{Y},\Theta}(\mathbf{Z})$ with $\mathbf{Z}_{\mathrm{MAP}}(\mathbf{Y},\Theta^{(t)})$ with orthogonality constraint:

$$E_{\mathbf{Z}|\mathbf{Y},\Theta^{(t)}}(\mathbf{Z}) \cong \mathbf{Z}_{MAP}\left(\mathbf{Y},\Theta^{(t)}\right)$$

$$= \arg\min_{\mathbf{Z}|\forall k\neq k'\, (\mathbf{Z}^T\mathbf{Z})_{k,k'}=0} \left[\frac{1}{2\sigma^2} \sum_{n\in\{1,\dots,N\}} \left\| \mathbf{y}_n - \mathbf{o}_r - \Delta\mathbf{o}_{i(n)} - \left(\mathbf{Z} + \Delta\mathbf{Z}_{i(n)}\right)\mathbf{b}_n - s_n \mathbf{1}_G \right\|_2^2 + \frac{1}{2\sigma^2 S_Z} \|\mathbf{Z} - \mathbf{CA}\|_F^2 \right]$$

$$= \arg\min_{\mathbf{Z}|\forall k\neq k'\, (\mathbf{Z}^T\mathbf{Z})_{k,k'}=0} \left[\sum_{n\in\{1,\dots,N\}} \left\| \mathbf{y}_n - \mathbf{o}_r - \Delta\mathbf{o}_{i(n)} - \left(\mathbf{Z} + \Delta\mathbf{Z}_{i(n)}\right)\mathbf{b}_n - s_n \mathbf{1}_G \right\|_2^2 + \frac{1}{S_Z} \|\mathbf{Z} - \mathbf{CA}\|_F^2 \right]$$

This can be solved similar to **section 1.1.1**, with the only difference that, here, we define $\mathbf{Y}''$ as:

$$\mathbf{Y}'' = \left[\mathbf{Y}' \quad \frac{1}{\sqrt{S_Z}} \mathbf{CA} \right]$$

1.2.2 Solving σ^2

We maximize the function Q with respect to σ^2:

$$\widehat{\sigma^2} = \arg\max_{\sigma^2} Q\left(\Theta|\Theta^{(t)}\right) = \arg\max_{\sigma^2} \left[E_{\mathbf{Z}|\mathbf{Y},\Theta^{(t)}}[\log f(\mathbf{Y}|\Theta,\mathbf{Z})] + E_{\mathbf{Z}|\mathbf{Y},\Theta^{(t)}}[\log f(\mathbf{Z}|\Theta)] + \log\, g(\Theta) \right]$$

$$= \arg\min_{\sigma^2} \left[-E_{\mathbf{Z}|\mathbf{Y},\Theta^{(t)}}[\log f(\mathbf{Y}|\Theta,\mathbf{Z})] - E_{\mathbf{Z}|\mathbf{Y},\Theta^{(t)}}[\log f(\mathbf{Z}|\Theta)] - \log\, g(\Theta) \right]$$

Using a procedure similar to the previous section, we can show that:

$$-E_{\mathbf{Z}|\mathbf{Y},\Theta^{(t)}}[\log f(\mathbf{Z}|\Theta)] = \frac{GK}{2}\log(2\pi\sigma^2 S_Z) + \frac{1}{2\sigma^2 S_Z}E_{\mathbf{Z}|\mathbf{Y},\Theta^{(t)}}\left[\sum_{g=1}^{G}\sum_{k=1}^{K}\left(z_{g,k} - (\mathbf{CA})_{g,k}\right)^2\right]$$
$$= \frac{GK}{2}\log(2\pi\sigma^2 S_Z) + \frac{1}{2\sigma^2 S_Z}\sum_{g=1}^{G}\sum_{k=1}^{K}\left(E_{\mathbf{Z}|\mathbf{Y},\Theta^{(t)}}\left(z_{g,k}\right) - (\mathbf{CA})_{g,k}\right)^2$$
$$+\frac{1}{2\sigma^2 S_Z}\sum_{g=1}^{G}\sum_{k=1}^{K}\mathrm{Var}_{\mathbf{Z}|\mathbf{Y},\Theta^{(t)}}\left(z_{g,k}\right)$$

The variance of each element of **Z**, conditional on **Y** and the current estimates of the parameters $\Theta^{(t)}$, is given by:

$$\mathrm{Var}_{\mathbf{Z}|\mathbf{Y},\Theta^{(t)}}\left(z_{g,k}\right) = \left(\sigma^{(t)}\right)^2\left[\left(\mathbf{B}^{(t)}\left(\mathbf{B}^{(t)}\right)^T + \frac{1}{S_Z}\mathbf{I}\right)^{-1}\right]_{k,k}$$

Therefore:

$$-E_{\mathbf{Z}|\mathbf{Y},\Theta^{(t)}}[\log f(\mathbf{Z}|\Theta)]$$
$$= \frac{GK}{2}\log(2\pi\sigma^2 S_Z) + \frac{1}{2\sigma^2 S_Z}\sum_{g=1}^{G}\sum_{k=1}^{K}\left(E_{\mathbf{Z}|\mathbf{Y},\Theta^{(t)}}\left(z_{g,k}\right) - (\mathbf{CA})_{g,k}\right)^2$$
$$+\frac{G\left(\sigma^{(t)}\right)^2}{2\sigma^2 S_Z}\sum_{k=1}^{K}\left[\left(\mathbf{B}^{(t)}\left(\mathbf{B}^{(t)}\right)^T + \frac{1}{S_Z}\mathbf{I}\right)^{-1}\right]_{k,k} \tag{14}$$

We can also use a similar approach to show that:

$$-E_{\mathbf{Z}|\mathbf{Y},\Theta^{(t)}}[\log f(\mathbf{Y}|\Theta,\mathbf{Z})] = \frac{GN}{2}\log(2\pi\sigma^2) + \frac{1}{2\sigma^2}E_{\mathbf{Z}|\mathbf{Y},\Theta^{(t)}}\left[\sum_{g=1}^{G}\sum_{n=1}^{N}\left(y'_{g,n} - (\mathbf{Zb}_n)_g\right)^2\right]$$
$$= \frac{GN}{2}\log(2\pi\sigma^2) + \frac{1}{2\sigma^2}\sum_{n=1}^{N}\sum_{g=1}^{G}\left[y'_{g,n} - \sum_{k=1}^{K}b_{k,n}E_{\mathbf{Z}|\mathbf{Y},\Theta^{(t)}}\left(z_{g,k}\right)\right]^2$$
$$+\frac{1}{2\sigma^2}\sum_{n=1}^{N}\sum_{g=1}^{G}\mathrm{Var}_{\mathbf{Z}|\mathbf{Y},\Theta^{(t)}}\left(\sum_{k=1}^{K}z_{g,k}b_{k,n}\right)$$

Here, $\mathbf{y'}_n$ is defined in the same way as in **section 1.2.1**. For the conditional variance of **Z**, we have:

$$\mathrm{Var}_{\mathbf{Z}|\mathbf{Y},\Theta^{(t)}}\left(\sum_{k=1}^{K}z_{g,k}b_{k,n}\right) = \sum_{k,k'=1}^{K}\mathrm{Cov}_{\mathbf{Z}|\mathbf{Y},\Theta^{(t)}}\left(z_{g,k}b_{k,n}, z_{g,k'}b_{k',n}\right) = \sum_{k,k'=1}^{K}b_{k,n}b_{k',n}\mathrm{Cov}_{\mathbf{Z}|\mathbf{Y},\Theta^{(t)}}\left(z_{g,k}, z_{g,k'}\right)$$
$$= \left(\sigma^{(t)}\right)^2\sum_{k,k'=1}^{K}b_{k,n}b_{k',n}\left[\left(\mathbf{B}^{(t)}\left(\mathbf{B}^{(t)}\right)^T + \frac{1}{S_Z}\mathbf{I}\right)^{-1}\right]_{k,k'}$$

Therefore:

$$-E_{\mathbf{Z}|\mathbf{Y},\Theta^{(t)}}[\log f(\mathbf{Y}|\Theta,\mathbf{Z})]$$
$$= \frac{GN}{2}\log(2\pi\sigma^2) + \frac{1}{2\sigma^2}\sum_{n=1}^{N}\sum_{g=1}^{G}\left[y'_{g,n} - \sum_{k=1}^{K}b_{k,n}E_{\mathbf{Z}|\mathbf{Y},\Theta^{(t)}}\left(z_{g,k}\right)\right]^2$$
$$+\frac{G\left(\sigma^{(t)}\right)^2}{2\sigma^2}\sum_{n=1}^{N}\sum_{k,k'=1}^{K}b_{k,n}b_{k',n}\left[\left(\mathbf{B}^{(t)}\left(\mathbf{B}^{(t)}\right)^T + \frac{1}{S_Z}\mathbf{I}\right)^{-1}\right]_{k,k'} \tag{15}$$

Finally, for the prior function *g*, we have:

$$-\log\ g(\Theta)=\frac{G}{2}\log(2\pi\sigma^2 S_o)+\frac{1}{2\sigma^2 S_o}\|\mathbf{o}_r-\mu_o\mathbf{1}_G\|_2^2+\frac{G}{2}\sum_{i\in\{1,\dots,Q\}}\log\left(2\pi\sigma^2 S_{\Delta o_i}\right)+\frac{1}{2\sigma^2}\sum_{i\in\{1,\dots,Q\}}\frac{1}{S_{\Delta o_i}}\|\Delta\mathbf{o}_i\|_2^2$$
$$+\frac{PK}{2}\log(2\pi\sigma^2 S_A)+\frac{1}{2\sigma^2 S_A}\|\mathbf{A}\|_F^2+\frac{GK}{2}\sum_{i\in\{1,\dots,Q\}}\log\left(2\pi\sigma^2 S_{\Delta z_i}\right)+\frac{1}{2\sigma^2}\sum_{i\in\{1,\dots,Q\}}\frac{1}{S_{\Delta z_i}}\|\Delta\mathbf{Z}_i\|_F^2 \quad (16)$$

Now, let's define the following sum:

$$\mathcal{S}=\sum_{n=1}^{N}\sum_{g=1}^{G}\left[y'_{g,n}-\sum_{k=1}^{K}b_{k,n}E_{\mathbf{Z}|\mathbf{Y},\Theta^{(t)}}(z_{g,k})\right]^2+G\left(\sigma^{(t)}\right)^2\sum_{n=1}^{N}\sum_{k,k'=1}^{K}b_{k,n}b_{k',n}\left[\left(\mathbf{B}^{(t)}\left(\mathbf{B}^{(t)}\right)^T+\frac{1}{S_Z}\mathbf{I}\right)^{-1}\right]_{k,k'}$$
$$+\frac{1}{S_Z}\sum_{g=1}^{G}\sum_{k=1}^{K}\left(E_{\mathbf{Z}|\mathbf{Y},\Theta^{(t)}}(z_{g,k})-(\mathbf{CA})_{g,k}\right)^2+\frac{1}{S_Z}G\left(\sigma^{(t)}\right)^2\sum_{k=1}^{K}\left[\left(\mathbf{B}^{(t)}\left(\mathbf{B}^{(t)}\right)^T+\frac{1}{S_Z}\mathbf{I}\right)^{-1}\right]_{k,k}$$
$$+\frac{1}{S_o}\|\mathbf{o}_r-\mu_o\mathbf{1}_G\|_2^2+\sum_{i\in\{1,\dots,Q\}}\frac{1}{S_{\Delta o_i}}\|\Delta\mathbf{o}_i\|_2^2+\frac{1}{S_A}\|\mathbf{A}\|_F^2+\sum_{i\in\{1,\dots,Q\}}\frac{1}{S_{\Delta z_i}}\|\Delta\mathbf{Z}_i\|_F^2 \quad (17)$$

Combining Eq. (14)-(17), we have:

$$\widehat{\sigma^2}=\frac{\mathcal{S}}{GN+PK+G+GQ+GK+GKQ} \quad (18)$$

1.2.3 Solving other parameters

As Eq. (14)-(17) in **section 1.2.2** suggest, maximization of the expectation function with respect to all parameters other than **A** and σ^2 can be done in the same way as in **sections 1.1.2-1.1.5**, with the exception that $\mathbf{Z}_r$ is replaced with its expectation, $E_{\mathbf{Z}|\mathbf{Y},\Theta}(\mathbf{Z})$.

1.3 Fitting the GEDI model with sample-level prior information

The sample-specific parameters that specify the distortions of the manifold in sample i, i.e, $\Delta\mathbf{o}_i$,$\Delta\mathbf{Z}_i$, can be expressed as probabilistic functions of $\mathbf{h}_i\in\mathbb{R}^L$, where $\mathbf{h}_i$ is a column vector whose elements represent the values of L variables for sample i. In this case, $\Delta\mathbf{o}_i$ as well as each column $\Delta\mathbf{z}_{i,k}$ are treated as latent variables with the following conditional probability function:

$$\Delta\mathbf{o}_i|\mathbf{R}_o\sim\mathcal{N}\left(\mathbf{R}_o\mathbf{h}_i,\sigma^2 S_{\Delta o_i}\mathbf{I}\right)$$
$$\mathbf{R}_o\sim\mathcal{N}\left(\mathbf{0},\sigma^2 S_{R_o}\mathbf{I}\right)$$
$$\Delta\mathbf{z}_{i,k}|\mathbf{R}_k\sim\mathcal{N}\left(\mathbf{R}_k\mathbf{h}_i,\sigma^2 S_{\Delta Z_i}\mathbf{I}\right)$$
$$\mathbf{R}_k\sim\mathcal{N}\left(\mathbf{0},\sigma^2 S_{R_k}\mathbf{I}\right)$$

$\mathbf{R}_o\in\mathbb{R}^{G\times L}$ and $\mathbf{R}_k\in\mathbb{R}^{G\times L}$ are matrices that represent the effects of the L variables on $\Delta\mathbf{o}_i$ and $\Delta\mathbf{z}_{i,k}$, respectively. Here, we will discuss how to solve this model in the absence of gene-level prior information, but the solutions from this section and **section 1.2** can be combined to solve the model in the presence of both gene-level and sample-level prior information. Here, the parameter set to be optimized includes:

$$\Theta=\{\mathbf{o}_r,\mathbf{Z}_r,\mathbf{R}_o,\mathbf{R}_1,\dots,\mathbf{R}_K,\mathbf{B},\sigma^2\}$$

For simplicity, let's define a set of matrices $\mathbf{\Delta}_i$, where for each $i\in\{1,\dots,Q\}$, the matrix $\mathbf{\Delta}_i\in\mathbb{R}^{G\times(K+1)}$ is the concatenation of $\Delta\mathbf{Z}_i$ and $\Delta\boldsymbol{o}_i$: $\mathbf{\Delta}_i=[\Delta\mathbf{Z}_i\ \Delta\mathbf{o}_i]$. Also, let's define matrix $\mathbf{\Delta}\in\mathbb{R}^{G\times Q(K+1)}$ to be the concatenation of all submatrices $\mathbf{\Delta}_i$, $\mathbf{\Delta}=[\mathbf{\Delta}_1\ \dots\ \mathbf{\Delta}_Q]$. Therefore, we need to optimize the following function:

$$\widehat{\Theta}_{\mathrm{MAP}}(\mathbf{Y})=\arg\max_\Theta f(\mathbf{Y}|\Theta)g(\Theta)=\arg\max_\Theta\int f(\mathbf{Y}|\Theta,\mathbf{\Delta})f(\mathbf{\Delta}|\Theta)d\mathbf{\Delta}\,g(\Theta)$$

Similar to **section 2**, we will use expectation maximization, where the expectation function Q is defined as:

$$Q\left(\Theta|\Theta^{(t)}\right)=E_{\mathbf{\Delta}|\mathbf{Y},\Theta^{(t)}}[\log f(\mathbf{Y}|\Theta,\mathbf{\Delta})]+E_{\mathbf{\Delta}|\mathbf{Y},\Theta^{(t)}}[\log f(\mathbf{\Delta}|\Theta)]+\log\ g(\Theta)$$

For simplicity, let's also define matrices $\mathbf{B}'$ and $\mathbf{B}'_i$ as:

$$\mathbf{B}' = \begin{bmatrix} \mathbf{B} \\ \mathbf{1}_N^T \end{bmatrix} \ \mathbf{B}_i' = \begin{bmatrix} \mathbf{B}_i \\ \mathbf{1}_{N_i}^T \end{bmatrix} \ i \in \{1, \ldots, Q\}$$

Here, $\mathbf{1}_N$ and $\mathbf{1}_{N_i}$ are $1 \times N$ and $1 \times N_i$ column-vectors of 1s, respectively, where N_i is the size of dataset i. We can now see that:

$$-E_{\boldsymbol{\Delta}|\mathbf{Y},\Theta^{(t)}}[\log f(\mathbf{Y}|\Theta, \boldsymbol{\Delta})] = \frac{GN}{2}\log(2\pi\sigma^2) + \frac{1}{2\sigma^2}E_{\boldsymbol{\Delta}|\mathbf{Y},\Theta^{(t)}}\left[\sum_{g=1}^{G}\sum_{n=1}^{N}\left(y'_{g,n} - (\boldsymbol{\Delta}\mathbf{b}'_n)_g\right)^2\right]$$

Here, $\mathbf{y'}_n$ is defined as:

$$\mathbf{y}'_n = \mathbf{y}_n - \mathbf{o}_r^{(t)} - \mathbf{Z}_r^{(t)}\mathbf{b}_n^{(t)} - s_n^{(t)}\mathbf{1}_G$$

Similar to section 1.2.2, we can show that:

$$\begin{aligned} &-E_{\boldsymbol{\Delta}|\mathbf{Y},\Theta^{(t)}}[\log f(\mathbf{Y}|\Theta, \boldsymbol{\Delta})] \\ &= \frac{GN}{2}\log(2\pi\sigma^2) + \frac{1}{2\sigma^2}\sum_{n=1}^{N}\sum_{g=1}^{G}\left[y'_{g,n} - \sum_{k=1}^{K+1} b'_{k,n}E_{\boldsymbol{\Delta}|\mathbf{Y},\Theta^{(t)}}\left(\delta_{i(n),g,k}\right)\right]^2 \\ &+ \frac{G\left(\sigma^{(t)}\right)^2}{2\sigma^2}\sum_{n=1}^{N}\sum_{k,k'=1}^{K} b'_{k,n}b'_{k',n}\left[\left(\mathbf{B}'_{i(n)}{}^{(t)}\left(\mathbf{B}'_{i(n)}{}^{(t)}\right)^T + \boldsymbol{\Lambda}_{i(n)}\right)^{-1}\right]_{k,k'} \end{aligned} \qquad (19)$$

Here, the prior precision matrix $\boldsymbol{\Lambda}_i$ is defined as:

$$\boldsymbol{\Lambda}_\mathrm{i} = \begin{bmatrix} \frac{1}{S_{\Delta Z_i}}\mathbf{I}_{K\times K} & \mathbf{0}_K \\ \mathbf{0}_K^T & \frac{1}{S_{\Delta o_i}} \end{bmatrix}$$

Also, similar to section 1.2.2, we can write:

$$\begin{aligned} &-E_{\boldsymbol{\Delta}|\mathbf{Y},\Theta^{(t)}}[\log f(\boldsymbol{\Delta}|\Theta)] \\ &= \frac{GK}{2}\sum_{i=1}^{Q}\log\left(2\pi\sigma^2 S_{\Delta Z_i}\right) + \sum_{i=1}^{Q}\frac{1}{2\sigma^2 S_{\Delta Z_i}}E_{\boldsymbol{\Delta}|\mathbf{Y},\Theta^{(t)}}\left[\sum_{g=1}^{G}\sum_{k=1}^{K}\left(\Delta z_{i,g,k} - (\mathbf{R}_k\mathbf{h}_i)_g\right)^2\right] \\ &+ \frac{G}{2}\sum_{i=1}^{Q}\log\left(2\pi\sigma^2 S_{\Delta o_i}\right) + \sum_{i=1}^{Q}\frac{1}{2\sigma^2 S_{\Delta o_i}}E_{\boldsymbol{\Delta}|\mathbf{Y},\Theta^{(t)}}\left[\sum_{g=1}^{G}\left(\Delta o_{i,g} - (\mathbf{R}_o\mathbf{h}_i)_g\right)^2\right] \end{aligned}$$

This can be further expanded to:

$$\begin{aligned} &-E_{\boldsymbol{\Delta}|\mathbf{Y},\Theta^{(t)}}[\log f(\boldsymbol{\Delta}|\Theta)] \\ &= \frac{GK}{2}\sum_{i=1}^{Q}\log\left(2\pi\sigma^2 S_{\Delta Z_i}\right) + \sum_{i=1}^{Q}\frac{1}{2\sigma^2 S_{\Delta Z_i}}\sum_{g=1}^{G}\sum_{k=1}^{K}\left(E_{\boldsymbol{\Delta}|\mathbf{Y},\Theta^{(t)}}\left(\Delta z_{i,g,k}\right) - (\mathbf{R}_k\mathbf{h}_i)_g\right)^2 \\ &+ \frac{G}{2}\sum_{i=1}^{Q}\log\left(2\pi\sigma^2 S_{\Delta o_i}\right) + \sum_{i=1}^{Q}\frac{1}{2\sigma^2 S_{\Delta o_i}}\sum_{g=1}^{G}\left(E_{\boldsymbol{\Delta}|\mathbf{Y},\Theta^{(t)}}\left(\Delta o_{i,g}\right) - (\mathbf{R}_o\mathbf{h}_i)_g\right)^2 \\ &+ \sum_{i=1}^{Q}\frac{1}{2\sigma^2 S_{\Delta Z_i}}\sum_{g=1}^{G}\sum_{k=1}^{K}\mathrm{Var}_{\boldsymbol{\Delta}|\mathbf{Y},\Theta^{(t)}}\left(\Delta z_{i,g,k}\right) + \sum_{i=1}^{Q}\frac{1}{2\sigma^2 S_{\Delta o_i}}\sum_{g=1}^{G}\mathrm{Var}_{\boldsymbol{\Delta}|\mathbf{Y},\Theta^{(t)}}\left(\Delta o_{i,g}\right) \end{aligned}$$

where:

$$\mathrm{Var}_{\boldsymbol{\Delta}|\mathbf{Y},\Theta^{(t)}}\left(\Delta z_{i,g,k}\right) = \left(\sigma^{(t)}\right)^2\left[\left(\mathbf{B}_i^{(t)}\left(\mathbf{B}_i^{(t)}\right)^T + \frac{1}{S_{\Delta Z_i}}\mathbf{I}\right)^{-1}\right]_{k,k}$$

$$\mathrm{Var}_{\boldsymbol{\Delta}|\mathbf{Y},\Theta^{(t)}}\left(\Delta o_{i,g}\right) = \frac{\left(\sigma^{(t)}\right)^2}{N_i + \frac{1}{S_{\Delta o_i}}}$$

Therefore:

$$-E_{\boldsymbol{\Delta}|\mathbf{Y},\Theta^{(t)}}[\log f(\boldsymbol{\Delta}|\Theta)]$$
$$= \frac{GK}{2}\sum_{i=1}^{Q}\log\left(2\pi\sigma^2 S_{\Delta Z_i}\right) + \frac{1}{2\sigma^2}\sum_{i=1}^{Q}\frac{1}{S_{\Delta Z_i}}\sum_{g=1}^{G}\sum_{k=1}^{K}\left(E_{\boldsymbol{\Delta}|\mathbf{Y},\Theta^{(t)}}\left(\Delta z_{i,g,k}\right) - (\mathbf{R}_k\mathbf{h}_i)_g\right)^2$$
$$+ \frac{G}{2}\sum_{i=1}^{Q}\log\left(2\pi\sigma^2 S_{\Delta o_i}\right) + \frac{1}{2\sigma^2}\sum_{i=1}^{Q}\frac{1}{S_{\Delta o_i}}\sum_{g=1}^{G}\left(E_{\boldsymbol{\Delta}|\mathbf{Y},\Theta^{(t)}}\left(\Delta o_{i,g}\right) - (\mathbf{R}_o\mathbf{h}_i)_g\right)^2$$
$$+ \frac{G\left(\sigma^{(t)}\right)^2}{2\sigma^2}\sum_{i=1}^{Q}\frac{1}{S_{\Delta Z_i}}\sum_{k=1}^{K}\left[\left(\mathbf{B}_i^{(t)}\left(\mathbf{B}_i^{(t)}\right)^T + \frac{1}{S_{\Delta Z_i}}\mathbf{I}\right)^{-1}\right]_{k,k} + \frac{G\left(\sigma^{(t)}\right)^2}{2\sigma^2}\sum_{i=1}^{Q}\frac{1}{S_{\Delta o_i}\left(N_i + \frac{1}{S_{\Delta o_i}}\right)} \tag{20}$$

Finally, for the prior function g, we have:

$$-\log\ g(\Theta) = \frac{G}{2}\log(2\pi\sigma^2 S_o) + \frac{1}{2\sigma^2 S_o}\|\mathbf{o}_r - \mu_o\mathbf{1}_G\|_2^2 + \frac{GL}{2}\log\left(2\pi\sigma^2 S_{R_o}\right) + \frac{1}{2\sigma^2 S_{R_o}}\|\mathbf{R}_o\|_2^2 + \frac{GK}{2}\log(2\pi\sigma^2 S_Z)$$
$$+ \frac{1}{2\sigma^2 S_Z}\|\mathbf{Z}_r\|_F^2 + \frac{GL}{2}\sum_{k\in\{1,\ldots,K\}}\log\left(2\pi\sigma^2 S_{R_k}\right) + \frac{1}{2\sigma^2}\sum_{k\in\{1,\ldots,K\}}\frac{1}{S_{R_k}}\|\mathbf{R}_k\|_F^2 \tag{21}$$

Note that the equations above require calculation of the expectation for $\boldsymbol{\Delta}_i$=[$\Delta\mathbf{Z}_i$ $\Delta\mathbf{o}_i$], which is given by the equation below:

$$E_{\boldsymbol{\Delta}|\mathbf{Y},\Theta^{(t)}}(\boldsymbol{\Delta}_i) = \left(\mathbf{Y}'\left(\mathbf{B'}_i^{(t)}\right)^T + \frac{1}{S_{\Delta Z_i}}\mathbf{R}_k^{(t)}\mathbf{h}_i\right)\left(\mathbf{B'}_i^{(t)}\left(\mathbf{B'}_i^{(t)}\right)^T + \frac{1}{S_{\Delta Z_i}}\mathbf{I}\right)^{-1} \tag{22}$$

1.3.1 Solving $\mathbf{R}_o$

Let's first define matrices $\mathbf{H}'$ and $\Delta\mathbf{O}'$ as follows:

$$\mathbf{H}'_{\Delta\boldsymbol{O}} = \left[\frac{1}{\sqrt{S_{\Delta o_1}}}\mathbf{h}_1 \quad \ldots \quad \frac{1}{\sqrt{S_{\Delta o_Q}}}\mathbf{h}_Q\right]$$
$$\Delta\mathbf{O}' = \left[\frac{1}{\sqrt{S_{\Delta o_1}}}E_{\boldsymbol{\Delta}|\mathbf{Y},\Theta^{(t)}}(\Delta\mathbf{o}_1) \quad \ldots \quad \frac{1}{\sqrt{S_{\Delta o_Q}}}E_{\boldsymbol{\Delta}|\mathbf{Y},\Theta^{(t)}}\left(\Delta\mathbf{o}_Q\right)\right]$$

Then, combining Eq. (19)-(21) and minimizing with respect to $\mathbf{R}_o$, we can see that:

$$\mathbf{R}_o = \Delta\mathbf{O}'\mathbf{H}'^T_{\Delta\mathbf{O}}\left(\mathbf{H}'_{\Delta\mathbf{O}}\mathbf{H}'^T_{\Delta\mathbf{O}} + \frac{1}{S_{R_o}}\mathbf{I}\right)^{-1} \tag{23}$$

1.3.2 Solving $\mathbf{R}_k$

Let's first redefine matrix $\mathbf{H}'$ as follows:

$$\mathbf{H}'_{\Delta Z} = \left[\frac{1}{\sqrt{S_{\Delta Z_1}}}\mathbf{h}_1 \quad \ldots \quad \frac{1}{\sqrt{S_{\Delta Z_Q}}}\mathbf{h}_Q\right]$$

Also, for each $k\in\{1,\ldots,Q\}$, we will define the matrix $\Delta\mathbf{Z}'_k$ as follows:

$$\Delta\mathbf{Z}'_k = \left[\frac{1}{\sqrt{S_{\Delta Z_1}}}E_{\boldsymbol{\Delta}|\mathbf{Y},\Theta^{(t)}}\left(\Delta\mathbf{z}_{1,k}\right) \quad \ldots \quad \frac{1}{\sqrt{S_{\Delta Z_Q}}}E_{\boldsymbol{\Delta}|\mathbf{Y},\Theta^{(t)}}\left(\Delta\mathbf{z}_{Q,k}\right)\right]$$

Combining Eq. (19)-(21) and minimizing with respect to each $\mathbf{R}_k$, we can see that:

$$\mathbf{R}_k = \Delta\mathbf{Z}'_k\mathbf{H}'^T_{\Delta Z}\left(\mathbf{H}'_{\Delta Z}\mathbf{H}'^T_{\Delta Z} + \frac{1}{S_{R_k}}\mathbf{I}\right)^{-1} \tag{24}$$

1.3.3 Solving σ^2

Let's define the following sum:

$$\mathcal{S} = \sum_{n=1}^{N}\sum_{g=1}^{G}\left[y'_{g,n} - \sum_{k=1}^{K+1} b'_{k,n}E_{\boldsymbol{\Delta}|\mathbf{Y},\Theta^{(t)}}\left(\delta_{i(n),g,k}\right)\right]^2$$

$$+ G\left(\sigma^{(t)}\right)^2 \sum_{n=1}^{N}\sum_{k,k'=1}^{K} b'_{k,n}b'_{k',n}\left[\left(\mathbf{B}'_{i(n)}{}^{(t)}\left(\mathbf{B}'_{i(n)}{}^{(t)}\right)^T + \boldsymbol{\Lambda}_{i(n)}\right)^{-1}\right]_{k,k'}$$

$$+ \sum_{i=1}^{Q}\frac{1}{S_{\Delta Z_i}}\sum_{g=1}^{G}\sum_{k=1}^{K}\left(E_{\boldsymbol{\Delta}|\mathbf{Y},\Theta^{(t)}}\left(\Delta z_{i,g,k}\right) - (\mathbf{R}_k\mathbf{h}_i)_g\right)^2 + \sum_{i=1}^{Q}\frac{1}{S_{\Delta o_i}}\sum_{g=1}^{G}\left(E_{\boldsymbol{\Delta}|\mathbf{Y},\Theta^{(t)}}\left(\Delta o_{i,g}\right) - (\mathbf{R}_o\mathbf{h}_i)_g\right)^2$$

$$+ G\left(\sigma^{(t)}\right)^2\sum_{i=1}^{Q}\frac{1}{S_{\Delta Z_i}}\sum_{k=1}^{K}\left[\left(\mathbf{B}_i^{(t)}\left(\mathbf{B}_i^{(t)}\right)^T + \frac{1}{S_{\Delta Z_i}}\boldsymbol{I}\right)^{-1}\right]_{k,k} + G\left(\sigma^{(t)}\right)^2\sum_{i=1}^{Q}\frac{1}{S_{\Delta o_i}\left(N_i + \frac{1}{S_{\Delta o_i}}\right)} + \frac{1}{S_Z}\|\mathbf{Z}_r\|_F^2$$

$$+ \frac{1}{S_o}\|\mathbf{o}_r - \mu_o\mathbf{1}_G\|_2^2$$

Combining Eq. (19)-(21), we have:

$$\widehat{\sigma^2} = \frac{\mathcal{S}}{GN + G + GQ + GK + GKQ + GL(K+1)} \tag{25}$$

1.3.4 Solving other parameters

As Eq. (19)-(21) suggest, maximization of the expectation function with respect to all parameters other than $\mathbf{R}_o$, $\mathbf{R}_k$, and σ^2 can be done in the same way as in **sections 1.1.2-1.1.5**, with the exception that $\Delta\mathbf{Z}_i$ and $\Delta\mathbf{o}_i$ are replaced with their expectations, $E_{\boldsymbol{\Delta}|\mathbf{Y},\Theta}(\Delta\mathbf{Z}_i)$ and $E_{\boldsymbol{\Delta}|\mathbf{Y},\Theta}(\Delta\mathbf{o}_i)$, respectively.

1.4 Fitting the GEDI model to UMI counts

In this section, we will discuss how the GEDI model can be fitted directly to the unnormalized, raw UMI count matrix $\mathbf{M}\in\mathbb{Z}^{G\times N}$. GEDI considers the following generative model for raw UMI counts:

$$m_{g,n} \sim \mathrm{Pois}(e^{y_{g,n}})$$
$$\mathbf{y}_n|\boldsymbol{\mu}_n(\Theta) \sim \mathcal{N}(\boldsymbol{\mu}_n(\Theta), \sigma^2\mathbf{I})$$
$$\Theta \sim g(\Theta)$$

Here, $\boldsymbol{\mu}_n(\Theta)$ is a function that returns the model-predicted vector $\boldsymbol{\mu}_n$ for cell n given the model parameters Θ — for the case with no gene-level or sample-level prior information, this is defined as:

$$\boldsymbol{\mu}_n(\Theta) = \mathbf{o}_r + \Delta\mathbf{o}_{i(n)} + \left(\mathbf{Z}_r + \Delta\mathbf{Z}_{i(n)}\right)\mathbf{b}_n + s_n\mathbf{1}_G \tag{26}$$

When gene-level and sample-level prior information is given, the solution can be easily derived from the same concepts described in this section.

Here, the model parameter set includes:

$$\Theta = \left\{\mathbf{o}_r, \mathbf{Z}_r, \Delta\mathbf{o}_1, \ldots, \Delta\mathbf{o}_Q, \Delta\mathbf{Z}_1, \ldots, \Delta\mathbf{Z}_Q, \mathbf{B}, \sigma^2, \mathbf{s}\right\}$$

To obtain the MAP estimate for the parameters of this hierarchical model (in which $\mathbf{y}_n$ is a latent variable), we want to maximize the following density function:

$$\widehat{\Theta}_{\mathrm{MAP}}(\mathbf{M}) = \arg\max_\Theta P(\mathbf{M}|\Theta)g(\Theta) = \arg\max_\Theta \int P(\mathbf{M}|\Theta,\mathbf{Y})f(\mathbf{Y}|\Theta)d\mathbf{Y}\,g(\Theta)$$
$$= \arg\max_\Theta \int P(\mathbf{M}|\mathbf{Y})f(\mathbf{Y}|\Theta)d\mathbf{Y}\,g(\Theta)$$

We will achieve this by expectation maximization, where the expectation function Q is:

$$Q\left(\Theta|\Theta^{(t)}\right) = E_{\mathbf{Y}|\mathbf{M},\Theta^{(t)}}[\log P(\mathbf{M}|\mathbf{Y})] + E_{\mathbf{Y}|\mathbf{M},\Theta^{(t)}}[\log f(\mathbf{Y}|\Theta)] + \log g(\Theta)$$

Note that, when maximizing the above function relative to Θ, the first part can be ignored, which means that:

$$\arg\max_\Theta Q\left(\Theta\middle|\Theta^{(t)}\right) = \arg\max_\Theta \left[E_{\mathbf{Y}|\mathbf{M},\Theta^{(t)}}[\log f(\mathbf{Y}|\Theta)] + \log g(\Theta)\right]$$
$$= \arg\min_\Theta \left[-E_{\mathbf{Y}|\mathbf{M},\Theta^{(t)}}[\log f(\boldsymbol{Y}|\Theta)] - \log g(\Theta)\right]$$

Let's expand the first part:

$$-E_{\mathbf{Y}|\mathbf{M},\Theta^{(t)}}[\log P(\mathbf{Y}|\Theta)] = \frac{NG}{2}\log(2\pi\sigma^2) + \frac{1}{2\sigma^2}\sum_{g=1}^{G}\sum_{n=1}^{N} E_{\mathbf{Y}|\mathbf{M},\Theta^{(t)}}\left[y_{g,n}^2 - 2y_{g,n}\mu_{g,n}(\Theta) + \mu_{g,n}^2(\Theta)\right]$$
$$= \frac{NG}{2}\log(2\pi\sigma^2) + \frac{1}{2\sigma^2}\sum_{g=1}^{G}\sum_{n=1}^{N}\left[E_{\mathbf{Y}|\mathbf{M},\Theta^{(t)}}\left(y_{g,n}\right) - \mu_{g,n}(\Theta)\right]^2 + \frac{1}{2\sigma^2}\sum_{g=1}^{G}\sum_{n=1}^{N}\mathrm{Var}_{\mathbf{Y}|\mathbf{M},\Theta^{(t)}}\left(y_{g,n}\right)$$

Therefore:

$$\arg\max_\Theta Q\left(\Theta\middle|\Theta^{(t)}\right)$$
$$= \arg\min_\Theta \left[\frac{NG}{2}\log(2\pi\sigma^2) + \frac{1}{2\sigma^2}\sum_{g=1}^{G}\sum_{n=1}^{N}\left[E_{\mathbf{Y}|\mathbf{M},\Theta^{(t)}}\left(y_{g,n}\right) - \mu_{g,n}(\Theta)\right]^2 - \log g(\Theta) + \frac{1}{2\sigma^2}\sum_{g=1}^{G}\sum_{n=1}^{N}\mathrm{Var}_{\mathbf{Y}|\mathbf{M},\Theta^{(t)}}\left(y_{g,n}\right)\right] \tag{27}$$

1.4.1 Solving parameters other than σ^2

Eq. (27) suggests that all paramters can be estimated in the same way as explained in the **sections 1.1**, **1.2**, or **1.3** (depending on the presence of prior information), with the difference that $y_{g,n}$ should be replaced with its expectation given the UMI count $m_{g,n}$ and the current parameter estimates $\Theta^{(t)}$. Therefore, we will describe here how to obtain this expectation:

$$E_{\mathbf{Y}|\mathbf{M},\Theta^{(t)}}\left(y_{g,n}\right) = \int_{-\infty}^{+\infty} f\left(y_{g,n}\middle|m_{g,n}, \mu_{g,n}(\Theta)\right) y_{g,n} dy_{g,n}$$

For simplicity, in what follows, we drop the subscripts g,n and simply write:

$$E_{\mathbf{Y}|\mathbf{M},\Theta^{(t)}}(y) = \int_{-\infty}^{+\infty} f\left(y\middle|m, \mu(\Theta)\right) y dy$$

Here, $f(y|m,\mu(\Theta))$ is the posterior probability density function given the observed count m and the model estimate $\mu(\Theta)$, which can be expanded as follows:

$$f\left(y\middle|m,\mu(\Theta)\right) = \frac{P(m|y)f\left(y\middle|\mu(\Theta)\right)}{\int_{-\infty}^{+\infty} P(m|u)f\left(u\middle|\mu(\Theta)\right)du}$$
$$P(m|y) = \frac{(e^y)^m e^{-e^y}}{m!}$$
$$f\left(y\middle|\mu(\Theta)\right) = \frac{e^{-\frac{1}{2\sigma^2}\left(y-\mu(\Theta)\right)^2}}{\sigma\sqrt{2\pi}}$$

This leads to:

$$E_{\mathbf{Y}|\mathbf{M},\Theta^{(t)}}(y) = c\int_{-\infty}^{+\infty} (e^y)^m e^{-e^y} e^{-\frac{1}{2\sigma^2}\left(y-\mu(\Theta)\right)^2} y dy = c\int_{-\infty}^{+\infty} e^{ym-e^y-\frac{1}{2\sigma^2}\left(y-\mu(\Theta)\right)^2} y dy$$

where c is a normalizing factor.

We use Laplace's method to approximate this integral, which leads to:

$$E_{\mathbf{Y}|\mathbf{M},\Theta^{(t)}}(y) \cong \arg\max_y \left[ym - e^y - \frac{1}{2\sigma^2}\left(y-\mu(\Theta)\right)^2\right] = \arg\max_y \left[y2\sigma^2 ym - 2\sigma^2 e^y - \left(y-\mu(\Theta)\right)^2\right]$$

By setting to zero the first derivative with respect to y, we have:

$$2\sigma^2 m - 2\sigma^2 e^y - 2\left(y-\mu(\Theta)\right) = 0$$
$$\sigma^2 e^y + y - (\mu(\Theta) + \sigma^2 m) = 0$$

$$y = -\mathrm{W}\left(\sigma^2 e^{\mu(\Theta)+\sigma^2 m}\right) + \mu(\Theta) + \sigma^2 m$$

Therefore:

$$E_{\boldsymbol{Y}|\boldsymbol{M},\Theta^{(t)}}(y) \cong= -W_0\left(\sigma^2 e^{\mu(\Theta)+\sigma^2 m}\right) + \mu(\Theta) + \sigma^2 m$$

where W_0 is the principal branch of the Lambert W function. While this equation provides an exact solution, it is computationally expensive to calculate the Lambert W function. Furthermore, when $\mu(\Theta)+\sigma^2 m$ is large, it is not possible to compute its exponential. Therefore, we use an alternative approach to approximate y based on Halley's method.

Consider again the following equation:

$$h(y) = \sigma^2 e^y + y - (\mu(\Theta) + \sigma^2 m)$$

We can use Halley's method to update y iteratively to find the root of h, using the equation:

$$y_{t+1} = y_t - \frac{2h(y_t)h'(y_t)}{2[h'(y_t)]^2 - h(y_t)h''(y_t)}$$

$$h'(y) = \sigma^2 e^y + 1$$

$$h''(y) = \sigma^2 e^y$$

In practice, we have found that this iterative procedure quickly converges on the solution for y. Note that calculating h and its derivatives requires the calculation of e^y. Since y is the logarithm of the expected read counts, e^y is expected to remain in a range that is feasible to compute. However, it is also possible to rearrange the iterative equation as below:

$$y_{t+1} = y_t - \frac{2[e^{-y_t}h(y_t)][e^{-y_t}h'(y_t)]}{2[e^{-y_t}h'(y_t)]^2 - [e^{-y_t}h(y_t)][e^{-y_t}h''(y_t)]}$$

$$e^{-y_t}h(y_t) = \sigma^2 + e^{-y_t}(y_t - \mu(\Theta) - \sigma^2 m)$$

$$e^{-y_t}h'(y_t) = \sigma^2 + e^{-y_t}$$

$$e^{-y_t}h''(y_t) = \sigma^2$$

This provides the possibility of using the first set of equations when y is negative, and the second set when y is positive, in order to avoid calculation of the exponential of large positive numbers.

1.4.2 Solving σ^2

Eq. (27) suggests that, to solve σ^2, the same equations as those presented in sections 1.1.6, 1.2.2, or 1.3.3 can be used (depending on the presence of prior information), with the only difference that the sum $\mathcal{S}$ should be modified to add the following term:

$$\mathcal{S}' = \mathcal{S} + \sum_{n=1}^{N} \mathrm{Var}_{\mathbf{Y}|\mathbf{M},\Theta^{(t)}}(y_{j,n})$$

To obtain the variance of $y_{g,n}$ given the counts $m_{g,n}$ and the current model parameters, again we use the Laplace's approximation, where variance is estimated as the negative reciprocal of the second derivative of the h function at its mode, where h is defined as:

$$h(y) = ym - e^y - \frac{1}{2\sigma^2}\left(y - \mu(\Theta)\right)^2$$

$$h'(y) = m - e^y - \frac{1}{\sigma^2}\left(y - \mu(\Theta)\right)$$

$$h''(y) = -e^y - \frac{1}{\sigma^2} \Rightarrow -\frac{1}{h''(y)} = \frac{1}{e^y + \frac{1}{\sigma^2}}$$

Since the mode of the h function is the expected value of y (as described in **section 1.4.1**), we have:

$$\mathrm{Var}_{\mathbf{Y}|\mathbf{M},\Theta^{(t)}}(y_{j,n}) = \frac{1}{e^{E_{\mathbf{Y}|\mathbf{M},\Theta^{(t)}}(y_{j,n})} + \frac{1}{\sigma^{2(t)}}}$$

1.5 Fitting the GEDI model to paired UMI counts

In this section, we will discuss how the GEDI model can be fitted directly to a pair of unnormalized, raw UMI count matrices $\mathbf{M}_1 \in \mathbb{Z}^{G\times N}$ and $\mathbf{M}_2 \in \mathbb{Z}^{G\times N}$. We are interested in modeling the logarithm of odds ratio of observing a molecule from $\mathbf{M}_1$ vs. observing a molecule from $\mathbf{M}_2$. Accordingly, GEDI considers the following generative model for raw UMI counts:

$$m_{1,g,n} \sim B\left(m_{g,n}, \frac{1}{1+e^{-y_{g,n}}}\right)$$
$$m_{g,n} = m_{1,g,n} + m_{2,g,n}$$
$$\mathbf{y}_n | \boldsymbol{\mu}_n(\Theta) \sim \mathcal{N}(\boldsymbol{\mu}_n, \sigma^2 \mathbf{I})$$
$$\Theta \sim g(\Theta)$$

Here, $\boldsymbol{\mu}_n(\Theta)$ is similar to **section 1.4**, Eq. (26); each element $\mu_{n,g}$ represents the model-predicted log odds ratio of $m_{1,n,g}$ vs. $m_{2,n,g}$. B is the binomial distribution.

Following the same procedure as in **section 1.4**, we can see that, for estimation of the model parameters using expectation-maximization, we need to maximize the following function:

$$\arg\max_{\Theta} Q(\Theta|\Theta^{(t)})$$
$$= \arg\min_{\Theta} \left[\frac{NG}{2}\log(2\pi\sigma^2) + \frac{1}{2\sigma^2}\sum_{g=1}^{G}\sum_{n=1}^{N}\left[E_{\mathbf{Y}|\mathbf{M}_1,\mathbf{M},\Theta^{(t)}}(y_{g,n}) - \mu_{g,n}(\Theta)\right]^2 - \log g(\Theta) + \frac{1}{2\sigma^2}\sum_{g=1}^{G}\sum_{n=1}^{N}\mathrm{Var}_{\mathbf{Y}|\mathbf{M}_1,\mathbf{M},\Theta^{(t)}}(y_{g,n}) \right]$$

Therefore, the same concepts as those outlined in **sections 1.4.1** and **1.4.2** apply here, except that we need to calculate $E_{\mathbf{Y}|\mathbf{M}_1,\mathbf{M},\Theta(t)}(y_{g,n})$ and $\mathrm{Var}_{\mathbf{Y}|\mathbf{M}_1,\mathbf{M},\Theta(t)}(y_{g,n})$.

1.5.1 Obtaining the expectation of Y

Similar to section 1.4.1, we have:

$$E_{\mathbf{Y}|\mathbf{M}_1,\mathbf{M},\Theta^{(t)}}(y_{g,n}) = \int_{-\infty}^{+\infty} f\left(y_{g,n} \middle| m_{1,g,n}, m_{g,n}, \mu_{g,n}(\Theta)\right) y_{g,n} dy_{g,n}$$

For simplicity, in what follows, we drop the subscripts g,n and simply write:

$$E_{\mathbf{Y}|\mathbf{M}_1,\mathbf{M},\Theta^{(t)}}(y) = \int_{-\infty}^{+\infty} f\left(y \middle| m_1, m, \mu(\Theta)\right) y dy$$

Here, $f(y|m_1,m,\mu(\Theta))$ is the posterior probability density function given the observed counts m_1 and $m=m_1+m_2$ and the model estimate $\mu(\Theta)$:

$$f\left(y \middle| m_1, m, \mu(\Theta)\right) = \frac{P(m_1|m,y) f\left(y \middle| \mu(\Theta)\right)}{\int_{-\infty}^{+\infty} P(m_1|m,u) f\left(u \middle| \mu(\Theta)\right) du}$$
$$P(m_1|m,y) = \binom{m}{m_1}\left(\frac{1}{1+e^{-y}}\right)^{m_1}\left(\frac{1}{1+e^{y}}\right)^{m-m_1}$$
$$f\left(y \middle| \mu(\Theta)\right) = \frac{e^{-\frac{1}{2\sigma^2}(y-\mu(\Theta))^2}}{\sigma\sqrt{2\pi}}$$

This leads to:

$$E_{\mathbf{Y}|\mathbf{M}_1,\mathbf{M},\Theta^{(t)}}(y) = c\int_{-\infty}^{+\infty}\left(\frac{1}{1+e^{-y}}\right)^{m_1}\left(\frac{1}{1+e^{y}}\right)^{m-m_1} e^{-\frac{1}{2\sigma^2}(y-\mu(\Theta))^2} y dy =$$
$$= c\int_{-\infty}^{+\infty}(e^{y})^{m_1}(1+e^{y})^{-m} e^{-\frac{1}{2\sigma^2}(y-\mu(\Theta))^2} y dy$$

where c is a normalizing factor.

We use Laplace's method to approximate this integral, which leads to:

$$E_{\mathbf{Y}|\mathbf{M}_1,\mathbf{M},\Theta^{(t)}}(y) \cong \arg\max_y \left\{m_1 y - m\ln(1+e^y) - \frac{1}{2\sigma^2}\big(y-\mu(\Theta)\big)^2\right\}$$

(Note that the function that is to be maximized is concave, since its second derivative with respect to y is always negative).

By setting to zero the first derivative with respect to y, we have:

$$\frac{d}{dy}\left[m_1 y - m\ln(1+e^y) - \frac{1}{2\sigma^2}\big(y-\mu(\Theta)\big)^2\right] = m_1 - \frac{m}{1+e^{-y}} - \frac{y-\mu(\Theta)}{\sigma^2} = 0$$

With some rearrangements, this leads to:

$$\sigma^2 m_2 - \mu(\Theta) + y + (y - \mu(\Theta) - \sigma^2 m_1)e^{-y} = 0$$

This can be rewritten as:

$$h(y) = y - \alpha + (y-\beta)e^{-y} = 0$$

where α and β are defined as:

$$\alpha = \mu(\Theta) - \sigma^2 m_2$$
$$\beta = \mu(\Theta) + \sigma^2 m_1$$

We will find the root of the function h (above) using Halley's method:

$$h(y) = y - \alpha + (y-\beta)e^{-y}$$
$$h'(y) = 1 + e^{-y}(\beta - y + 1)$$
$$h''(y) = e^{-y}(y - \beta - 2)$$
$$y_{t+1} = y_t - \frac{2h(y_t)h'(y_t)}{2[h'(y_t)]^2 - h(y_t)h''(y_t)}$$

Note that when $-y$ is large, the solution above requires computation of e^{-y}, which can result in numerical instability or out of range results. We can instead use the following calculations:

$$y_{t+1} = y_t - \frac{2[e^{y_t}h(y_t)][e^{y_t}h'(y_t)]}{2[e^{y_t}h'(y_t)]^2 - [e^{y_t}h(y_t)][e^{y_t}h''(y_t)]}$$
$$e^{y_t}h(y_t) = e^{y_t}(y_t - \alpha) + y_{nt} - \beta$$
$$e^{y_t}h'(y_t) = e^{y_t} + \beta - y_t + 1$$
$$e^{y_t}h''(y_t) = y_t - \beta - 2$$

In practice, we have found that Halley's method may overshoot or undershoot in some cases. We resolve this problem by identifying an upper and lower bound for the root of function h, within which Halley's method results in convergence to the solution. It is easy to show that the root of h must be between α and β ($\alpha<\beta$) because $h(\alpha)<0$ and $h(\beta)>0$. Furthermore, within this range, $h'(y)>0$ (i.e. h is monotonically increasing), and $h''(y)<0$, meaning that Halley's method will not over/undershoot if initialized with α.

1.5.2 Obtaining the variance of **Y**

To obtain the variance of $y_{g,n}$ given the counts $m_{1,g,n}$ and $m_{2,g,n}$ and the current model parameters, again we use the Laplace's approximation, where variance is estimated as the negative reciprocal of the second derivative of the h function at its mode, where h is defined as:

$$h(y) = m_1 y - m\ln(1+e^y) - \frac{1}{2\sigma^2}\big(y-\mu(\Theta)\big)^2$$
$$h'(y) = m_1 - \frac{m}{1+e^{-y}} - \frac{y-\mu(\Theta)}{\sigma^2}$$
$$h''(y) = -\frac{me^{-y}}{(1+e^{-y})^2} - \frac{1}{\sigma^2}$$
$$-\frac{1}{h''(y)} = \frac{1}{\frac{me^{-y}}{(1+e^{-y})^2} + \frac{1}{\sigma^2}} = \frac{1}{\frac{me^{y}}{(1+e^{y})^2} + \frac{1}{\sigma^2}}$$

And since mode of the h function is the expected value of y, we have:

$$\mathrm{Var}_{\mathbf{Y}|\mathbf{M}_1,\mathbf{M},\Theta^{(t)}}(y_{g,n}) = \frac{1}{\frac{me^{-\left|E_{\mathbf{Y}|\mathbf{M}_1,\mathbf{M},\Theta^{(t)}}(y)\right|}}{\left(1+e^{-\left|E_{\mathbf{Y}|\mathbf{M}_1,\mathbf{M},\Theta^{(t)}}(y)\right|}\right)^2} + \frac{1}{\sigma^2_{(t)}}}$$

1.6 Choice of hyperparameters

The behavior of GEDI may be fine-tuned using its hyperparameters, which primarily represent the variances of the prior distributions of the model parameters. However, in the present work, we have not systematically explored the effects of increasing or decreasing various prior distribution variances, as well as potential metrics that may be used to fine-tune the hyperparameters for a given dataset. Instead, we have selected a set of predefined values based on what we would expect to infer from "random" gene expression data, i.e., data generated from a model in which all parameters are zero except for the model variance σ^2, as described below.

1.6.1 The prior distribution of $\mathbf{Z}_r$

Consider the matrix of observed gene expression **Y** generated from a model similar to that described in **section 1.1**, with the "null" parameter set Θ_0 in which all parameters, except σ^2, are zero. We then use **Y** to infer Θ using a block coordinate descent approach similar to that used by GEDI, with the exception that we use flat priors for each parameter. When inferring $\mathbf{Z}_r$ with the flat prior $U(-\infty,+\infty)$, each row $\mathbf{z}_{r,g,*}$ of $\mathbf{Z}_r$ will have the following posterior distribution, conditional on **Y** and all other model parameter estimates:

$$\mathbf{z}_{r,g,*}|\mathbf{Y},\widehat{\Theta}\backslash\{\widehat{\mathbf{Z}}_r\} \sim \mathcal{N}\left(\hat{\mathbf{z}}_{r,g,*}, \hat{\sigma}^2\left(\widehat{\mathbf{B}}\widehat{\mathbf{B}}^\top\right)^{-1}\right)$$

Here, $\hat{\mathbf{z}}_{r,g,*}$ is the ML estimate of $\mathbf{z}_{r,g,*}$ given **Y** and other model parameters. Note that **B** is restricted to have rows with L^2 norm of 1, as described in **section 1.1.5**. Furthermore, under the assumptions of the null model, the expected covariance of different rows of **B** is zero, leading to $E_{\mathbf{Y}|\Theta_0}(\widehat{\mathbf{B}}\widehat{\mathbf{B}}^\top)=\mathbf{I}$. Therefore:

$$E_{\mathbf{Y}|\Theta_0}\left(\mathrm{Cov}_{\mathbf{z}_{r,g,*}|\mathbf{Y},\widehat{\Theta}\backslash\{\hat{\mathbf{z}}_r\}}(\mathbf{z}_{r,g,*})\right) = \hat{\sigma}^2\mathbf{I}$$

In other words, each element of $z_{r,g,k}$ is expected to have a posterior normal distribution with mean $\hat{z}_{r,g,k}$ and variance σ^2. Conversely, we can infer that the ML estimate $\hat{z}_{r,g,k}$ is expected to be sampled from a normal distribution with mean 0 and variance σ^2. We use this distribution as the prior in the GEDI model:

$$\mathbf{z}_{r,*,k} \sim \mathcal{N}(\mathbf{0}, \sigma^2 S_Z \mathbf{I})$$

where S_Z is set to 1.

1.6.2 The prior distribution of $\Delta\mathbf{Z}_i$

Let's consider the same null model as the previous section. When inferring $\Delta\mathbf{Z}_i$ (for each $i\in\{1,\ldots,Q\}$) from data generated by this null model, each row $\Delta\mathbf{z}_{i,g,*}$ of $\Delta\mathbf{Z}_i$ will have the following posterior distribution if a flat prior $U(-\infty,+\infty)$ is used, conditional on **Y** and all other model parameter estimates:

$$\Delta\mathbf{z}_{i,g,*}|\mathbf{Y},\widehat{\Theta}\backslash\{\Delta\widehat{\mathbf{Z}}_i\} \sim \mathcal{N}\left(\Delta\hat{\mathbf{z}}_{i,g,*}, \hat{\sigma}^2\left(\widehat{\mathbf{B}}_i\widehat{\mathbf{B}}_i^\top\right)^{-1}\right)$$

Following the same assumptions as those of the previous section, we can see that $E_{\mathbf{Y}|\Theta_0}(\widehat{\mathbf{B}}_i\widehat{\mathbf{B}}_i^\top)=(N_i/N)\times\mathbf{I}$, where N_i is the number of cells in sample i and N is the total number of cells across all samples. This suggests the following prior:

$$\Delta\mathbf{z}_{i,*,k} \sim \mathcal{N}(\mathbf{0}, \sigma^2 S_{\Delta Z_i}\mathbf{I})$$

$$S_{\Delta Z_i} = \frac{N}{N_i}$$

1.6.3 The prior distribution of $\Delta\mathbf{o}_i$

Similar to the previous sections, we can see that, with a flat prior, the posterior distribution of each element of the $\Delta\mathbf{o}_i$ vector, when **Y** is generated from a null parameter set, is given by:

$$\Delta o_{i,g}|\mathbf{Y},\widehat{\Theta}\backslash\{\Delta\hat{\mathbf{o}}_i\} \sim \mathcal{N}\left(\Delta\hat{o}_{i,g}, \hat{\sigma}^2\frac{1}{N_i}\right)$$

Therefore, we use the following prior:

$$\Delta\mathbf{o}_i \sim \mathcal{N}(\mathbf{0}, \sigma^2 S_{\Delta o_i}\mathbf{I})$$

$$S_{\Delta o_i} = \frac{1}{N_i}$$

We note that $\Delta\mathbf{o}_i$ represents a simple linear batch correction that maps the centroids of the manifolds of the different samples to the centroid of the reference manifold. Therefore, when all samples are expected to have similar cell types, a less stringent prior can be placed on $\Delta\mathbf{o}_i$. In the benchmarking analyses presented in this paper, we use $S_{\Delta oi}$=1000/N_i to better accommodated the mean abundance differences that exist between multiple scRNA-seq technologies.

1.6.4 The prior distribution of **A**

In the presence of gene-level prior information (represented by matrix **C**), $\mathbf{Z}_r$ is modeled as:

$$\mathbf{z}_{r,k}|\mathbf{a}_k \sim \mathcal{N}(\mathbf{C}\mathbf{a}_k, \sigma^2 S_Z \mathbf{I})$$

Similar to the previous sections, we consider data generated from a null model in which **A**=**0**. When inferring **A** from this data with a flat prior, conditional on $\mathbf{Z}_r$ and σ^2, the posterior distribution of **A** is given by:

$$\mathbf{a}_k\big|\hat{\mathbf{Z}}_r, \hat{\sigma}^2 \sim \mathcal{N}(\hat{\mathbf{a}}_k, \hat{\sigma}^2 S_Z (\mathbf{C}^\top \mathbf{C})^{-1})$$

If **C** has orthonormal column vectors, then $\mathbf{C}^\top\mathbf{C}=\mathbf{I}$, and since S_z=1, the posterior distribution of each element of $\mathbf{a}_k$ will have a variance equal to σ^2. We can therefore use the following prior:

$$\mathbf{a}_k \sim \mathcal{N}(\mathbf{0}, \sigma^2 S_A \mathbf{I})$$
$$S_A = 1$$

GEDI uses this prior even when the provided matrix **C** does not have orthonormal column vectors. However, in this case, it uses singular value decomposition (SVD) to obtain $\mathbf{C}=\mathbf{U}\mathbf{\Sigma}\mathbf{V}^\top$. It then uses the matrix $\mathbf{C}'=\mathbf{U}$ instead of **C** in model fitting. Once the matrix $\mathbf{A}'$ is obtained by fitting the model with $\mathbf{C}'$ instead of **C**, **A** can be obtained as follows:

$$\mathbf{CA} = \mathbf{C}'\mathbf{A}'$$
$$\Rightarrow \mathbf{C}'\mathbf{\Sigma}\mathbf{V}^\top\mathbf{A} = \mathbf{C}'\mathbf{A}'$$
$$\Rightarrow \mathbf{\Sigma}\mathbf{V}^\top\mathbf{A} = \mathbf{A}'$$
$$\Rightarrow \mathbf{A} = \mathbf{V}\mathbf{\Sigma}^{-1}\mathbf{A}'$$

Note that this is equivalent to a regularized principal component regression.

1.6.5 The prior distributions of $\mathbf{R}_o$ and $\mathbf{R}_k$

In the presence of sample-level prior information (represented by vector $\mathbf{h}_i$ for each sample i), $\Delta\mathbf{o}_i$ is modeled as:

$$\Delta\mathbf{o}_i|\mathbf{R}_o \sim \mathcal{N}\big(\mathbf{R}_o\mathbf{h}_i, \sigma^2 S_{\Delta o_i}\mathbf{I}\big)$$

If we infer $\mathbf{R}_o$ with a flat prior, conditional on $\Delta\mathbf{O}$ (as defined in **section 1.3.1**) and σ^2 we have:

$$\mathbf{r}_{o,g,*}|\Delta\hat{\mathbf{O}}, \hat{\sigma}^2 \sim \mathcal{N}\left(\hat{\mathbf{r}}_{o,g,*}, \hat{\sigma}^2\big(\mathbf{H}'_{\Delta\mathbf{O}}\mathbf{H}'^{\top}_{\Delta\mathbf{O}}\big)^{-1}\right)$$

where $\mathbf{r}_{o,g,*}$ represents row g of matrix $\mathbf{R}_o$, and, similar to **section 1.3.1**, $\mathbf{H}'_{\Delta\boldsymbol{O}}$ is defined as:

$$\mathbf{H}'_{\Delta\boldsymbol{O}} = \left[\frac{1}{\sqrt{S_{\Delta o_1}}}\mathbf{h}_1 \quad \dots \quad \frac{1}{\sqrt{S_{\Delta o_Q}}}\mathbf{h}_Q\right]$$

If $\mathbf{H}'_{\Delta\boldsymbol{O}}$ has orthonormal row vectors, then similar to the previous section, we can see that the covariance of the posterior distribution of each row of $\mathbf{R}_o$ will be $\sigma^2\mathbf{I}$, which leads us to use the following prior:

$$\mathbf{R}_o \sim \mathcal{N}\big(\mathbf{0}, \sigma^2 S_{R_o}\mathbf{I}\big)$$
$$S_{R_o} = 1$$

A similar line of calculations will lead to the following prior matrix for Rk as long as $\mathbf{H}'_{\Delta\boldsymbol{Z}}$ has orthonormal rows:

$$\mathbf{R}_k \sim \mathcal{N}\big(\mathbf{0}, \sigma^2 S_{R_k}\mathbf{I}\big)$$
$$S_{R_k} = 1$$

GEDI uses SVD to convert $\mathbf{H}'_{\Delta\mathbf{O}}$ and $\mathbf{H}'_{\Delta\mathbf{Z}}$ to matrices with orthonormal rows, allowing it to use the above priors. The fitted matrices $\mathbf{R}'_o$ and $\mathbf{R}'_k$ are then converted to the original scale similar to the previous section.

2 Datasets and preprocessing

2.1.1 PBMC dataset

The PBMC dataset[1] is a collection of immune cell types profiled from peripheral blood from two human donors across six single-cell RNA-seq technologies (10x Chromimum v2 and v3, CEL-seq2, Drop-seq, inDrops, Seq-Well and Smart-seq2). The raw count matrices and metadata were retrieved using the SeuratData package (v0.1), using the accession code "pbmcsca" (v.3.0.0). The data can be downloaded at the original website repository[20] or at the GEO accession number GSE132044.

For QC, we kept cells that passed the following cutoffs: $\log_{10}$ total counts >2.5, $\log_{10}$ total features >2, and percentage of mitochondrial reads per cell <20%. To remove low-expressed genes, we kept genes that had more than five counts in more than three cells across the entire dataset. After filtering, the PBMC dataset contained 28,035 cells and 14,406 genes. To obtain normalized expression counts, we used the function multiBatchNorm from batchelor (v.1.6.3). For the integration benchmark, we used the combination of the technology and the donor variable as the 'batch' variable (14 samples).

2.1.2 Pancreas dataset

The Pancreas dataset is a collection of datasets from multiple studies that profiled human cells from the pancreas using various scRNAseq technologies. The raw count matrices and metadata were retrieved using the SeuratData package[21] (v0.1), using the accession code "panc8" (v.3.0.2). The original collection consisted of eight datasets (celseq, celseq2, fluidigmc1, indrop1, indrop2, indrop3, indrop4 and smartseq2). As we were interested in comparing the performance of GEDI when fitted directly to raw counts against the performance of other methods, we restricted the analysis to the datasets that contained raw count data available (indrop1, indrop2, indrop3, indrop4 and smartseq2). The Smart-seq2 dataset[2] contained five healthy donors and four donors with type 2 diabetes—the original processed data can be found at ArrayExpress accession number E-MTAB-5061. The inDrop datasets[3] contained four healthy donors, and the original processed data can be found at GEO accession number GSE84133.

For QC, we kept cells that passed the following cutoffs: $\log_{10}$ total counts >3 and $\log_{10}$ total features >2.8. To alleviate the effect of donors with low cell numbers, we removed samples that had less than 100 cells per donor. To remove low-expressed genes, we kept genes that had more than five counts in more than three cells across the entire dataset. After filtering, the Pancreas dataset contained 10,902 cells and 18,366 genes. To obtain normalized expression counts, we used the function multiBatchNorm from batchelor[22, 23] (v.1.6.3). For the integration benchmark, we used the donor (encoded as 'orig.ident' in the metadata) as the 'batch' variable (13 samples).

2.1.3 Tabula Muris BM dataset

The Tabula Muris BM dataset[4] is a collection of cell types profiled from the bone marrow of mice across two scRNA-seq technologies (10x Chromimum and Smart-seq2). Data in H5AD format for the 10x experiment was retrieved from ref[24]. We restricted our analysis to bone marrow cells derived from three female mice.

For QC, we kept cells that passed the following cutoffs: $\log_{10}$ total counts >3 and $\log_{10}$ total features >3. To remove low-expressed genes, we kept genes that had more than five counts in more than three cells across the entire dataset. After filtering, the Tabula Muris BM dataset contained 13,874 cells and 15,472 genes. To obtain normalized expression counts, we used the function multiBatchNorm from batchelor (v.1.6.3). For the integration benchmark, as the mice of origin were different across both scRNA-seq technologies, we used the mouse of origin (encoded as 'mouse.id' in the metadata) as the 'batch' variable (10 samples).

2.1.4 COVID-19 dataset

The COVID-19 dataset[7] profiled human peripheral blood samples from independent patient cohorts at two university medical centers in Germany. Samples from cohort 1 were profiled using 10x Chromium, while samples from cohort 2 were profiled using a microwell-based scRNA-seq system (Rhapsody). The dataset contains individuals diagnosed with mild and severe COVID-19, as well as healthy controls. Raw count data and metadata were retrieved from the processed Seurat objects, which were downloaded from the FastGenomics Portal; Cohort 1 dataset was downloaded

from[25], while Cohort 2 dataset was downloaded from[26]. The data is deposited at the European Genome-phenome Archive (EGA) under access number EGAS00001004571.

2.1.5 Faure dataset

Faure et al.[16] used scRNA-seq (Smart-seq2) to understand the developmental diversity that occurs in sensory neurogenesis in mice. The processed data was downloaded in LOOM format from the GEO accession number GSE150150, which contained intronic and exonic raw counts. Metadata was downloaded from the GitHub repository[27] associated with the article. Only cells that passed QC in the original article were used for analysis. To remove low-expressed genes, we kept genes that (a) had more than five counts in more than three cells across the entire dataset, (b) were expressed in more than one cell in the intronic counts, and (c) were expressed in more than one cell in the exonic counts. After filtering, the Faure dataset consisted of 2,245 cells and 14,725 genes.

2.1.6 La Manno dataset

La Manno et al.[17] used scRNA-seq (10x Chromium) to understand the kinetics of transcription in human embryonic glutamatergic neurogenesis. The processed data was downloaded in LOOM format from the authors' repository[28], which contained metadata, intronic and exonic counts. Only cells that passed QC in the original article were used for analysis. To remove low-expressed genes, we kept genes that (a) had more than five counts in more than three cells across the entire dataset, (b) were expressed in more than one cell in the intronic counts, and (c) were expressed in more than one cell in the exonic counts. After filtering, the La Manno dataset consisted of 1,720 cells and 5,037 genes.

2.1.7 Genga dataset

Genga et al.[8] performed a scRNA-seq based CRISRPi screening of 50 transcription factors in human embryonic stem cells. Processed data consisting of the raw count matrix, sgRNA matrix and cell metadata were downloaded from ref[29] provided by Holland et al.[30]. Only cells that passed QC in the Holland re-analysis were used. Normalized expression values were obtained using the logNormCounts from scran. After QC, the dataset consisted of 5,281 cells and 12,781 genes.

2.1.8 Tasic dataset

The Tasic dataset is a collection of two studies that profiled neocortex tissue in adult mice. The Tasic 2016 study[10] used the SMARTer Ultra Low RNA Kit and generated single-end reads, while the Tasic 2018 study[11] used the SMART-Seq v4 Ultra Low Input RNA Kit for Sequencing protocol and generated paired-end reads. FASTQ files were downloaded from the NCBI Short Read Archive (SRA) under accession numbers SRP061902 and SRP150473. Metadata for the Tasic 2016 dataset was retrieved from the GEO accession number GSE71585, as well as from the Supplementary Table 1 from Feng et al.[31], who performed a splicing-analysis of the two Tasic datasets. Metadata for the Tasic 2018 dataset was retrieved from the GEO accession number GSE115746.

Quantification of the reads that support exon inclusion and exon exclusion events was performed using the Quantas pipeline[32] (v.1.1.1). Alignment of the reads to the mm10 genome was performed using Olego[33, 34] (v.1.1.9). To quantify alternative splicing events, we inferred the transcript structure between paired-end reads using 'gapless' (this step was only performed for the paired-end data). Then, we quantified inclusion or exclusion read counts for cassette exons using the 'summarize_splicing_wrapper.pl' script from 'countit'.

For QC, we kept cells that passed the following cutoffs: $\log_{10}$ total counts >3, $\log_{10}$ total features >2, and percentage of mitochondrial reads per cell <20%. To remove low-expressed genes, we kept genes that had more than three counts in more than three cells across the entire dataset. After filtering, the COVID-19 dataset contained 197,039 cells and 13,205 genes. To obtain normalized expression counts, we used the function multiBatchNorm from batchelor (v.1.6.3). For the analysis of sample-to-sample variability with GEDI, the donor of origin was considered as the

‘sample’ variable, except for donors BN-10, BN-11 and BN-12—each of those three patients were profiled in severe and mild conditions, so a combination of the donor and the COVID status was used.

For QC, we filtered out cells that were classified as low-quality cells in the original metadata. Then, we restricted our analysis to cells that were classified as ‘Non-Neuronal’, ‘GABAergic’, ‘Glutamatergic’, and ‘Endothelial’ in the original metadata. Finally, we kept cells that passed the following cutoff: $\log_{10}$ total inclusion counts >4.5 and $\log_{10}$ total exclusion counts >4. To remove low-expressed cassette exons, we kept events that had more than 50 exon inclusion counts and more than 50 exon exclusion counts across the entire dataset. After filtering, the Tasic dataset contained 25,352 cells and 14,267 exon events. Normalized exclusion and inclusion counts were obtained using the normalizeCounts from scuttle.

For the integration tasks, for all methods except for GEDI-B and LIGER, we used the logarithm of a naïve estimate of the ratio of the inclusion and exclusion counts as input for each method. This ratio was calculated as $(1+\mathbf{M}'_1)/(1+\mathbf{M}'_2)$, where $\mathbf{M}'_1$ represents the normalized count matrix for the inclusion counts and $\mathbf{M}'_2$ represents the normalized count matrix for the exclusion counts. For LIGER, we used $(1+\mathbf{M}_1)/(2+\mathbf{M}_1+\mathbf{M}_2)$, where $\mathbf{M}_1$ represents the raw count matrix for the inclusion counts and $\mathbf{M}_2$ represents the raw count matrix for the exclusion counts. These choices were made based on the requirement for the input data of each method; for example, while most other methods can work with values that span negative and positive numbers, LIGER works with only positive numbers. For GEDI-B (GEDI with a binomial data generating distribution), we used the pair of raw inclusion and exclusion counts as input.

3 Integration methods

To compare the integration performance across methods, we ran each method using the same set of genes (all genes after filtering low-expressed genes) and five different values of K (where K represents the number of latent variables), including 20, 40, 60, 80, and 100. The details of each method can be found below:

3.1.1 GEDI

For the integration benchmark tasks (PBMC, Pancreas, and Tabula Muris BM), we ran GEDI using the raw counts and with default parameters, except that we set ‘oi_shrinkage=0.001’. The integrated embedding was retrieved using the function ‘svg.gedi’. For non-benchmark integration tasks, K=20 was used.

3.1.2 Seurat

To run Seurat[35, 36] (v.4.1.1), we followed the documentation available on the Seurat website[37]. We ran the Canonical Correlation Analysis (CCA) pipeline for integration in Seurat, which included the functions CreateSeuratObject, SplitObject(split.by=batch), NormalizeData, FindIntegrationAnchors and IntegrateData. To run Seurat with a specified gene list, these were indicated in the FindIntegrationAnchors with the ‘anchor.features’ parameter. PCA was computed on the integrated expression matrix returned by Seurat.

3.1.3 LIGER

To run LIGER[38] (v.1.0.0), we followed the documentation available on the LIGER GitHub repository[39]. We applied the pipeline used for the integration of multiple scRNA-seq datasets, which performs integrative nonnegative matrix factorization (iNMF). This included the createLiger, normalize, scaleNotCenter, optimizeALS and quantile_norm functions. The integrated embedding returned by LIGER was obtained from the ‘H.norm’ slot space in the LIGER object.

3.1.4 Harmony

To run Harmony[19, 40] (v.1.0), we followed the documentation available on the Harmony GitHub repository[41]. We first ran PCA on the normalized expression matrix of each dataset and then used the HarmonyMatrix function with arguments ‘do_pca=FALSE’ to obtain the integrated Harmony embeddings.

3.1.5 BBKNN

To run BBKNN[42] (v.1.3.12), we followed the integration tutorial on the GitHub repository[43], which suggested applying the pre-processing steps with scanpy[44] (v.1.9.1) and then performing integration with BBKNN. We ran the normalization steps (scanpy.pp.normalize_per_data and scanpy.pp.log1p) and computed PCA using scanpy.tl.pca. The integrated space was recovered using the bbknn.bbknn function, which constructs a batch-balanced neighborhood graph.

3.1.6 CSS

To run CSS[45] (simspec v.0.0.0.9000), we followed the documentation available in the GitHub repository[46]. CSS uses a processed Seurat Object, so we first created a Seurat object and followed the default scRNA-seq pipeline (CreateSeuratObject, NormalizeData, ScaleData, RunPCA). Then, we used the cluster_sim_spectrum from simspec, which returned CSS-integrated embeddings.

3.1.7 PCA

PCA was run using the rpca function from rsvd[47] (v.1.0.5) on the normalized expressed data.

3.1.8 scVI

To run scVI[48] (v.1.0.4), we followed the documentation available on the scVI website[49]. We first registered the AnnData object using the scvi.model.SCVI.setup_anndata function, using the raw counts as input. We then created the model using the scvi.model.SCVI function. After training, the integrated embedding was retrieved using the get_latent_representation method and nearest-neighbors and UMAP were calculated using scanpy.

3.1.9 Scanorama

To run Scanorama[50] (v.1.7.4), we followed the documentation available on its GitHub repository[51]. We ran the pre-processing steps with scanpy, including normalization (scanpy.pp.normalize_per_data and scanpy.pp.log1p) and PCA (scanpy.tl.pca). The original AnnData object was split per batch into a list of AnnData objects, which was input for the scanorama.correct_scanpy function. The returned list was merged and the integrated embeddings were retrieved from the merged AnnData object.

4 Metrics to compare integration performance

All metrics described below rely on identification of the neighborhood of each cell, followed by examination of the extent to which cells from different batches or different cell types mix together within the neighborhoods. It is desired to see the cells from different batches mix together within neighborhoods, while cells from different cell types should remain in separate neighborhoods. Identification of the neighborhoods relies on measuring the pairwise distances of the cells in the integrated space. Given that the integrated space is still high-dimensional (20-100 dimensions) and, therefore, Euclidean distances between pairs of points may become relatively homogenous (curse of dimensionality), we first generated a UMAP[52] embedding of the cells with the same number of dimensions as that of the original integrated space (K). The UMAP transformation leads to an emphasis on the local connectivity of the points. Furthermore, as previously shown[53], Euclidean distances of points in the UMAP embedding better correlate with their geodesic distances, especially when the data points are noisy, compared to Euclidean distances in, for example, PCA-projected data.

For all methods except BBKNN, the output of each method was an integrated embedding, from which we generated a UMAP embedding of dimension K, using the umap function from uwot[54] (v.0.1.10). For BBKNN, we ran UMAP on the batch-balanced neighborhood graph. The K-dimensional UMAP embedding was used as input for the calculation of the integration metrics, as described below.

4.1.1 Alignment Score

The alignment score was proposed by Butler et al.[55], with the goal of quantifying how well any group of data sets is aligned. The alignment score ranges from 0 to 1, and when applied to batch labels, a value closer to 1 denotes good mixing. We implemented the alignment score as an R function as defined previously[55]. The alignment score builds a k-nearest neighbor graph based on the cell's embedding; we used k=10. The alignment score was applied either to the cell type or batch labels. For the cell type labels, to ensure that a higher AS score means better cell type conservation, we subtracted it from 1 to give a final cell type AS score.

4.1.2 LISI

The local inverse Simpson's Index (LISI) was proposed by Korsunsky et al.[19], and consists of a measure to assess batch mixing (integration LISI) and cell-type separation (cell-type LISI). Integration LISI (iLISI) defines the effective number of batches in a neighborhood, and a score close to the expected number of batches denotes good mixing. We used the compute_lisi function from lisi[56] (v.1.0) on the cell type labels (cLISI) or on the batch labels (iLISI). The original LISI scores range from 1 to B (where B is the number of batches), so we applied the normalization procedure proposed by Luecken et al.[5], where the values of LISI were rescaled to range from 0 to 1. Specifically, scaled iLISI was calculated as [median(iLISI)–1]/[B–1], and scaled cLISI was calculated as [B–median(cLISI)]/[B–1].

4.1.3 kBET

kBET was developed by Büttner et al.[57] as a measure to quantify batch effects in scRNA-seq data. kBET uses a chi-squared test to assess the mixing of fixed-size random neighborhoods. An overall rejection rate is calculated after averaging the binary test results, with low rejection rates indicating well-mixed batches. We used the kBET function from kBET package[58] (v0.99.6), using 'k0=30' and 'do.pca=FALSE' on the batch labels. To ensure that a higher kBET score represents better batch removal, we subtracted the original score from 1 to give a final kBET score.

4.1.4 ASW

The silhouette width measures the relationship between the similarity of a point to its own cluster and the similarity of that point to the closest neighboring cluster. The score ranges from -1 to 1, where a score close to 1 represents that the point is properly clustered, while a score ≤0 represents poor correspondence to its own cluster. The average silhouette width (ASW) provides an evaluation of clustering validity. To compute ASW, we used the 'silhouette' function from the 'cluster' R package[59] (v.2.1.0). To compute cell type ASW and batch ASW, we followed the scaling and normalization established previously[5]. Specifically, cell type ASW (ASW^c) was normalized as (ASW^c+1)/2. To calculate the batch ASW, we first calculated the batch ASW (ASW^b) for each cell type j as:

$$\text{ASW}_j^b = \frac{1}{|C_j|} \sum_{i \in C_j} 1 - |s(i)|$$

$$\text{ASW}^b = \frac{1}{|M|} \sum_{j \in \text{M}} \text{ASW}_j^b$$

Here, s(i) is the silhouette width on batch labels for cell i, C_j represents the set of cells with cell labels j, and M is the set of unique cell labels.

4.1.5 ARI

The Adjusted Rand Index (ARI) is a measure of similarity between two data clusterings, corrected by chance. An ARI score of 1 represents perfect correspondence, while a value of 0 represents random labeling. To calculate ARI, we performed Louvain clustering using igraph[60] (v.1.3.4) to obtain cluster labels that were compared to the cell type labels. We followed the approach proposed previously[5], where the ARI was optimized based on iterating the clustering resolution from 0.1 to 2 in steps of 0.1. The final clustering was chosen based on the highest ARI value. To compute ARI, we used the function 'ARI' from the 'aricode' R package[61] (v.1.0.0).

4.1.6 NMI

Like ARI, the normalized mutual information (NMI) is a measure of similarity between two data clusterings. An NMI score of 1 represents perfect correspondence, while a value of 0 represents random labeling. To calculate the NMI, we

used the optimal clustering obtained from the optimization of the ARI score. To compute NMI, we used the function 'NMI' from the aricode package (v.1.0.0).

5 Simulating cohort-level scRNA-seq data

5.1 A generative model for simulating single-cell data

Our aim is to simulate cohort-level single-cell data in a way that, in the ground truth, condition-associated differential expression becomes a smooth function of cell state. In other words, cells that have similar biological states change in a similar manner in response to sample-level variables. We do this by simulating the cell state manifold using a set of "archetypes", similar to previous works[62, 63], with each archetype associated with a differential expression (DE) vector. Each cell is then simulated as a weighted average of these archetypes. The DE vector of each cell, then, becomes the weighted average of the DE vectors of the archetypes to which it is assigned.

We start by first describing how the expression vector of the archetypes are simulated. Consider the N cells in the dataset. We model each cell based on its soft assignment to K archetypes, where K is large enough to cover the diversity of cells across the manifold. Each of the K archetypes is expected to be assigned (with a certain weight) to cells with similar biological states, which may come from different samples.

Let's consider gene g. For each sample i, let's denote the (log-scale) expression of gene g across the K archetypes as the column vector $\boldsymbol{\gamma}_{g,i} \in \mathbb{R}^K$. We model this vector with a multivariate normal distribution whose mean is determined by the sample characteristics (i.e., column-vector $\mathbf{h}_i \in \mathbb{R}^L$, where L is the number of sample-level variables):

$$\boldsymbol{\gamma}_{g,i} \sim \mathcal{N}\left(\mathbf{X}_g \mathbf{h}_i + \mathbf{x}'_g \mathbf{h}_i \mathbf{1}_K, \boldsymbol{\Sigma}_g\right)$$

Here, $\mathbf{X}_g$ is a gene-specific matrix of coefficients ($\mathbf{X}_g \in \mathbb{R}^{K \times L}$), which shows, for each archetype (represented by each row of $\mathbf{X}_g$), how the mean expression of gene g is determined by each sample-level variable (represented by each column of $\mathbf{X}_g$). $\mathbf{x}'_g$ is a row vector ($\mathbf{x}'_g \in \mathbb{R}^L$), representing the global effects of sample-level variables on the expression of gene g irrespective of the archetype (and, by extension, cell state). For example, if different samples are generated by different technologies, there will be systematic differences in the capture efficiency of each gene (e.g., driven by gene length or GC content). Another source of such global differences can be the ambient RNA, which can be potentially associated with biological characteristics of the sample, but affects the observed UMI counts of all the cells uniformly[64]. $\mathbf{1}_K$ is a column-vector of 1's. $\boldsymbol{\Sigma}_g$ is a gene-specific covariance matrix, representing the covariance across the K archetypes conditional on sample-level variables ($\boldsymbol{\Sigma}_g \in \mathbb{R}^{K \times K}$). In other words, $\boldsymbol{\Sigma}_g$ represents the inter-sample variability that cannot be explained by sample-level variables, as well as the correlations of these residual variabilities across archetypes.

If $\mathbf{X}_g$, $\mathbf{x}'_g$, and $\boldsymbol{\Sigma}_g$ are given for all $g \in \{1,\ldots,G\}$, then for each sample i we can draw the mean expression of all genes across all K archetypes, given the sample characteristics $\mathbf{h}_i$, from the multivariate normal distribution above.

Next, we will describe how the expression vector of each individual cell can be simulated. Let's represent the weighted assignment of each cell n to the K archetypes using the row vector $\mathbf{w}_n \in \mathbb{R}_+^K$ ($\Sigma_k w_{n,k}=1$). The expression of gene g in cell n, i.e., $y_{g,n}$, is modeled as:

$$y_{g,n} \sim \mathcal{N}\left(\mathbf{w}_n \cdot \boldsymbol{\gamma}_{g,i(n)}, \sigma_g^2\right)$$

Here, σ_g is a gene-specific standard deviation representing the extent to which the expression of gene g in a cell may deviate from the respective weighted average of the archetypes. In other words, σ_g represents the within-sample variability of cells around the cell state manifold.

Finally, having sampled, from the distribution above, the ground truth expression of each gene in each cell, we simulate UMI counts as follows. First, we convert the log-scale expression vector of each cell to "gene proportion" values:

$$p_{g,n} = \frac{c^{y_{g,n}}}{\sum_{g=1}^{G} c^{y_{g,n}}}$$

$$\mathbf{p}_n = [p_{1,n} \quad \cdots \quad p_{G,n}]$$

Here, c is the logarithmic base for $y_{g,n}$, which is determined by the logarithmic scale used in $\boldsymbol{X}_g$. For example, if $\boldsymbol{X}_g$ contains the log2 fold-change of gene expression in each cluster, then c=2.

Then, we sample the UMI counts for each cell n using a multinomial distribution:

$$\mathbf{m}_n \sim \text{Multinom}_G(M_n, \mathbf{p}_n)$$

Here, M_n is a scalar representing the total number of UMI counts for cell n, and $\mathbf{m}_n$ is the vector of simulated UMI counts across the G genes ($\mathbf{m}_n \in \mathbb{Z}^G$).

Note that, since the expected expression vector of each cell in each sample is the weighted average of the expression vectors of the archetypes, the expected (ground truth) DE for each cell is also the weighted average of the DE in archetypes:

$$\boldsymbol{\delta}_{n,g} = \mathbf{w}_n \mathbf{X}_g$$

Here, $\boldsymbol{\delta}_{n,g}$ is a row vector ($\boldsymbol{\delta}_{n,g} \in \mathbb{R}^L$), representing how much the expression of gene g in cell n changes in response to a unit change in each of the L sample-level variables. For example, for the l'th sample-level variable, we can construct the following ground truth matrix:

$$\boldsymbol{\Delta}_l = \begin{bmatrix} (\boldsymbol{\delta}_{1,1})_l & \cdots & (\boldsymbol{\delta}_{N,1})_l \\ \vdots & \ddots & \vdots \\ (\boldsymbol{\delta}_{1,G})_l & \cdots & (\boldsymbol{\delta}_{N,G})_l \end{bmatrix}$$

This matrix can then be compared to inferences made by a clustering-free differential expression analysis tool, in order to understand the extent to which those inferences correlate with the ground truth for each cell (compare each column of $\boldsymbol{\Delta}_l$ to the inferences made for each cell across the G genes).

5.2 Selecting the parameters of the generative model

Together, the equations above allow us to construct a ground-truth expression vector for each cell as well as an observed UMI count vector, starting from the following parameters: $\mathbf{X}_g$, $\boldsymbol{\Sigma}_g$, and σ_g for all $g \in \{1,\ldots,G\}$, and $\mathbf{w}_n$ and M_n for all $n \in \{1,\ldots,N\}$. In this section, we will describe how these parameters can be chosen in order to resemble real biological datasets. Briefly, we start with a real cohort-level single-cell RNA-seq dataset, and use it as a template to roughly estimate model parameters that can explain the observed data. Then, we use these model parameters as ground truth to generate a simulated dataset as described in section 5.1. Note that we do not need to precisely estimate the parameters in a way that, for example, they maximize the likelihood of the real (template) dataset; our goal is simply to obtain a sensible set of values for the parameters of the generative model in a way that core properties, such as the gene-gene and cell-cell correlation structures, are reflected. Therefore, we use a heuristic approach to derive the parameters, as described below.

5.2.1 Estimating w_n

We use Harmony to integrate the single-cell data of different samples across the template dataset. Harmony performs integration by iterative "maximum diversity clustering" of the cells, which leads to soft clustering of the cells into K clusters. For each cell n, we use the soft clustering weights provided by Harmony as the $\mathbf{w}_n$ (for each cell, we normalize $\mathbf{w}_n$ so that the sum of the K elements are 1); in other words, we consider the weighted average of the cells of each cluster as an "archetype". In the simulations presented in this paper, we used K=100.

5.2.2 Estimating X_g and x'_g

The cells that are assigned by Harmony to each cluster can come from different samples. For each cluster k in each sample i, we create a pseudobulk as follows:

$$\mathbf{m}_{k,i} = \lfloor \mathbf{M}_i \mathbf{w}'_{k,i} \rfloor$$

Here, $\mathbf{M}_i$ is the UMI count matrix of sample i ($\mathbf{M}_i \in \mathbb{R}^{G \times N_i}$, where N_i is the number of cells in sample i), $\mathbf{m}_{k,i}$ is a column vector representing the pseudobulk UMI counts for cluster k in sample i, and $\lfloor x \rfloor$ denotes the floor function, ensuring that the elements of $\mathbf{m}_{k,i}$ are integers ($\mathbf{m}_{k,i} \in \mathbb{Z}^G$). $\mathbf{w}'_{k,i}$ is a column vector that contains the weights for soft assignment of each cell in sample i to cluster k:

$$\mathbf{w}'_{k,i} = \left[(\mathbf{w}_{n_1})_k \quad \cdots \quad (\mathbf{w}_{n_{N_i}})_k \right]$$

Here, n_1 to n_{N_i} are indices of the cells that belong to sample i. This procedure effectively results in taking the weighted average of the UMI counts. In the simulations presented in this paper, we removed pseudobulks that had at least 1

sample with less than 50 sum UMI counts across all genes, keeping K=67. We then renormalized $\mathbf{w}_n$ so that the sum of the K elements are 1.

Next, for each cluster k, we collate the column vectors $\mathbf{m}_{k,i}$ across all samples i (resulting in matrix $\mathbf{M'}_k \in \mathbb{R}^{G\times Q}$, where Q is the total number of samples in the dataset), followed by pseudobulk DE analysis, with $\mathbf{H}$ used as the design matrix ($\mathbf{H}$ is a $Q\times L$ matrix; each row i of $\mathbf{H}$ gives us the characteristics of sample i, which is the same as $\mathbf{h}_i^\top$). Here, we use DESeq2 for DE analysis, followed by retrieval of the model coefficients using the 'coef' function. Let's denote the matrix returned by the 'coef' function for the k'th cluster as $\mathbf{B}_k$ ($\mathbf{B}_k \in \mathbb{R}^{G\times L}$). For coefficients other than the intercept, we replace the values of the corresponding column in $\mathbf{B}_k$ with shrunken coefficients obtained using lfcShrink (type="apeglm"). This shrinkage provides a more sparse coefficient matrix; furthermore, for clusters in which certain condition groups are under-represented, the coefficients of those conditions is more strongly shrunken toward zero.

Next, for each gene g, we can construct $\mathbf{X}_g$ and $\mathbf{x'}_g$ as follows, using all $\mathbf{B}_k$ matrices of all K clusters. First, let's define the matrix $\mathbf{X'}_g$:

$$\mathbf{X}'_g = \begin{bmatrix} (\mathbf{B}_1)_{g,1} & \cdots & (\mathbf{B}_1)_{g,L} \\ \vdots & \ddots & \vdots \\ (\mathbf{B}_K)_{g,1} & \cdots & (\mathbf{B}_K)_{g,L} \end{bmatrix}$$

We set the row vector $\mathbf{x'}_g$ to be the column-wise median of $\mathbf{X'}_g$. Subsequently, we obtain $\mathbf{X}_g$ by subtracting $\mathbf{x'}_g$ from each row of $\mathbf{X'}_g$:

$$\mathbf{X}_g = \mathbf{X}'_g - \mathbf{x}'_g \mathbf{1}_K$$

Finally, we "trim" matrix $\mathbf{X}_g$ by replacing with zero the elements whose absolute value is below some threshold θ to create a more sparse ground truth. In the analyses presented in this paper, we set θ=0.3.

5.2.3 Estimating Σ_g:

Consider again, for each cluster k, the $G\times Q$ matrix of counts that was used to fit the DESeq2 pseudobulk model in the previous section (i.e., matrix $\mathbf{M'}_k$). We first use variance-stabilized transformation to obtain log-scale normalized values for each cluster k, which we denote here as $\hat{\mathbf{Y}}_k$ ($\hat{\mathbf{Y}}_k \in \mathbb{R}^{G\times Q}$). Then, for each gene g, we construct the matrix of residuals $\hat{\mathbf{Y}}'_g$ as follows ($\hat{\mathbf{Y}}'_g \in \mathbb{R}^{K\times Q}$):

$$\hat{\mathbf{Y}}'_g = \begin{bmatrix} (\hat{\mathbf{Y}}_1)_{g,1} & \cdots & (\hat{\mathbf{Y}}_1)_{g,Q} \\ \vdots & \ddots & \vdots \\ (\hat{\mathbf{Y}}_K)_{g,1} & \cdots & (\hat{\mathbf{Y}}_K)_{g,Q} \end{bmatrix} - \mathbf{X}'_g \mathbf{H}^\top$$

The matrix $\boldsymbol{\Sigma}_g$ for gene g can then be constructed by simply taking the covariance of rows of $\hat{\mathbf{Y}}'_g$.

5.2.4 Estimating σ_g:

We start by log-scale normalization of UMI counts in each cell of each sample i. Let's denote the log-scale normalized count matrix for sample i as $\mathbf{V'}_i$ ($\mathbf{V'}_i \in \mathbb{R}^{G\times Ni}$). We also construct the matrix of normalized pseudobulk expression values for sample i across the K clusters (archetypes), reusing matrices $\hat{\mathbf{Y}}_k$ from the previous section, followed by intrapolation of these values to obtain the projected expression profile of each cell on the sample manifold:

$$\mathbf{U}_i = \begin{bmatrix} (\hat{\mathbf{Y}}_1)_{1,i} & \cdots & (\hat{\mathbf{Y}}_K)_{1,i} \\ \vdots & \ddots & \vdots \\ (\hat{\mathbf{Y}}_1)_{G,i} & \cdots & (\hat{\mathbf{Y}}_1)_{G,i} \end{bmatrix} \mathbf{W}_i$$

Here, $\mathbf{W}_i$ is the matrix of cluster-by-cell weights ($\mathbf{W}_i \in \mathbb{R}_+^{K\times Ni}$):

$$\mathbf{W}_i = \begin{bmatrix} \mathbf{w}_{n_1}^\top & \cdots & \mathbf{w}_{n_{N_i}}^\top \end{bmatrix}$$

Again, n_1 to n_{Ni} are indices of the cells that belong to sample i.
We obtain the matrix of residuals $\mathbf{V''}_i$ ($\mathbf{V''} \in \mathbb{R}^{G\times N}$) by individually performing ordinary least squares regression of each column of $\mathbf{V'}_i$ (dependent variable) against the corresponding column of $\mathbf{U}_i$ (independent variable), and collating all matrices $\mathbf{V''}_i$ across all samples:

$$\mathbf{V}'' = [\mathbf{V}''_1 \ \cdots \ \mathbf{V}''_Q]$$

Finally, for each gene g, σ_g is estimated as the standard deviation of the elements of the g'th row of $\mathbf{V''}$.

5.2.5 Estimating M_n:

For each cell n in the template dataset, we generate a corresponding cell in the simulated dataset as described in section 5.1. We simply use the sum of UMI counts of cell n in the template dataset as M_n in the simulated dataset.

5.3 Cluster-free differential expression benchmark

Differential expression estimates from each method were compared to the ground truth vectors of the simulated data, by either calculating the Pearson correlation or by assessing the classification of up-regulated or down-regulated genes using AUROC values. Sets of up-regulated and down-regulated genes were defined by using a threshold of 0.3 (log_2 scale) of the ground truth DE values. AUROC values were calculated using the roc function from pROC (v.1.18). For the clustering free DGE methods, we employed 20 latent variables during the dimensional reduction steps. Unless specified, we used the default parameters established by each package. The details of each method can be found below:

5.3.1 GEDI

GEDI was run using the raw counts, specifying a sample-level metadata and setting 'oi_shrinkage=0.001'. Single-cell differential expression estimates were retrieved using the function 'getDiffExp.gedi'.

5.3.2 LEMUR

To run LEMUR[65] (v.1.0.4), we followed the documentation available on the Bioconductor vignette[66]. We used a SingleCellExperiment object with log-normalized counts as input to the lemur function, using n_embedding=20. We then applied the align_harmony and test_de functions. Single-cell differential expression estimates were retrieved by accessing the "DE" slot of the LEMUR object.

5.3.3 miloDE

To run miloDE[67] (v.0.0.0.9000), we followed the documentation available on its GitHub repository[68]. We used scVI for the calculation of the latent embeddings, which are required as input for the generation of the nearest neighbor graph. Assignation of neighborhoods was performed using the assign_neighbourhoods function, followed by differential expression testing using the de_test_neighbourhood function. As miloDE returns differential expression estimates by neighborhood, we collapsed GEDI's and LEMUR's single-cell DE estimates into neighborhood estimates by averaging across cells of miloDE neighborhoods in order to be able to compare their performances.

5.3.4 Vector field visualization

First, a low dimensional representation of the vector field was retrieved by applying SVD using GEDI's svd.vectorField.gedi function. This function first computes the differences between the manifold of two conditions, defined as start and end by the user. Then, it performs SVD to return a projection of the cells on the manifold for the start and end conditions. Next, it applies UMAP to the SVD embedding of the vector field, retrieving a two dimensional space projection. Finally, the vector field is plotted using GEDI's function plot_vectorField, which receives the UMAP coordinates and connects the change between the start and end conditions using an arrow. For a less cluttered visualization, this function groups the cells that are near each other in the UMAP embedding and plots the average of their associated vectors.

Supplementary References

1. Ding, J. et al. Systematic comparison of single-cell and single-nucleus RNA-sequencing methods. *Nat Biotechnol* **38**, 737-746 (2020).
2. Segerstolpe, A. et al. Single-Cell Transcriptome Profiling of Human Pancreatic Islets in Health and Type 2 Diabetes. *Cell Metab* **24**, 593-607 (2016).
3. Baron, M. et al. A Single-Cell Transcriptomic Map of the Human and Mouse Pancreas Reveals Inter- and Intra-cell Population Structure. *Cell Syst* **3**, 346-360 e344 (2016).
4. Tabula Muris, C. et al. Single-cell transcriptomics of 20 mouse organs creates a Tabula Muris. *Nature* **562**, 367-372 (2018).
5. Luecken, M.D. et al. Benchmarking atlas-level data integration in single-cell genomics. *Nat Methods* **19**, 41-50 (2022).
6. He, L. et al. NEBULA is a fast negative binomial mixed model for differential or co-expression analysis of large-scale multi-subject single-cell data. *Commun Biol* **4**, 629 (2021).
7. Schulte-Schrepping, J. et al. Severe COVID-19 Is Marked by a Dysregulated Myeloid Cell Compartment. *Cell* **182**, 1419-1440 e1423 (2020).
8. Genga, R.M.J. et al. Single-Cell RNA-Sequencing-Based CRISPRi Screening Resolves Molecular Drivers of Early Human Endoderm Development. *Cell Rep* **27**, 708-718 e710 (2019).
9. Kuleshov, M.V. et al. Enrichr: a comprehensive gene set enrichment analysis web server 2016 update. *Nucleic Acids Res* **44**, W90-97 (2016).
10. Tasic, B. et al. Adult mouse cortical cell taxonomy revealed by single cell transcriptomics. *Nat Neurosci* **19**, 335-346 (2016).
11. Tasic, B. et al. Shared and distinct transcriptomic cell types across neocortical areas. *Nature* **563**, 72-78 (2018).
12. Korotkevich, G. et al. Fast gene set enrichment analysis. *bioRxiv*, 060012 (2021).
13. van Dijk, D. et al. Recovering Gene Interactions from Single-Cell Data Using Data Diffusion. *Cell* **174**, 716-729 e727 (2018).
14. Huang, M. et al. SAVER: gene expression recovery for single-cell RNA sequencing. *Nat Methods* **15**, 539-542 (2018).
15. Gaidatzis, D., Burger, L., Florescu, M. & Stadler, M.B. Analysis of intronic and exonic reads in RNA-seq data characterizes transcriptional and post-transcriptional regulation. *Nature biotechnology* **33**, 722-729 (2015).
16. Faure, L. et al. Single cell RNA sequencing identifies early diversity of sensory neurons forming via bi-potential intermediates. *Nat Commun* **11**, 4175 (2020).
17. La Manno, G. et al. RNA velocity of single cells. *Nature* **560**, 494-498 (2018).
18. Cheng, L.-C., Pastrana, E., Tavazoie, M. & Doetsch, F. miR-124 regulates adult neurogenesis in the subventricular zone stem cell niche. *Nature neuroscience* **12**, 399-408 (2009).
19. Korsunsky, I. et al. Fast, sensitive and accurate integration of single-cell data with Harmony. *Nat Methods* **16**, 1289-1296 (2019).
20. https://singlecell.broadinstitute.org/single_cell/study/SCP424/single-cell-comparison-pbmc-data#study-summary (Accessed on 2021-09-01)
21. https://github.com/satijalab/seurat-data (Accessed on 2021-09-01)
22. https://bioconductor.org/packages/release/bioc/html/batchelor.html (Accessed on 2022-01-01)

23. Haghverdi, L., Lun, A.T.L., Morgan, M.D. & Marioni, J.C. Batch effects in single-cell RNA-sequencing data are corrected by matching mutual nearest neighbors. *Nat Biotechnol* **36**, 421-427 (2018).

24. https://figshare.com/articles/dataset/Processed_files_to_use_with_scanpy_/8273102 (Accessed on 2021-04-20)

25. https://beta.fastgenomics.org/datasets/detail-dataset-952687f71ef34322a850553c4a24e82e#Files (Accessed on 2022-03-16)

26. https://beta.fastgenomics.org/datasets/detail-dataset-7ae02f5553074bda92c14a8f0bce2d24#Files (Accessed on 2022-03-16)

27. https://github.com/LouisFaure/sensoryfates_paper (Accessed on 2022-02-20)

28. http://pklab.med.harvard.edu/velocyto/hgForebrainGlut/ (Accessed on 2022-09-02)

29. https://doi.org/10.5281/zenodo.3564179 (Accessed on 2023-01-17)

30. Holland, C.H. et al. Robustness and applicability of transcription factor and pathway analysis tools on single-cell RNA-seq data. *Genome Biol* **21**, 36 (2020).

31. Feng, H. et al. Complexity and graded regulation of neuronal cell-type-specific alternative splicing revealed by single-cell RNA sequencing. *Proc Natl Acad Sci U S A* **118** (2021).

32. https://zhanglab.c2b2.columbia.edu/index.php/Quantas_Documentation (Accessed on 2022-07-28)

33. https://zhanglab.c2b2.columbia.edu/index.php/OLego (Accessed on 2022-07-28)

34. Wu, J., Anczukow, O., Krainer, A.R., Zhang, M.Q. & Zhang, C. OLego: fast and sensitive mapping of spliced mRNA-Seq reads using small seeds. *Nucleic Acids Res* **41**, 5149-5163 (2013).

35. https://cran.r-project.org/package=Seurat (Accessed on 2022-07-23)

36. Hao, Y. et al. Integrated analysis of multimodal single-cell data. *Cell* **184**, 3573-3587 e3529 (2021).

37. https://satijalab.org/seurat/ (Accessed on 2022-07-23)

38. Welch, J.D. et al. Single-Cell Multi-omic Integration Compares and Contrasts Features of Brain Cell Identity. *Cell* **177**, 1873-1887 e1817 (2019).

39. https://github.com/welch-lab/liger (Accessed on 2022-07-23)

40. https://cran.r-project.org/package=harmony (Accessed on 2022-03-09)

41. https://github.com/immunogenomics/harmony (Accessed on 2022-03-09)

42. Polanski, K. et al. BBKNN: fast batch alignment of single cell transcriptomes. *Bioinformatics* **36**, 964-965 (2020).

43. https://github.com/Teichlab/bbknn (Accessed on 2022-07-27)

44. https://scanpy.readthedocs.io/ (Accessed on 2022-07-27)

45. He, Z., Brazovskaja, A., Ebert, S., Camp, J.G. & Treutlein, B. CSS: cluster similarity spectrum integration of single-cell genomics data. *Genome Biol* **21**, 224 (2020).

46. https://github.com/quadbio/simspec (Accessed on 2022-03-10)

47. https://cran.r-project.org/package=rsvd (Accessed on 2022-07-23)

48. Lopez, R., Regier, J., Cole, M.B., Jordan, M.I. & Yosef, N. Deep generative modeling for single-cell transcriptomics. *Nat Methods* **15**, 1053-1058 (2018).

49. https://docs.scvi-tools.org/en/stable/ (Accessed on 2024-03-01)

50. Hie, B., Bryson, B. & Berger, B. Efficient integration of heterogeneous single-cell transcriptomes using Scanorama. *Nat Biotechnol* **37**, 685-691 (2019).

51. https://github.com/brianhie/scanorama (Accessed on 2024-03-01)

52. McInnes, L., Healy, J. & Melville, J. Umap: Uniform manifold approximation and projection for dimension reduction. *arXiv preprint arXiv:1802.03426* (2018).

53. Moon, K.R. et al. Visualizing structure and transitions in high-dimensional biological data. *Nat Biotechnol* **37**, 1482-1492 (2019).

54. https://cran.r-project.org/package=uwot (Accessed on 2022-07-01)

55. Butler, A., Hoffman, P., Smibert, P., Papalexi, E. & Satija, R. Integrating single-cell transcriptomic data across different conditions, technologies, and species. *Nat Biotechnol* **36**, 411-420 (2018).

56. https://github.com/immunogenomics/LISI (Accessed on 2022-03-09)

57. Buttner, M., Miao, Z., Wolf, F.A., Teichmann, S.A. & Theis, F.J. A test metric for assessing single-cell RNA-seq batch correction. *Nat Methods* **16**, 43-49 (2019).

58. https://github.com/theislab/kBET (Accessed on 2022-07-01)

59. https://cran.r-project.org/package=cluster (Accessed on 2022-08-01)

60. https://cran.r-project.org/package=igraph (Accessed on 2022-08-01)

61. https://cran.r-project.org/package=aricode (Accessed on 2022-08-01)

62. Korem, Y. et al. Geometry of the Gene Expression Space of Individual Cells. *PLoS Comput Biol* **11**, e1004224 (2015).

63. Persad, S. et al. SEACells infers transcriptional and epigenomic cellular states from single-cell genomics data. *Nat Biotechnol* **41**, 1746-1757 (2023).

64. Fleming, S.J. et al. Unsupervised removal of systematic background noise from droplet-based single-cell experiments using CellBender. *Nat Methods* **20**, 1323-1335 (2023).

65. Ahlmann-Eltze, C. & Huber, W. Analysis of multi-condition single-cell data with latent embedding multivariate regression. *bioRxiv*, 2023.2003.2006.531268 (2023).

66. https://bioconductor.org/packages/release/bioc/vignettes/lemur/inst/doc/Introduction.html (Accessed on 2023-12-01)

67. Missarova, A., Dann, E., Rosen, L., Satija, R. & Marioni, J. Sensitive cluster-free differential expression testing. *bioRxiv*, 2023.2003.2008.531744 (2023).

68. https://github.com/MarioniLab/miloDE (Accessed on 2023-12-11)
