# Supplementary material for: A unified model for interpretable latent embedding of multi-sample, multi-condition single-cell data
Source: Nat Commun. 2024 Aug 3;15:6573. doi: 10.1038/s41467-024-50963-0 (PMC11298001; doi:10.1038/s41467-024-50963-0)
Supplement: Supplementary file 3 — Description of Additional Supplementary Files [file 41467_2024_50963_MOESM3_ESM.pdf]

Description of Additional Supplementary Files:

Supplementary Data 1: Integration metrics for different methods across different datasets.

Supplementary Data 2: AUROC values for differential enrichment of transcription factor activities in the PBMC dataset.
