# Supplementary material for: A unified model for interpretable latent embedding of multi-sample, multi-condition single-cell data
Source: Nat Commun. 2024 Aug 3;15:6573. doi: 10.1038/s41467-024-50963-0 (PMC11298001; doi:10.1038/s41467-024-50963-0)
Supplement: Supplementary file 6 — Reporting Summary [file 41467_2024_50963_MOESM6_ESM.pdf]

nature portfolio

Corresponding author(s): Hamed S. Najafabadi

Last updated by author(s): Jul 9, 2024

Reporting Summary

Nature Portfolio wishes to improve the reproducibility of the work that we publish. This form provides structure for consistency and transparency in reporting. For further information on Nature Portfolio policies, see our Editorial Policies and the Editorial Policy Checklist.

Statistics

For all statistical analyses, confirm that the following items are present in the figure legend, table legend, main text, or Methods section.

n/a	Confirmed	
☐	☒	The exact sample size (n) for each experimental group/condition, given as a discrete number and unit of measurement
☒	☐	A statement on whether measurements were taken from distinct samples or whether the same sample was measured repeatedly
☐	☒	The statistical test(s) used AND whether they are one- or two-sided<br>*Only common tests should be described solely by name; describe more complex techniques in the Methods section.*
☐	☒	A description of all covariates tested
☒	☐	A description of any assumptions or corrections, such as tests of normality and adjustment for multiple comparisons
☐	☒	A full description of the statistical parameters including central tendency (e.g. means) or other basic estimates (e.g. regression coefficient) AND variation (e.g. standard deviation) or associated estimates of uncertainty (e.g. confidence intervals)
☐	☒	For null hypothesis testing, the test statistic (e.g. F, t, r) with confidence intervals, effect sizes, degrees of freedom and P value noted<br>*Give P values as exact values whenever suitable.*
☐	☒	For Bayesian analysis, information on the choice of priors and Markov chain Monte Carlo settings
☒	☐	For hierarchical and complex designs, identification of the appropriate level for tests and full reporting of outcomes
☐	☒	Estimates of effect sizes (e.g. Cohen's d, Pearson's r), indicating how they were calculated

Our web collection on statistics for biologists contains articles on many of the points above.

Software and code

Policy information about availability of computer code

Data collection	Raw count matrices and metadata from the PBMC dataset and Pancreas datasets were downloaded from the SeuratData(v.0.1) R package. For the PBMC dataset, data was retrieved using the 'pbmcsca' (v.3.0.0). For the Pancreas dataset, we used the accession code 'panc8' (v.3.0.2). Other datasets used in the article were downloaded from the publication access codes or from their online data repositories.
Data analysis	Quality control and preprocessing steps were performed with the scuttle package (v 1.0.4). Normalization of single cell data was performed using batchelor (v.1.6.3). For pre-processing of exon inclusion and exclusion events, we used Olego (v.1.1.9) and the Quantas pipeline (v.1.1.1). For integration benchmarking analysis, we used the following packages: Seurat (v.4.1.1), LIGER (v.1.0.0), Harmony (v.1.0), BBKNN (v.1.3.12), CSS (v.0.0.0.9000), scVI (v.1.0.4) and Scanorama (v.1.7.4). Pseudo-bulk differential expression analysis was performed using DESeq2 (v.30.1). Marker analysis was performed using scran (v.1.30.0). Differential expression analysis of imputed logit of PSI was computed using limma (v.3.46). Sashimi plots were generated using sashimipy (v 0.0.6). Estimation of TF activities was performed using decoupleR (v.2.8.0). For cluster-free differential expression, we used LEMUR (v.1.0.4) and miloDE (v.0.0.0.9000).<br><br>GEDI is available at: https://github.com/csglab/GEDI. Notebooks with reproducible analysis are available at: https://github.com/csglab/GEDI_manuscript

For manuscripts utilizing custom algorithms or software that are central to the research but not yet described in published literature, software must be made available to editors and reviewers. We strongly encourage code deposition in a community repository (e.g. GitHub). See the Nature Portfolio guidelines for submitting code & software for further information.

Data

Policy information about availability of data

All manuscripts must include a data availability statement. This statement should provide the following information, where applicable:

- Accession codes, unique identifiers, or web links for publicly available datasets
- A description of any restrictions on data availability
- For clinical datasets or third party data, please ensure that the statement adheres to our policy

Only publicly available datasets were used in this article:
PBMC dataset (https://doi.org/10.1038/s41587-020-0465-8). Retrieved using the SeuratData package (v0.1), using the accession code 'pbmcsca' (v.3.0.0). Also available at GEO accession number GSE132044.
Pancreas dataset. (https://doi.org/10.1016/j.cmet.2016.08.020; https://doi.org/10.1016/j.cels.2016.08.011) Retrieved using the SeuratData package (v0.1), using the accession code 'panc8' (v.3.0.2). Also available at ArrayExpress accession number E-MTAB-5061 and at GEO accession number GSE84133.
Tabula Muris Bone Marrow dataset (https://doi.org/10.1038/s41586-018-0590-4): Downloaded from the publication repository at :https://figshare.com/articles/dataset/Processed_files_to_use_with_scanpy_/8273102
COVID-19 dataset (https://doi.org/10.1016/j.cell.2020.08.001). Downloaded from the FastGenomic portal https://beta.fastgenomics.org/datasets/detail-dataset-952687f71ef34322a850553c4a24e82e#Files and https://beta.fastgenomics.org/datasets/detail-dataset-7ae02f5553074bda92c14a8f0bce2d24#Files. The data is also deposited at the European Genome-phenome Archive (EGA) under access number EGAS00001004571.
Tasic dataset (https://doi.org/10.1038/nn.4216; https://doi.org/10.1038/s41586-018-0654-5): Downloaded from the SRA accession numbers SRP061902 and SRP150473 and GEO accession number GSE115746.
Faure dataset (https://doi.org/10.1038/s41467-020-17929-4): Downloaded from the GEO accession number GSE150150.
LaManno dataset (https://doi.org/10.1038/s41586-018-0414-6). Downloaded from author's repository: http://pklab.med.harvard.edu/velocyto/hgForebrainGlut/.
Genga dataset (https://doi.org/10.1016/j.celrep.2019.03.076) downloaded from Zenodo https://zenodo.org/doi/10.5281/zenodo.3564178.

GEDI models can be accessed via Zenodo (DOIs: 10.5281/zenodo.8222039, 10.5281/zenodo.8222697, 10.5281/zenodo.11163741, and 10.5281/zenodo.11164776).
Source data are provided with this paper.

Research involving human participants, their data, or biological material

Policy information about studies with human participants or human data. See also policy information about sex, gender (identity/presentation), and sexual orientation and race, ethnicity and racism.

Reporting on sex and gender	No human data was collected in this study.
Reporting on race, ethnicity, or other socially relevant groupings	No human data was collected in this study.
Population characteristics	No human data was collected in this study.
Recruitment	No human data was collected in this study.
Ethics oversight	No human data was collected in this study.

Note that full information on the approval of the study protocol must also be provided in the manuscript.

Field-specific reporting

Please select the one below that is the best fit for your research. If you are not sure, read the appropriate sections before making your selection.

☒ Life sciences ☐ Behavioural & social sciences ☐ Ecological, evolutionary & environmental sciences

For a reference copy of the document with all sections, see nature.com/documents/nr-reporting-summary-flat.pdf

Life sciences study design

All studies must disclose on these points even when the disclosure is negative.

Sample size	No samples were collected in this study. No statistical method was used to predetermine sample size. For the analysis of publicly available data, sample sizes were determined based on prior studies performed in the field. The number of replicates for statistical analyses are specified in the figure legends.
Data exclusions	Unless specified, cells were removed from the downloaded datasets based on standard quality control criteria (% of mitochondrial reads < 20 %; number of UMI < 1000 and number of detected genes < 1000).
Replication	Computational experiments and analysis are reproducible using the notebooks and code provided.

Randomization	No samples were collected, therefore not applicable.
Blinding	No samples were collected, therefore not applicable.

Reporting for specific materials, systems and methods

We require information from authors about some types of materials, experimental systems and methods used in many studies. Here, indicate whether each material, system or method listed is relevant to your study. If you are not sure if a list item applies to your research, read the appropriate section before selecting a response.

Materials & experimental systems

n/a	Involved in the study
☒	☐ Antibodies
☒	☐ Eukaryotic cell lines
☒	☐ Palaeontology and archaeology
☒	☐ Animals and other organisms
☒	☐ Clinical data
☒	☐ Dual use research of concern
☒	☐ Plants

Methods

n/a	Involved in the study
☒	☐ ChIP-seq
☒	☐ Flow cytometry
☒	☐ MRI-based neuroimaging
